# Divergence and evolution of cotton bHLH proteins from diploid to allotetraploid

**DOI:** 10.1186/s12864-018-4543-y

**Published:** 2018-02-23

**Authors:** Bingliang Liu, Xueying Guan, Wenhua Liang, Jiedan Chen, Lei Fang, Yan Hu, Wangzhen Guo, Junkang Rong, Guohua Xu, Tianzhen Zhang

**Affiliations:** 1 National Key Laboratory of Crop Genetics and Germplasm Enhancement, Cotton Hybrid R & D Engineering Center (the Ministry of Education), Nanjing Agricultural University, Nanjing, 210095 China; 2 Institute of Engineering and Technology in Rice Industry, Yangzhou University, Yangzhou, 225007 China; 3 Institute of Food Crops, Jiangsu Academy of Agricultural Sciences, Jiangsu High Quality Rice R&D Center, Nanjing, Jiangsu 210014 China; 4 The Key Laboratory for Quality Improvement of Agricultural Products of Zhejiang Province, School of Agriculture and Food Science, Zhejiang A&F University, Linan, Hangzhou, Zhejiang 311300 China; 5 MOA Key Laboratory of Plant Nutrition and Fertilization in Lower-Middle Reaches of the Yangtze River, Nanjing Agricultural University, Nanjing, 210095 China

**Keywords:** bHLH protein, Orthologous genes, Duplicated pairs, Expansion, Divergent expression

## Abstract

**Background:**

Polyploidy is considered a major driving force in genome expansion, yielding duplicated genes whose expression may be conserved or divergence as a consequence of polyploidization.

**Results:**

We compared the genome sequences of tetraploid cotton (*Gossypium hirsutum*) and its two diploid progenitors, *G. arboreum* and *G. raimondii*, and found that the *bHLH* genes were conserved over the polyploidization. Oppositely, the expression of the homeolgous gene pairs was diversified. The biased homeologous proportion for *bHLH* family is significantly higher (64.6%) than the genome wide homeologous expression bias (40%). Compared with *cacao* (*T. cacao*), orthologous genes only accounted for a small proportion (41.7%) of whole cotton *bHLHs* family. The further Ks analysis indicated that *bHLH* genes underwent at least two distinct episodes of whole genome duplication: a recent duplication (1.0–60.0 million years ago, MYA, 0.005 < Ks < 0.312) and an old duplication (> 60.0 MYA, 0.312 < Ks < 3.0). The old duplication event might have played a key role in the expansion of the *bHLH* family. Both recent and old duplicated pairs (68.8%) showed a divergent expression profile, indicating specialized functions. The expression diversification of the duplicated genes suggested it might be a universal feature of the long-term evolution of cotton.

**Conclusions:**

Overview of cotton bHLH proteins indicated a conserved and divergent evolution from diploids to allotetraploid. Our results provided an excellent example for studying the long-term evolution of polyploidy.

**Electronic supplementary material:**

The online version of this article (10.1186/s12864-018-4543-y) contains supplementary material, which is available to authorized users.

## Background

Polyploidization (or whole-genome duplication, WGD) is a widespread speciation mechanism, particularly in plants [1]. It is estimated that 50% to 80% of angiosperms are polyploids [2, 3]. Polyploidy introduces genome-wide genetic redundancy and is considered as a major driving force in angiosperm evolution [4]. In general, WGD events are now believed to have occurred at least twice during the evolution of all angiosperms (ζ and ε WGD events, 319 and 192 million years ago, MYA) [4]. Global analysis with the assembled genomes and the collections of expressed sequence tags (EST) in multiple plant species also infer more recent genomic duplication events in eudicots (ϒ, 130 MYA) [5, 6] and in monocots (σ and ρ, 130 MYA and 60 MYA) [7, 8]. Relatively recent WGD, also known as neopolyploid events (50 MYA) are also well known and characterized in many crop plants including wheat, tobacco, Brassica, apple, banana, sugar cane, and cotton [9]. These species provide a valuable model for studying the long-term evolution of polyploidy.

The duplication of all genes in a genome is the most obvious consequence of polyploidization. Genes duplicated by polyploidy usually showed expression bias or divergence [10, 11]. Therefore, studying the subsequent fate of these duplicated gene pairs from each genome is particularly important for understanding the evolution of polyploidy [12]. Because the homoeologous genes may have redundant functions in the merged genome in one nuclei, redundant genes may be subjected to several evolutionary outputs. The common observations of the redundant gene fate include gene loss [13], neofunctionalization [14], subfunctionalization [14]. Usually, functional divergence can occur by neofunctionalization and subfunctionalization. The incidence of functional divergence among the duplicated genes is difficult to quantify because genes exert their biological roles in many different ways. Some gene products are part of subcellular structures, others engage in protein-protein interactions, network with DNA or RNA fragments, or catalyze the transformation of small molecules. Moreover, genes with the same biochemical functions may be expressed at different special and temporal patterns. Because the integration of the multiple aspects of gene functionality is very complex, it is impossible to summarize them with a single manner [13].

The cotton lineage experienced a five to six fold ploidy increase shortly after its divergence from the ancestor shared with *Theobroma cacao* at least 60 MYA [15]. Allotetraploid, such as *Gossypium hirsutum* (upland cotton, AADD) were derived from the interspecific hybridization of its two diploid ancestors. The A and D diploid cotton genome species diverged from a common ancestor 5–10 MYA. The polyploidization of the closest extant progenitors, *G. herbaceum* (A1) or *G. arboreum* (A2) and *G. raimondii* (D5), occurred 1–1.5 MYA [16]. The complete genome sequences of *G. hirsutum acc.*TM-1 [17] and two progenitor diploids [15, 18] provide raw information of all genes and proteins for the analysis of basic/helix-loop-helix (*bHLH*) gene family and its evolution in detail. The *bHLH* gene family was estimated to contain 214 members in *G. raimondii* genome [15]. The *bHLH* genes in cotton genome play important roles in critical fiber trait determination and cell developmental regulation such as fiber cell elongation [19] and glandular cell differentiation [20]. *Gossypium* genus experienced paleopolyploidy, diversification and diploids to allotetroploid, which provide an excellent model to investigate the history of *bHLH* genes in polyploidy process. Previous studies mainly focused on genome-wide identification, classification or expression analyses of *bHLH* genes [21–24], but little was involved in complete sets of duplication or functional divergence of a polyploidy genome and representatives of its ancestral diploid progenitor species (such as *G. herbaceum* (A1) and *G. raimondii* (D5)). Using the signature bHLH domain, systematical analyses were carried on the *bHLH* gene family in diploid and allotetraploid cotton genomes in the present work. Here, we integrated the sequence variations and expression profiles of *bHLH* gene members in diploids and allotetraploid cotton genome and a greater number of homoeologous expression biases appeared in allotetraploid cotton. In addition, an expansion of the *bHLH* family was also observed and the results suggested that the old duplications mainly contributed to the expansion of the *bHLH* family. Moreover, divergent expression of the recent and old duplicated pairs indicated a functional divergence. Our data provide a comprehensive study on gene evolution in WGD process.

## Results

### bHLH proteins in allotetraploid cotton and its diploid progenitors

The genome sequences of *G. arboreum* [18], *G. raimondii* [15] and *G. hirsutum* [17] provide us the possibility to conduct a genome-wide screen of the bHLH proteins. The *bHLH* genes identified in two diploids (*G. arboreum* and *G. raimondii*) are presented in Table 1 and Additional files 1, 2 and 3. Total number of bHLH proteins in *G. hirsutum* (432) is approximately equal to the sum of those in *G. arboreum* (215 *GabHLHs*) and *G. raimondii* (224 *GrbHLHs*)*.* Some animal bHLH proteins such as SREBP miss Arg-16 in their bHLH domain, but they can also bind to the E-box based on a hypothetical consensus motif [24–26]. In bHLH family, *GabHLH173*, *GrbHLH091*, *GhbHLH138* and *GhbHLH357* were considered part of this category, though lacking Arg16. Only five bHLH coding genes, *GhbHLH1*, *GhbHLH4*, *GhbHLH130*, *GhDEL61* and *GhDEL65* in *G. hirsutum*, had been identified previously [27–29]. Compared with *Oryza sativa* (178), *Arabidopsis thaliana* (170) [30], *Theobroma cacao* (94) [31] and *Vitis vinifera* (84) [32], the cotton *bHLH* gene family was one of the largest families identified by now.

**Table 1:** Predicted DNA binding characteristics based on the amino acid sequences of the bHLH domain of cotton bHLH proteins

Predicted DNA-binding Activities	Predicted Domain	No.in Gh	No.in Ga	No.in Gr
E-box but non-G-box binding	bHLH	60	28	33
G-box binding	bHLH	258	127	132
Non-E-box binding	bHLH	67	35	40
Non-DNA binding	HLH	47	25	19
Total	–	432	215	224

There are 202 ortholog gene pairs between *G. arboreum* (AA) and *G. hirsutum* A-subgenome, while 216 orthologous gene pairs were detected between *G. raimondii* (DD) and *G. hirsutum* D-subgenome. Also, 189 homoeologous gene pairs were detected between A- and D-subgenome in *G. hirsutum* (Additional file 4). Orthologous and homoeologous gene analysis between cottons is another evidence to verify a conservative evolution from diploids to allotetraploid.

Based on a neighbor-joining (NJ) phylogenetic tree, 871 bHLH proteins were subdivided into 31 subfamilies and 25 remaining proteins were classified as “orphans” because of low statistical support (Additional file 5). As shown in the phylogenetic tree, most of the ortholog genes between two diploids and allotetraploid were clustered into a same clade. The phylogenetic tree provides a way to estimate the number of *bHLH* genes in the most recent common ancestor (Additional file 6). Branches with only *GabHLH* (13) or *GrbHLH* (8) without *GhbHLH* members were likely to have experienced a gene loss or sequence divergence event during polyploidization, as found in our ortholog analysis between diploid and allotetraploid (Additional file 4).

### Orthologous genes analyses between *T.cacao* and cottons

After its divergence from *Theobroma cacao* at least 60 MYA, the cotton lineage experienced a five- to six-fold ploidy increase [33]. The number of *bHLH* genes in *G. hirsutum* is ~ 4.6 fold that in cacao. A similar state was also observed in *G. arboreum* (~ 2.3 fold greater) and *G. raimondii* (~ 2.4 fold greater). The topology of the *TcbHLH*, *GabHLH* and *GrbHLH* tree supported the possibility of duplications (Additional file 6), but not all triplications in the divergence from cacao [15]. This might result from gene loss during or after the triplications or duplications. Even within this data set, we also observed that the ratio of *GhbHLH* to *TcbHLH* genes in some subfamilies was up to 8:1 (Additional file 6), possibly due to the duplication. However, whole genome duplication (WGD) can only explain some of the *bHLH* gene family expansion observed.

The orthologous groups between *T. cacao* and three cotton species (*G. arboreum*, *G. raimondii,* and *G. hirsutum*) were also clustered separately by Inparanoid_4.1 (http://software.sbc.su.se/cgi-bin/request.cgi?project=inparanoid). The congruence observed between the trees (Additional file 6) and orthologous analysis suggest that our identification of the bHLH phylogeny is essentially correct. Here, we identified 74 orthologous groups between *T. cacao* and *G. arboreum bHLH* genes, corresponding to 68 orthologous groups between *T. cacao* and *G. raimondii*, whereas the orthologous genes that evolved from *T. cacao* were much the same. In addition, 71 and 77 orthologous groups were identified between *T. cacao* and the A- (87 genes) and D-subgenomes (93 genes) *bHLH* genes in allotetraploid cotton, respectively, accounting for 41.7% (87 + 93/432). We also identified 202 and 216 orthologous genes respectively between the A genome and A-subgenome, and the D genome and the D-subgenome of the allotetraploid. But there were 13 *GabHLH* genes and 8 *GrbHLH* genes lost in the evolution from diploid to allotetraploid.

### Conserved characteristics between diploids and the allotetraploid

The multiple sequence alignments were performed to examine the signature bHLH domain in these three cotton species (Additional file 7). On average, the basic regions (the N-terminal 17 positions) of the *GabHLH* and *GrbHLH* domains had 5.74 and 5.96 basic residues, respectively, although some of these proteins did not have a basic region at all. However, when two diploid ancestors united and formed an allotetraploid, the average number of basic residues in *GhbHLH* changed to 5.86. As expected, the number of basic residues was similar between the A-subgenome in *G. hirsutum* (5.87 average residues) and the A-genome in *G. arboreum* (t-test, *p* < 0.05), also no difference between the D-subgenome in *G. hirsutum* (5.85 average residues) and the D-genome in *G. raimondii* (t-test, *p* < 0.05) (Additional file 2).

The complete lists of the DNA binding activity and bHLH domain categories of these proteins are provided in Additional file 2. The total number of DNA binding activities in each category in these two diploids is also approximately equal to those in *G. hirsutum* (Table 1). For example, the percentage of proteins in the major category, G-box binding, in *G. hirsutum* (~ 59.7%) is similar to that in *G. arboreum* (~ 59.1%) and in *G. raimondii* (~ 58.9%), also in other subcategories. Meanwhile, phylogenetic analysis showed that proteins grouped within the same subfamily tended to be composed of the same DNA-binding recognition motifs, further supporting the DNA-binding definition (Additional file 5). This result also provides additional support to the clustering found in the phylogenetic relationship.

The intron patterns, including intron distribution, positions, and phases, in genomic regions encoding the bHLH domains were also analyzed to investigate the conservation of *bHLH* genes between diploids and allotetraploid. Within the 871 cotton *bHLH* genes, 17 different intron distribution patterns (designated from A to Q) were identified, ranging from zero to four introns within the domain (Additional file 8). All the intron patterns, except patterns M and P, were consistent with those found in *Arabidopsis bHLH* genes [30]. The total numbers of proteins in most patterns in the two diploids corresponded to those in the allotetraploid, but in patterns B, C and E, significant variation occurred during allotetraploid formation (Additional file 3). In most cases, the intron position was conserved, even though the number of each pattern varied in these three cotton species. Furthermore, all of the introns with conserved positions also had identical phases, mainly phase 0, with respect to codons. In addition, intron pattern distribution was almost absolutely conserved within most subfamilies, providing another evidence for testing the reliability of our phylogenetic analysis (Additional file 8).

### Putative function and expression of homoeologous *bHLH* genes in allotetraploid cotton

Until now, only a few *bHLH* genes have been functionally characterized in plant species. One previous study showed that orthologous genes usually share similar functions and are clustered in the same clades and sub-clades, whereas homoeologous genes generally display different functions [30]. Using cotton, *Arabidopsis*, rice, cacao and grape bHLH proteins, a NJ phylogenetic tree that included 25 subfamilies was constructed (Additional files 6 and 9) to presume the functions of the *bHLH* genes corresponding to Arabidopsis bHLH family described by Pires and Dolan [34]. Maximum Parsimony and Maximum likelihood trees were generated to support the existence of most of these subfamilies (Additional files 10 and 11). Of the 28 bHLH subfamilies, eight lacked any functional characteristics.

**Fig. 1:**
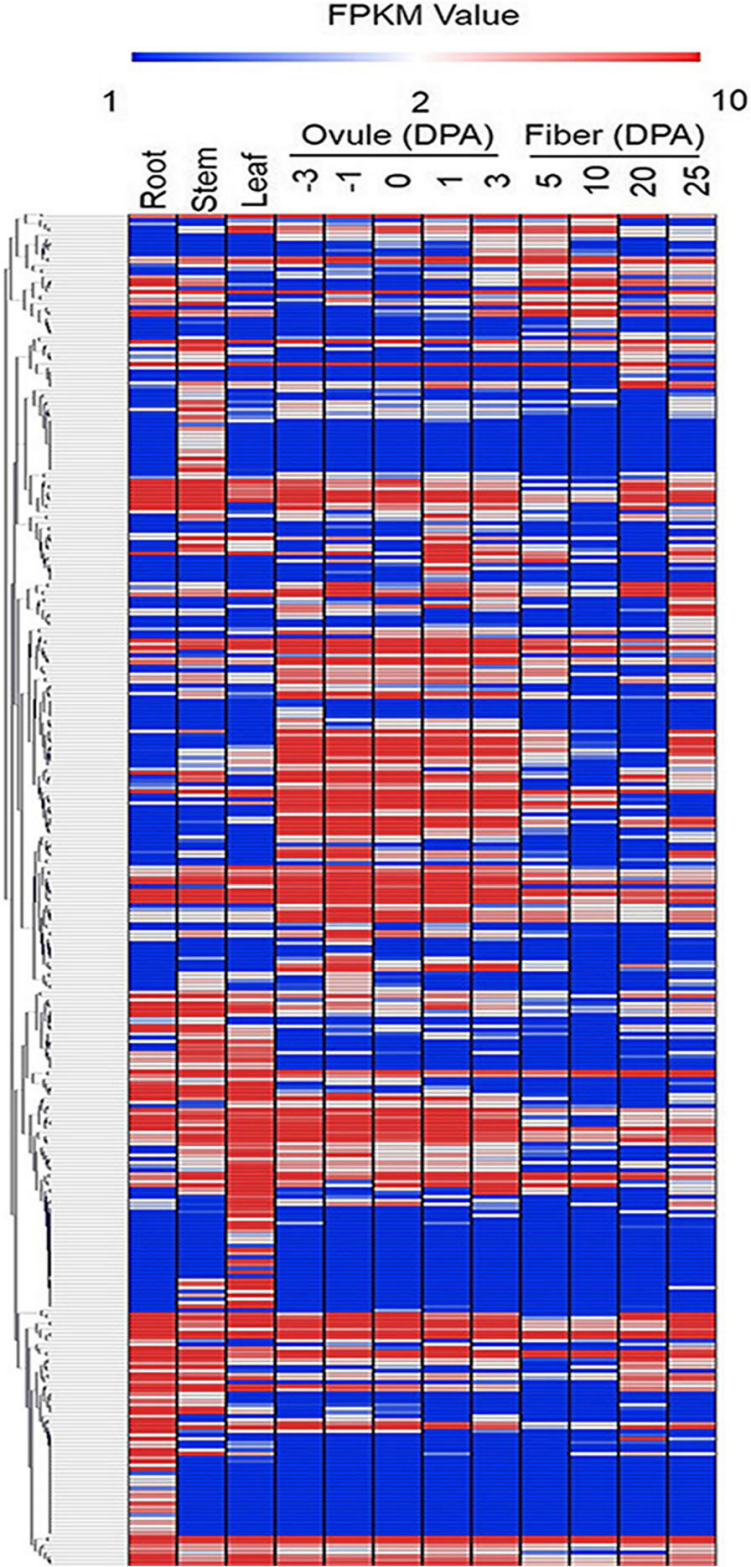
Expression patterns of *GhbHLH* genes from transcriptome sequencing. Expression clusters were produced by Mev4.6.2. 358 *bHLH* genes were tested in roots (R), stems (S), leaves (L), − 3-3 DPA ovules and 5–25 DPA fibers. DPA: day post-anthesis

Transcriptome analysis of datasets from different tissues (root, stem, leaf, ovules, fibers) in *G. hirsutum* revealed that of 432 *bHLH* genes, 74(17.1%)had no expression information, suggesting that they were pseudogenes or were expressed only at specific developmental stages or under special conditions. The remaining 358 *GhbHLH* genes (82.9%), which were expressed in roots, stems, leaves, ovules and fibers (Fig. 1), were clustered into 27 expression patterns by Mev4.6.2 (threshold≥0.5). Most *bHLH* genes were dominantly expressed in different tissues; only a few genes were constitutively expressed in all organs and developmental stages. In the allotetraploid, approximately one third (137, 31.7%) of the analyzed *bHLHs* were constitutively expressed in all of the four tissues tested, suggesting an extensive roles in cotton development. Of 432 *GhbHLHs*, 64 (14.8%) showed dominant expression in root, stem and leaf tissue but were not detected in fibers, while 63 (14.6%) were expressed only in fibers.

Although the expression pattern of *bHLHs* varied, we also detected conserved expression profiles. In addition to groups of genes that exhibited similar transcript abundance profiles but were relatively phylogenetically distinct, several phylogenetic clades shared, to a large extent, the same transcript abundance profile. Gene expression patterns can provide important clues for gene function. We subsequently compared these expression profiles against the phylogenetic tree. As shown in Additional file 12 c, some genes (homoeologous or orthologous) in a subfamily shared similar expression patterns and even, indiscriminate expression, indicating possible conserved and redundant functions. But not all of the genes had the similar character in Additional file 12 c. In contrast, the remaining genes exhibited significant divergence in their expression. For example, in subfamily 12, genes showed four expression patterns, mainly patterns 17 and 23, but all genes were highly expressed during fiber initiation and early elongation, corresponding to the trichome formation predicted in Additional file 9. In addition, a similar phenomenon was detected in subfamily 11, which was presumed to be involved in secondary metabolism. In this subfamily, genes were mainly assigned to patterns 19, 25, and 27, and were highly expressed in roots, which indicated they might participate in root development.

**Fig. 2:**
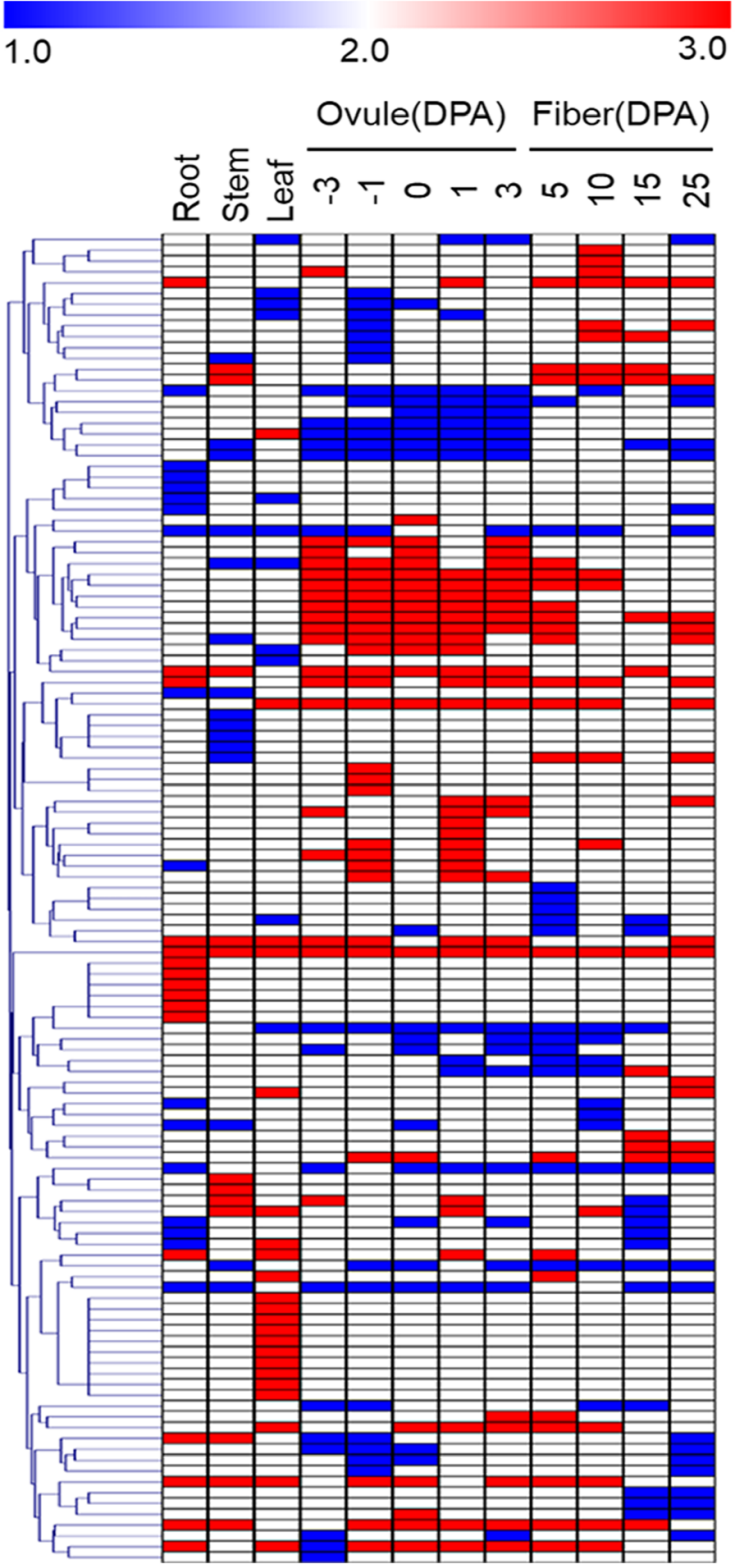
Differences in the expression of A- and D- homoeologous genes in *G. hirsutum*. Only 123 pairs of bHLH genes are presented here (significance was set at *P* < 0.01, FDR < 0.05 and at least two fold differences in average expression levels). 1, At > Dt indicated biased expression of the A homoeologs; 2, At = Dt indicated similar expression of the At and Dt homoeologs; 3, Dt > At indicated biased expression of the D homoeologs

To understand the temporal and spatial expression differences between the A-subgenome and the D-subgenome homoeologous genes in *G. hirsutum* (Fig. 2), the *GhbHLH* gene expression was also clustered by MEV4.6.2. It revealed that 156 pairs of the A- and D- subgenome homoeologous genes (87.3%, 165/189) had similar expression profiles (*r* ≥ 0.575; t-test, *P* ≤ 0.05) (Additional file 4). Of them, 67 have no difference in the subgenomic expression (35.4%, 67/189) in allotetraploid cotton (Additional file 12b), but 122 (64.6%, 122/189) pairs of *bHLH* genes were biased to A- (28.0%, 53/189) or the D-subgenome (36.5%, 69/189) (Fig. 2 and Additional file 12b), higher than the genome wide homeologous expression bias (40%) [17]. Biased expression of bHLH genes between A- and D-subgenome suggested their subfunctionalization, though 52.9% (100/189) of the pairs had a high Pearson correlation coefficient (*r* ≥ 0.575; t-test, *P* ≤ 0.05, Additional file 4). We found slightly more genes with expression bias toward the D-homoeologs than the A-homoeologs, which is consistent with the published data [17].

### Duplicated pair detection and divergence of transcription profiles in allotetraploid subgenomes

**Fig. 3:**
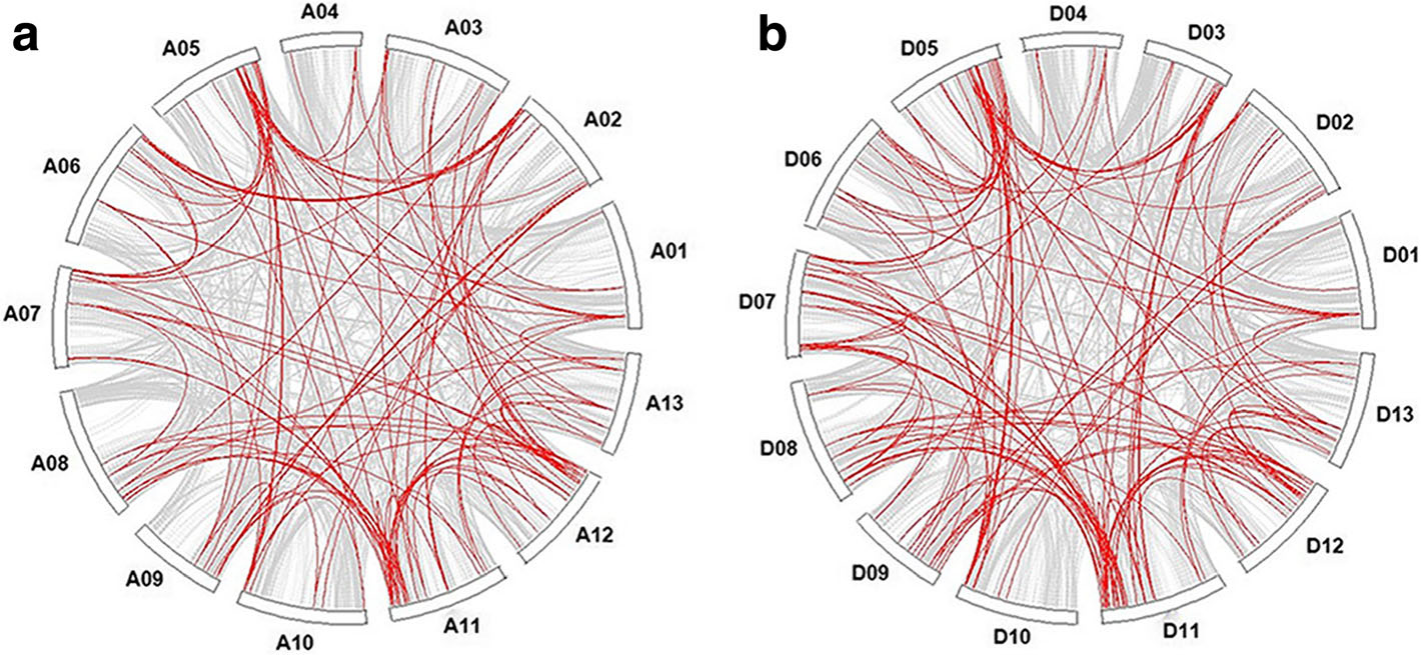
Distributions of duplicated pairs in the allotetraploid TM-1 genome. **a** and **b** show the duplicated pairs identified in the A- and D-subgenomes, respectively. Red and gray colored lines depict *bHLH* and whole genome duplicated pairs, respectively

**Fig. 4:**
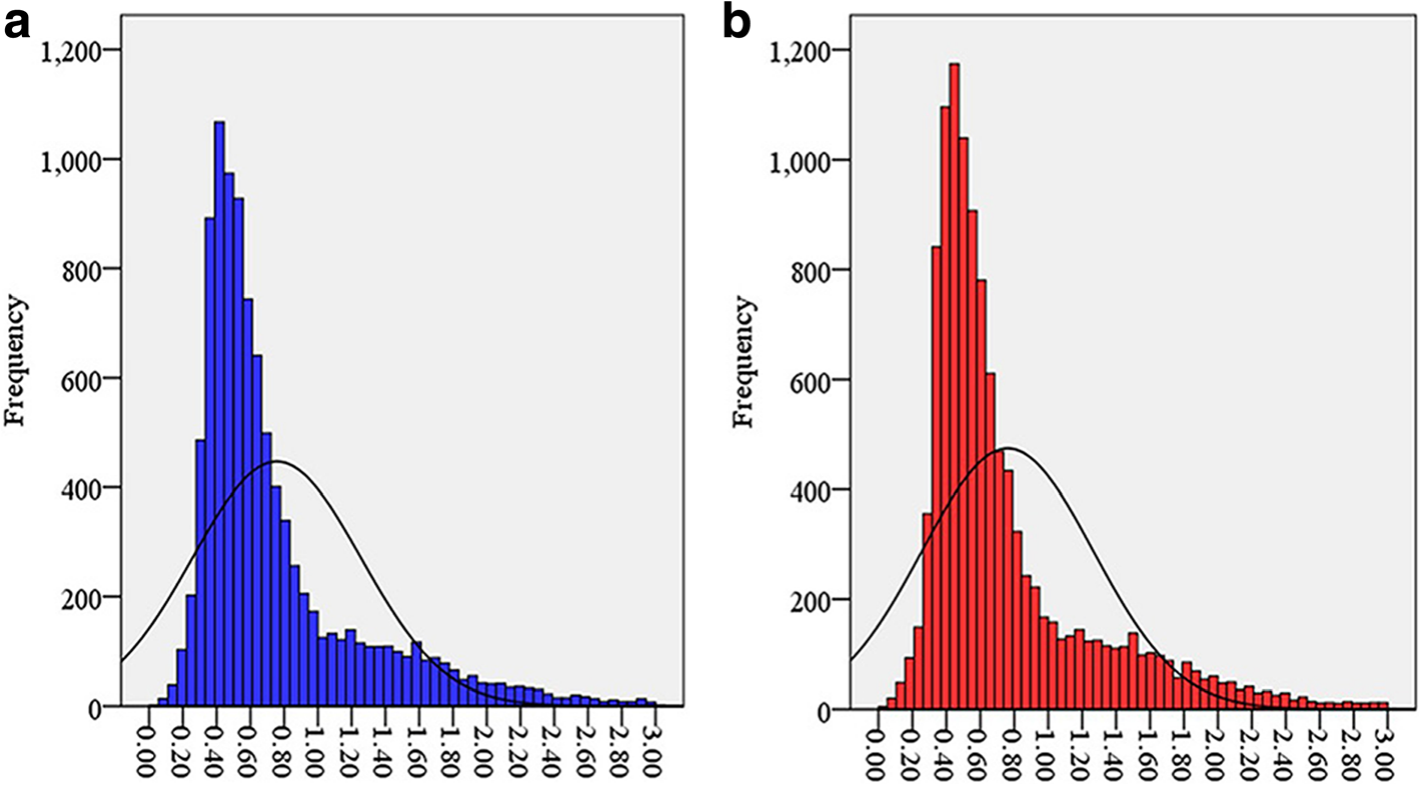
Frequency distributions of duplicated pairs in the A- and D-subgenomes based on Ks values. The Ks value of duplicated pairs in the A- and D-subgenomes have similar frequency distributions, and are mainly distributed in Ks = 0.3–0.9. **a**, the Ks value frequency distribution in A-subgenome; **b**, the Ks value frequency distribution in D-subgenome

Using MCSCAN (http://chibba.agtec.uga.edu/duplication/mcscan/), we detected 10,393 and 11,781 pairs of duplicated genes in the A- and the D-subgenome chromosomes, respectively based on the whole genome of *G. hirsutum* (Fig. 3). To assess the relative chronology of duplication events, the level of synonymous substitutions (Ks) between duplicated genes was estimated. For each pair of sister regions, we obtained the distribution of Ks values estimated from each pair of duplicated genes, excluding all values of Ks > 3.0 [13], because those sequences are highly saturated at synonymous sites and therefore uninformative. As all of the genes forming a duplicated block were almost certainly duplicated simultaneously (regardless of the actual duplication mechanism), their Ks values can be regarded as an independent random sample derived from genes duplicated at the same time. The results showed that the duplicated blocks of the A- and the D-subgenomes chromosomes fell into one major age group that ranged from 0.3 to 0.9, as indicated by the Ks levels (Fig. 4). This Ks value most likely represents a very old large-scale duplication event corresponding to the time of the five to six fold ploidy divergence from *T. cacao* (60 MYA, Ks = 0.312) and a prior paleopolyploidy event (100 MYA, Ks = 0.520) [33]. After the divergence from *T. cacao,* the duplication event was defined as “recent”, and mainly led to A- and D-progenitor divergence (6.0–6.3 MYA, Ks = 0.031–0.033) and allotetraploid reunion (1–1.5 MYA, Ks = 0.005–0.008) [35].

Based on the overview of duplication events in the *G. hirsutum* genome, four recent duplicated gene pairs (4.3%) and 89 old duplicated gene pairs belonging to the *bHLH* gene family (95.7%) were detected in the A-subgenome, and two (1.6%) recent and 120 old pairs (98.4%) were found in the D-subgenome (Additional file 13). This result also indicated that an old duplication event had the greater number of *bHLH* genes in the D- subgenome than that in the A-subgenome.

**Fig. 5:**
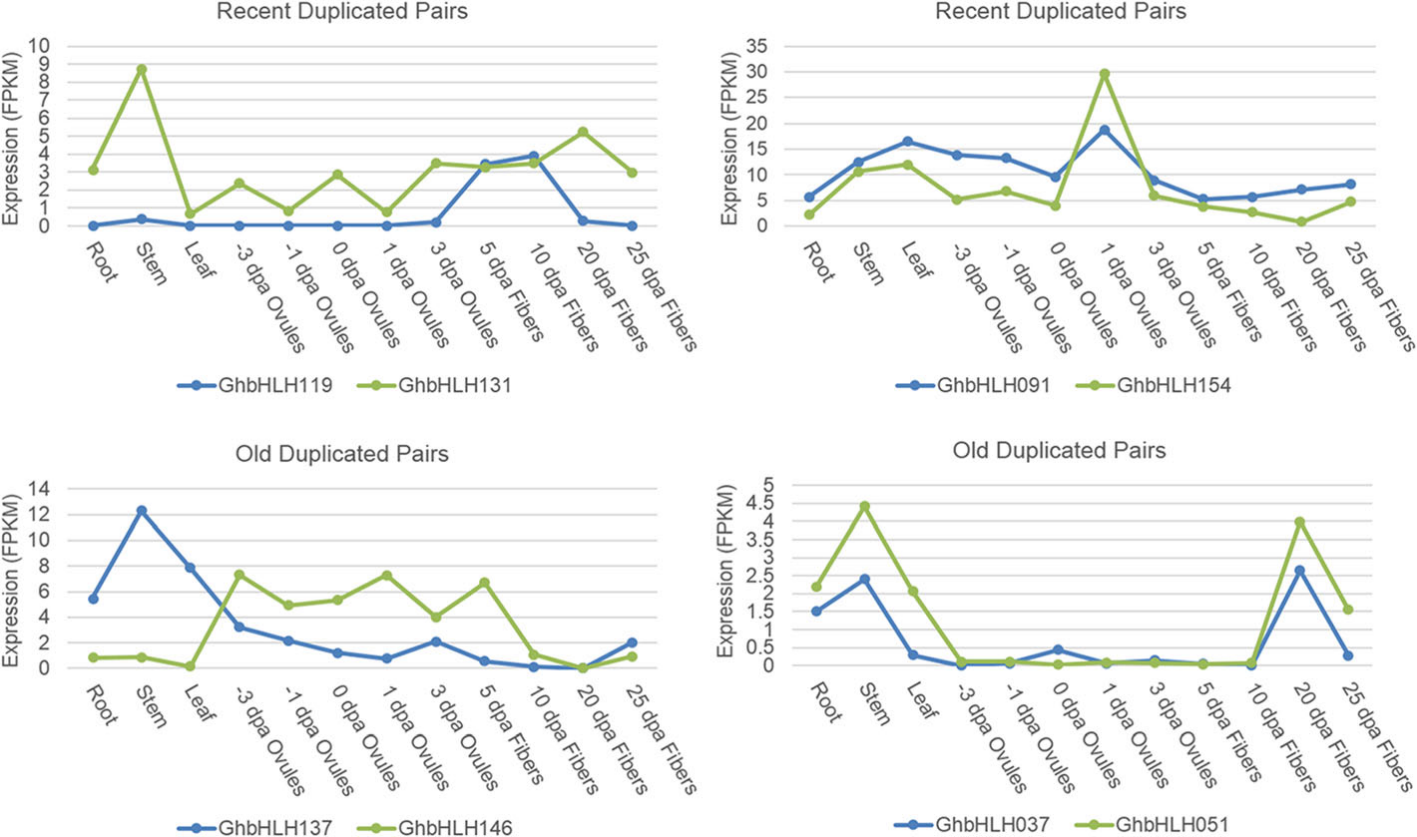
Representative examples of the expression profiles of paralogous pairs. Four ancient paralog pairs exhibited agreed or divergent expression profiles in the five tissues examined

The presumed functions of the *bHLH* genes are described in Additional file 9. Due to the relatively fast evolution and high sequence similarity, no divergent functions were found in the A- or the D-subgenome duplicated pairs. As the functions of some duplicated *bHLH* genes could not be distinguished by their sequence features or presumed functions, we obtained *bHLH* expression data from Texas Marker-1 (TM-1) analyses of various tissues [17]. The duplicated pairs formed by the recent polyploidy event and the old polyploidy events were analyzed separately. To determine a Pearson correlation coefficient (r value) below which duplicated gene pairs can be considered divergent, we calculated r between the expression profiles of all duplicated genes (13093) (Additional file 13). Since most of the duplicated pairs are not functionally related and not convergent, the distribution of r can be used to test the null hypothesis that the duplicated genes are convergent [36]. The r values obtained from all duplicated pairs were ≥0.575 (t-test, *P* ≤ 0.05) (Additional file 13), and this was then used as a criterion for determining that two duplicated genes have diverged in their expression (Fig. 5). This criterion was also used in a similar analysis of duplicated pairs in the D-subgenome. Expression profiles were available for 4 and 89 pairs of young and old duplicates, respectively, in the A-subgenome chromosomes, and 2 and 120 pairs of young and old duplicates, respectively, in the D-subgenome chromosomes (Table 2). Based on this condition (*r* < 0.575), we found that 50% (2/4) of the pairs of recent duplicates and 77.5% (69/89) of the pairs of old duplicates in the A-subgenome have diverged in their expression (Table 2). For 28.1% (25/89) of the old pairs, the correlation coefficient was negative (*r* < 0). In general, the results in the D-subgenome were similar to those in the A-subgenome, altogether 68.8% (148/215) in *G. hirsutum* genome (Table 2). These data provide further evidence of the conserved evolution of the *bHLH* genes in the A- and D-subgenomes.

**Table 2:** Statistics of recent and old bHLH duplicated pairs and expression Pearson correlation coefficient in allotetraploid

	Recent Duplicated Pairs	Divergence expression in recent duplicated pairs	Old Duplicated Pairs	Divergence expression in old duplicated pairs	Expression Pearson correlation coefficient (r)
A_subgenome	4	2	89	69	0.575
D_subgenome	2	1	120	76	0.575
Total	6	3	209	145	–

**Fig. 6:**
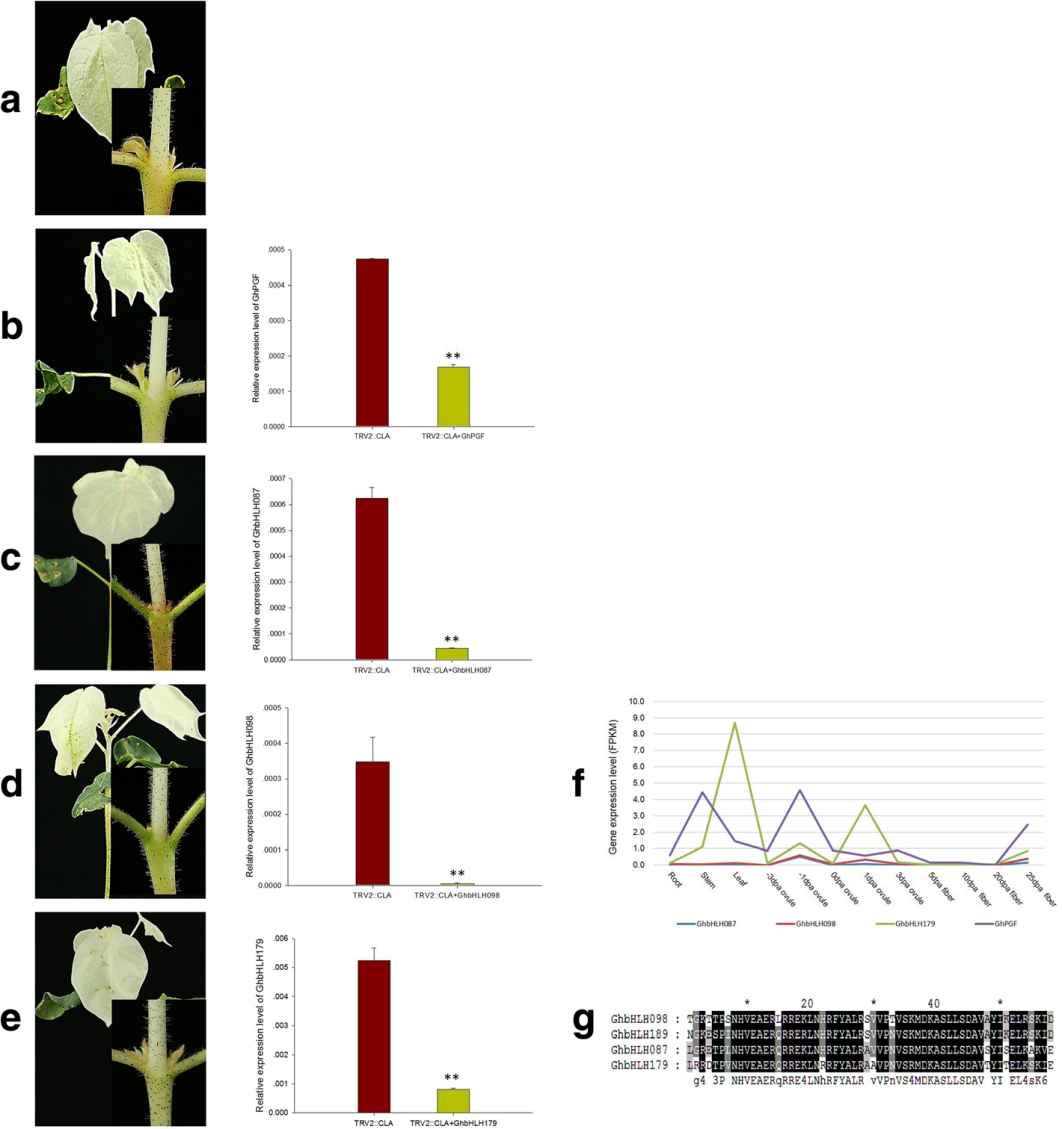
Functional characterization of *GoPGF* and its paralogous genes by VIGS. **a**-**e** Presence and absence of glands before and after genes were silenced by VIGS, showing the corresponding transcription levels of the genes in silenced leaves. **f-g** Difference of expression and aminao acids among *GoPGF* and its paralogous genes. A was the control, where the chloroplasts alterados 1 gene (CLA) was silenced. Bars represent the SD of three biological replicates and ** represents a *p*-value≤0.01 (t-test)

In order to evaluate our research on duplicated genes, we tested the functional divergence of a glandular trichome formation gene, *GhbHLH*189_At (*GoPGF*, GenBank accession number: KP072743), with its paralog genes (*GhbHLH*087, 098, 179) in the A-subgenome by virus induced gene silencing (VIGS) [20]. Q-PCR showed that all four genes had a low expression level after VIGS (Fig. 6). Silencing *GoPGF*_At led to a completely glandless phenotype, but no changes were detected in its duplicated genes (Fig. 6). These results suggest that *GoPGF*_At performs a function that has diverged from that of its duplicated genes.

## Discussion

### Evolutionary conservation and divergence of *bHLH* genes from diploids to Allotetraploid

Recent completion of whole genome sequences of cotton allowed us to perform a genome-wide analysis of the cotton *bHLH* genes and a comparison with other plant *bHLH* genes. Comparison of the *G. hirsutum* genome [17] with the already sequenced diploid *G. arboreum* (AA) (215) and *G. raimondii* (DD) (224) genomes revealed that *bHLH* genes were shared at orthologous positions, and had a conserved chromosomal distribution and gene numbers. Amino acid sequence comparison showed a relatively stable status between orthologous genes from the ancestor and *G. raimondii*, *G. arboreum* and *G. hirsutum* in terms of DNA binding activity, intron position and phylogenetic analysis. The same phenomenon was also found between the A- and the D-subgenomes. But, from the values obtained in the bootstrap analysis, it was apparent that the deep nodes of the tree have low statistical support (Additional file 5). This observation is mainly a result of the small size of the bHLH motif and the existence of numerous ancient paralogs [21]. As the internal nodes are not highly supported by phylogenetic statistics, we could not infer evolutionary relationships between the different subfamilies. But, within each subfamily, strong amino acid sequence conservation is evident from the short branch lengths at the tips of the tree, suggesting strong evolutionary relationships among subfamily members. Orthologous genes from *T. cacao* were similar in *G. arboreum* and *G. raimondii*, and in the A- and D-subgenomes. These results indicated a conserved evolution when two diploids were reunited into allotetraploid. Transcription of homoeologous pairs between the A- and D-subgenomes may even lead us to understand the true proportion of conserved pairs or divergent pairs in the allotetraploid for several reasons; for example, most homoeologous genes that evolved from the same ancestor had largely similar profiles with no difference or bias. However, some pairs had unequal expression in a small subset of conditions or tissues, although there was a significant positive correlation coefficient. This resulted in the dominant expression of Gh_At or Gh_Dt *bHLH* genes, which would also mask a genuine divergence in their expression patterns or functions, and could be a result of adaptability inherited from its progenitors.

Some changes in the number of loss genes also occurred in the evolution from diploid to allotetraploid. For example, *bHLH* gene loss is rare in most species; however here two genes were lost in the A-subgenome and two in the D-subgenome. The expression of *bHLH*s in cotton supports the conservation of function in orthologous genes across plant species. Discrepancies in the expression patterns of genes in the same subfamily, as found in the phylogenetic tree, may indicate a divergence in function. Although the functions of most *bHLHs* are unknown, our phylogenetic and expression analyses provide a solid foundation for future functional studies in cotton and other species.

### Old duplication might played a key role in expansion of bHLH family

Duplication of single genes, chromosomes and whole genomes are major forces in the evolution of plant genome structure and content. The increasing number of sequenced genomes has provided the opportunity to investigate ancient polyploidy and duplication events [15, 37]. In this study, the number of orthologous genes from cacao were nearly equal in two diploids (39.5%, 40.2%) and A−/D-subgenomes (42.6%, 42.08%), and these only account for part of the bHLH superfamily, reflecting a number of different evolutionary processes. The loss or retention of duplicated genes is a nonrandom process. Duplicated genes are referred to as “old” and “recent” based on the Ks-value. In our study, an old duplication event was found to have made a major contribution to the expansion of the *bHLH* number, corresponding to a WGD. Tandem gene duplication was also deemed to play an important role in the gene expansion [38], but only two tandem duplication pairs in the two diploids and four pairs in the allotetraploid were identified. These results also indicate that few recent duplication events occurred after the WGD, and this idea is supported by the Ks value. Additionally, the intron position patterns in the bHLH domain provide evidence of a common origin of closely related bHLHs. As shown in Additional files 2 and 3, the genes that were related by the presumed number of duplications also shared conserved intron position patterns and DNA binding activities.

### Functional divergence of duplicated genes

Here, we present an analysis of the long term expression divergence of duplicated genes in cotton, evolving from an ancient ancestors [17]. Assuming that *r* < 0.575 between expression profiles is an indication of expression divergence, the most striking result is that most of the duplicate pairs (Additional file 13) have acquired divergent expression patterns. This observation is in agreement with previous analyses in *Arabidopsis* and cotton, which show a rapid divergence in expression between duplicated genes [36]. For example, *GoPGF*, which encodes a basic helix-loop-helix (bHLH) domain-containing transcription factor, was reported to regulate glandular cell formation and gossypol content [20]. Interestingly, *GoPGF* and *AtMYC2* were clustered in the same subfamily in the present study (Additional file 6), but *AtMYC2* performed a completely different function; it was found to regulate sesquiterpene synthase gene expression and modulates diverse JA-mediated resistance to insect pests in *Arabidopsis* [39, 40]*.* Another gene, *AtGL3*, regulates non-glandular trichome development, though its closest homologous relationships were with *GoPGF* and *AtMYC2* [41–43]. This suggests a different function after divergence from the ancestor. Recent duplication suggests that expression divergence did not occur and these genes experienced a slow evolution after divergence from *Theobroma cacao.* Collectively, our data suggest that expression divergence of ancient duplicated genes is a common phenomenon, and may be evident in different developmental stages and different tissues. Moreover, our results indicate that the fraction of transcriptionally divergent pairs is greater in the set of ancient paralogous genes than in the set of recent paralogous genes. We also observed duplicated pairs with similar profiles that were expressed differently in small subsets of tissues, though the correlation coefficients of these were still significantly positive.

The duplicated genes have a high sequence similarity, suggesting that their function cannot be discriminated by a phylogenetic tree. Genes that remain duplicated, such as *GhPGF* and its duplicated genes (Fig. 6), do not tend to retain redundant functions: their divergent expression patterns indicate that neo-functionalization may have occurred in the long term evolution. The Pearson correlation coefficient showed that *GhPGF* coevolved with *GhbHLH087* and *GhbHLH098*, but not *GhbHLH179*; however, our results suggest that *GhPGF* functionally diverged from its duplicated genes.

## Conclusion

Here, we showed an overview of conservation in two diploids and also lasted in allotetraploid. Our studies show that the cotton *bHLH* gene family has likely expanded considerably due to potentially old duplications during the evolution, allowing both the conservation and divergence of gene functions. Combining the data on inferred gene duplications with clustering analysis of genes provides a framework for visualizing the likely functional diversity within groups of related genes such as those encoding bHLH proteins. Genome-wide studies of expression divergence between *bHLH* genes and presumed functional data indicating that *bHLH* genes in cotton perform a variety of functions in different tissues and multiple fiber developmental stages. Our results provided an excellent example to clarify the origin and diversification of other dynamic and fascinating gene family for further studies and the growing genomic data from across plants will refine our knowledge of ancestor and descendant genomes.

## Methods

### Identification of bHLH proteins in cotton and other species

Using a consensus sequence based on that predicted by Atchley and Fitch [44] for the bHLH motif, cotton genomic peptide sequences were scanned using the PBLAST algorithm. To identify a large number of candidate *bHLH* sequences in the *G. arboretum, G. raimondii and G. hirsutum* databases, we used the bHLH domain (60 amino acids) encoded by a putative cotton *bHLH* gene *GhDEL61* (Gene-Bank number AAK19612) as a multiple BLAST query. We identified unique hits and removed redundant sequences from candidate *bHLH* genes according to their corresponding sequences and chromosome locations. All sequences were investigated to predict the bHLH domain using a Hidden Markov Model (HMMER) search and bHLH consensus motif development, as described by Atchley et al. [45]. PF00010_seed.txt from PFAM web site (http://pfam.xfam.org/) was used to identify candidate bHLH proteins with HMMER3.0. Search Parameters are as follows: (1) Sequence database: uniprotrefprot; (2) Significance threshold (E-value): 0.01 for sequence matches, 0.03 for hit matches; (3) Reporting threshold (E-value): 1 for both sequences and hits; (4) Filter: Bias composition filtering on. The number designation of the *bHLH* genes was based on their order on the chromosome. Names were composed of ‘bHLH’ and a number, and links between sequence names and the gene and protein identifiers from the corresponding genome are shown in Additional file 1. Previously, some genes that did not encode a bHLH motif according to our definition (lacking several amino acids that are highly conserved) had been classified as *bHLH* genes. These were excluded from our analysis.

### Multiple sequence alignments and intron/exon structure analysis

The amino acids of bHLH region were extensively aligned in BioEdit (http://www.mbio.ncsu.edu/BioEdit/BioEdit.html). The alignment was then adjusted manually according to the location of the corresponding amino acids in the bHLH motif, and similar amino acids were highlighted using BioEdit. The MEME version 3.5.7 tool was used to identify conserved motifs that were shared among bHLH proteins [46]. The following parameter settings were used: maximum number of different motifs to find, 50; optimum motif width, 8 to 100. Subsequently, the MAST program was used to search for the detected motifs in protein databases [47].

To obtain information on the intron/exon structure, the cDNA alignment of the bHLH domain sequences was obtained from the amino acid sequence alignment, and the intron distribution pattern and intron splicing phases were derived from the aligned cDNA sequences.

### Phylogenetic analysis

Reconstruction of evolutionary relationships was performed using the amino acid sequences of the bHLH proteins. Only the bHLH domain was used, because the flanking sequences of the bHLH proteins from independent subfamilies were either nonhomologous or too divergent to be reliably alignedPhylogenetic analysis was carried out with bHLH sequences from different species, using different methods [24, 34, 48]. The Jones, Taylor, and Thorton (JTT) model with an estimated proportion of the invariable sites (I) and an estimated g-distribution parameter (G) was selected as the best-fitting amino acid substitution model. NJ and MP analyses were carried out using MEGA 5.0 [49]. In the NJ analysis, distances were calculated using the JTT amino acid substitution model. To account for short insertions/deletions (commonly occurring throughout the loop region), “pairwise deletion” and “all sites” settings were used in NJ and MP analyses, respectively. A bootstrap analysis with 1000 replicates was performed in each case. Different subfamilies of phylogenetic tree are identified with a predictive value. The numbers besides the branched represent bootstrap support values (> 50%) from 1000 replications.

### Correlation coefficient and expression bias of homoeolog pairs

Genes were presumed to be expressed if FPKM> 1 (fragments per kilobase of transcript per million reads sequenced). If the correlation coefficient of homoeolog pairs is *r* ≥ 0.575 (t-test, *P* ≤ 0.05), we consider they possess similar expression pattern. The criterion for similar expression pattern *r* ≥ 0.575 is based on whole genome homoeolog pairs, corresponding to t-test, *P* ≤ 0.05. To evaluate expression bias between homoeologous gene pairs in each tissue, homeolog-specific read counts for each gene pair were compared using Fisher’s exact test. Then differential expression between gene pairs was delimited by 1.5-fold expression changes and with an FDR < 0.05 [17]. At>Dt indicated biased expression of the A homoeologs (Ahigh) and Dt > At indicated biased expression of the D homoeologs (Dhigh).

### Assessing ortholog groups and segmental block detection

Orthologous genes were identified by Inparanoid_4.1 (http://software.sbc.su.se/cgi-bin/request.cgi?project=inparanoid) and the phylogenetic tree of *T. cacao*, *G. arboreum*, *G. raimondii* and *G. hirsutum*. Groups of orthologs were identified as follows: Score cutoff 40 bits; In-paralogs with confidence was more than 0.05; Sequence overlap cutoff 0.5; Group merging cutoff 0.5.

For the detection of large segmental duplications, we consulted the duplicated blocks map provided by MCScanX algorithm (http://chibba.agtec.uga.edu/duplication/mcscan/). In our analysis, a link was created between two similar genes if (1) alignment between the corresponding proteins gave an E-value lower than 1e-20, (2) the E-value did not exceed 1e20 times the E-value of the best non-self-hit in order to restrict the analysis to the closest family members, and (3) at least 50% of the longest sequence was aligned. (4) Finally, a minimum of 6 unduplicated genes were allowed in a block region. Synonymous substitutions (Ks) were calculated according to the default value in MCScanX.

### Identification of recent and old duplicated pairs

Duplicated *bHLH* pairs were extracted from the segmental duplication results. Pairs of duplicated regions that shared 6 or more duplicated genes (sm ≥ 6) in each pair of blocks were identified by MCSCAN (http://chibba.agtec.uga.edu/duplication/mcscan/). Synonymous substitutions (Ks) were used to distinguish the recent and old duplicated pairs based on the WGD event.

Expression data for *GhbHLH* genes were obtained from RNA-Seq [17]. These datasets correspond to expression intensities under various tissues. Expression patterns were clustered by Mev4.6.2 using the Hierarchical Clustering model (http://www.softpedia.com/get/Science-CAD/MeV.shtml). To analyze the global expression pattern of the two subgenomes, we compared the average FPKM values of homoeologs in four tissues. The expression profiles of two duplicated genes should be virtually similar (subfunctionalization) or discriminated (neofunctionalization) just after duplication, so the degree of similarity between the expression profiles of each pair of duplicated genes across various tissues was measured using the Pearson correlation coefficient (*r*) and their correlation coefficient is a criterion measure. The criterion for expression divergence *r* < 0.575 was obtained based on the correlation coefficient of whole genome duplicated pairs, corresponding to t-test, *P* > 0.05. Hence, if two duplicated genes do not show evidence of similarity (*r* ≤ 0.575) (i.e., has a low r value), they might have acquired divergent functions.

### Quantitative RT–PCR analysis

RNA was extracted from cotton leaves using a BioFlux kit. First-strand cDNA was generated using TransScript One-Step gDNA Removal and cDNA Synthesis SuperMix (TransGen Biotec Co., Ltd.) according to the manufacturer’s instructions. Q-PCR assays were performed in a 7500 Real-Time PCR System (Applied Biosystems) using First Start Universal SYBR Green Master (Roche). The cotton *Histone3* gene (AF024716) was used as aninternal control and the relative expression levels of the genes were calculated using the comparative threshold cycle method. The amplification efficiency of each gene was calculated. The Q-PCR cycles were as follows: (1) 95 °C, 10 min; (2) 40 cycles of 95 °C for 15 s, 60 °C for 30 s and 72 °C for 30 s; (3) a melting curve analysis from 65 to 95 °C (1 s hold per 0.2 °C increase) to check the specificity of the amplified product. The relative expression level of the *GhPGF* was calculated by the eq. *Y* = 2^-ΔCt (where ΔCt is the difference between the Ct values of the *His3* products and the *GhPGF* product; i.e. ΔCt = Ct*GhPGF*–Ct*His3*). Quantitative RT–PCR was performed with the primers listed in Additional file 14.

### Virus-induced gene silencing assay

To silence the expression of *GhPGF* and its duplicated genes, a fragment of cDNA of the genes was PCR-amplified using Pfu DNA polymerase (Promega) and primers (Additional file 14). The resulting PCR products were cloned into *EcoRI-BamHI-cut pTRV2* to produce a VIGS vector. The *pTRV1*- and *pTRV2*-gene vectors were introduced into *Agrobacterium* strain GV3101 by electroporation (Bio-Rad, Hercules, CA, USA). Details of the VIGS method used have been published by Dan Ma et al. (2016) [20]. The plants were grown in pots at 25 °C in a growth chamber under a 16-h light per 8-h dark cycle with 60% humidity.

## Additional files

Additional file 1: Summary of information on the bHLH proteins in cotton and other species. (XLSX 62 kb)

Additional file 2: Statistical analysis of amino acids in the basic motif and their DNA-binding activities. (XLSX 81 kb)

Additional file 3: Intron distribution within the bHLH domain of cotton bHLH proteins (XLSX 46 kb)

Additional file 4: Ortholog and homoeologous genes identified in cacao or cottons. (XLSX 51 kb)

Additional file 5: NJ phylogenetic tree of the cotton *bHLH* members. This tree shows the subfamilies, the predicted DNA-binding activities and the intron distribution pattern. (PDF 73 kb)

Additional file 6: NJ phylogenetic tree of the *bHLH* members in cotton and other species. (PDF 623 kb)

Additional file 7: Multiple sequence alignment of the bHLH domains of the 871 members of the cotton bHLH protein family. (PDF 392 kb)

Additional file 8: Intron distribution within the bHLH domains of three cotton species. (JPEG 122 kb)

Additional file 9: Functional characterization of bHLH proteins presumed from different plant species. (XLSX 18 kb)

Additional file 10: MP phylogenetic tree of the *bHLH* members in cotton and other species. (PDF 103 kb)

Additional file 11: ML phylogenetic tree of the *bHLH* members in cotton and other species. (PDF 102 kb)

Additional file 12: Tissues specific expression and bias expression of A_ and D_subgenome genes and comparison of expression profiles and the phylogenetic trees. (XLSX 2598 kb)

Additional file 13: Ka and Ks values of *bHLH* duplicated pairs and expression Pearson correlation coefficients (r). (XLSX 270 kb)

Additional file 14: Primers used in this study. (XLSX 9 kb)

## Abbreviations

*AtGL3*: Arabidopsis GLABRA3
bHLH: Basic/helix-loop-helix
EST: Expressed sequence tags
FPKM: Fragments per kilobase of transcript per million reads sequenced
Ga: *Gossypium arboreum*
Gh: *Gossypium hirsutum*
*GoPGF*: Pigment gland formation
Gr: *Gossypium raimondii*
Ks: Synonymous substitutions
MYA: Million years ago
qPCR: Quantitative polymerase chain reaction
Tc: *Theobroma cacao*
VIGS: Virus induced gene silencing
WGD: Whole-genome duplication
