# Supplementary material for: Divergence and evolution of cotton bHLH proteins from diploid to allotetraploid
Source: BMC Genomics. 2018 Feb 23;19:162. doi: 10.1186/s12864-018-4543-y (PMC5824590; doi:10.1186/s12864-018-4543-y)
Supplement: Supplementary file 5 — NJ phylogenetic tree of the cotton bHLH members. This tree shows the subfamilies, the predicted DNA-binding activities and the intron distribution pattern. (PDF 73 kb) [file 12864_2018_4543_MOESM5_ESM.pdf]

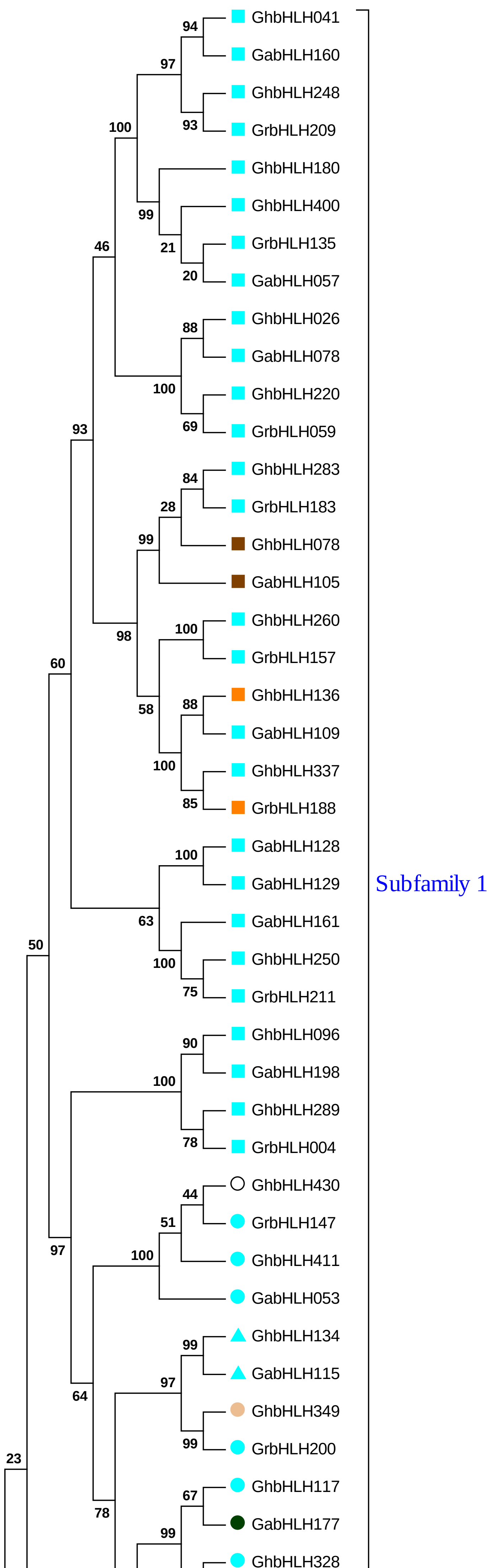
GhbHLH041
GabHLH160
GhbHLH248
GrbHLH209
GhbHLH180
GhbHLH400
GrbHLH135
GabHLH057
GhbHLH026
GabHLH078
GhbHLH220
GrbHLH059
GhbHLH283
GrbHLH183
GhbHLH078
GabHLH105
GhbHLH260
GrbHLH157
GhbHLH136
GabHLH109
GhbHLH337
GrbHLH188
GabHLH128
GabHLH129
GabHLH161
GhbHLH250
GrbHLH211
GhbHLH096
GabHLH198
GhbHLH289
GrbHLH004
GhbHLH430
GrbHLH147
GhbHLH411
GabHLH053
GhbHLH134
GabHLH115
GhbHLH349
GrbHLH200
GhbHLH117
GabHLH177
GhbHLH328
Subfamily 1

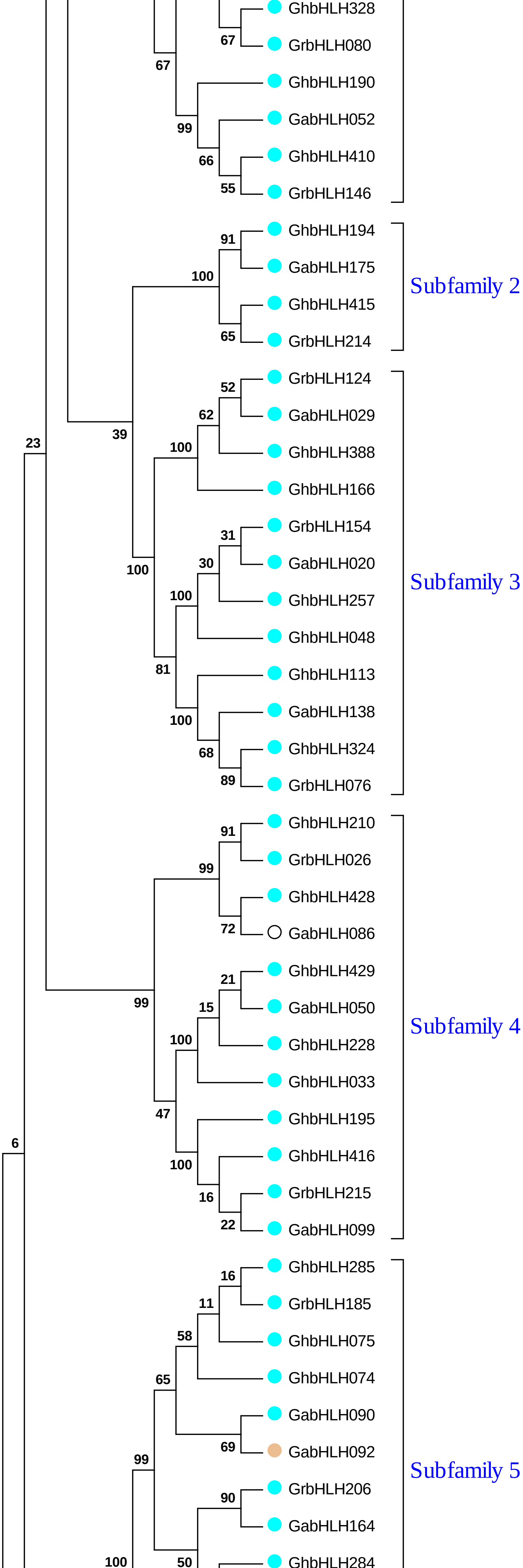

GhbHLH328
GrbHLH080
GhbHLH190
GabHLH052
GhbHLH410
GrbHLH146
GhbHLH194
GabHLH175
GhbHLH415
GrbHLH214
Subfamily 2
GrbHLH124
GabHLH029
GhbHLH388
GhbHLH166
GrbHLH154
GabHLH020
GhbHLH257
GhbHLH048
GhbHLH113
GabHLH138
GhbHLH324
GrbHLH076
Subfamily 3
GhbHLH210
GrbHLH026
GhbHLH428
GabHLH086
GhbHLH429
GabHLH050
GabHLH228
GhbHLH033
GhbHLH195
GhbHLH416
GrbHLH215
GabHLH099
Subfamily 4
GhbHLH285
GrbHLH185
GhbHLH075
GhbHLH074
GabHLH090
GabHLH092
GrbHLH206
GabHLH164
GhbHLH284
Subfamily 5

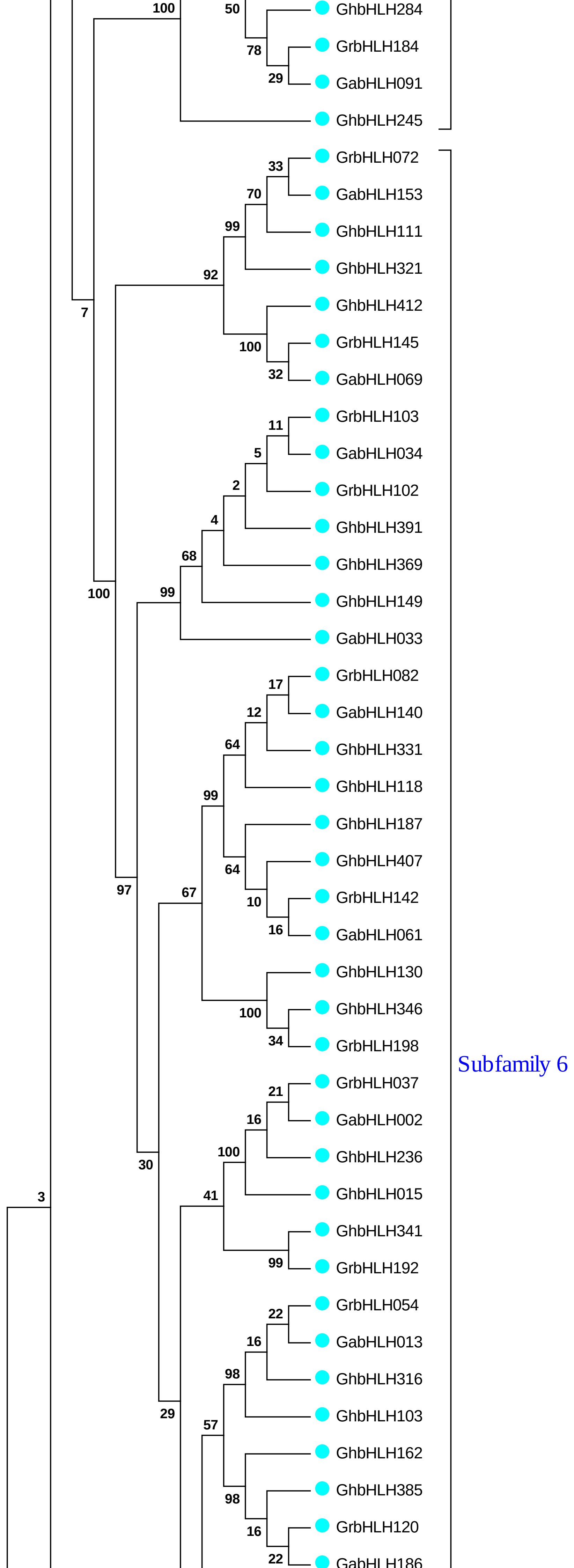
GhbHLH284
GrbHLH184
GabHLH091
GhbHLH245
GrbHLH072
GabHLH153
GhbHLH111
GhbHLH321
GhbHLH412
GrbHLH145
GabHLH069
GrbHLH103
GabHLH034
GrbHLH102
GhbHLH391
GhbHLH369
GhbHLH149
GabHLH033
GrbHLH082
GabHLH140
GhbHLH331
GhbHLH118
GhbHLH187
GhbHLH407
GrbHLH142
GabHLH061
GhbHLH130
GhbHLH346
GrbHLH198
Subfamily 6
GrbHLH037
GabHLH002
GhbHLH236
GhbHLH015
GhbHLH341
GrbHLH192
GrbHLH054
GabHLH013
GhbHLH316
GhbHLH103
GhbHLH162
GhbHLH385
GrbHLH120

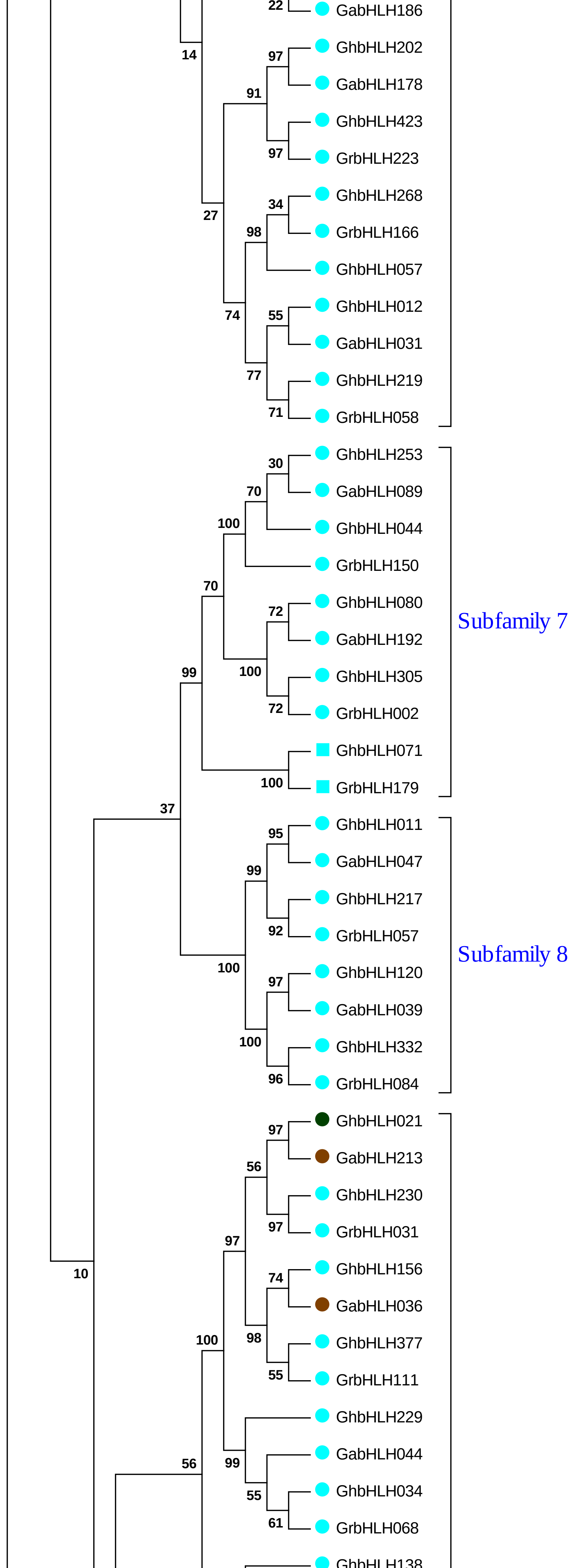
GabHLH186
GhbHLH202
GabHLH178
GhbHLH423
GrbHLH223
GhbHLH268
GrbHLH166
GhbHLH057
GhbHLH012
GabHLH031
GhbHLH219
GrbHLH058
GhbHLH253
GabHLH089
GhbHLH044
GrbHLH150
GhbHLH080
GabHLH192
GhbHLH305
GrbHLH002
GhbHLH071
GrbHLH179
Subfamily 7
GhbHLH011
GabHLH047
GhbHLH217
GrbHLH057
GhbHLH120
GabHLH039
GhbHLH332
GrbHLH084
Subfamily 8
GhbHLH021
GabHLH213
GhbHLH230
GrbHLH031
GhbHLH156
GabHLH036
GhbHLH377
GrbHLH111
GhbHLH229
GabHLH044
GhbHLH034
GrbHLH068

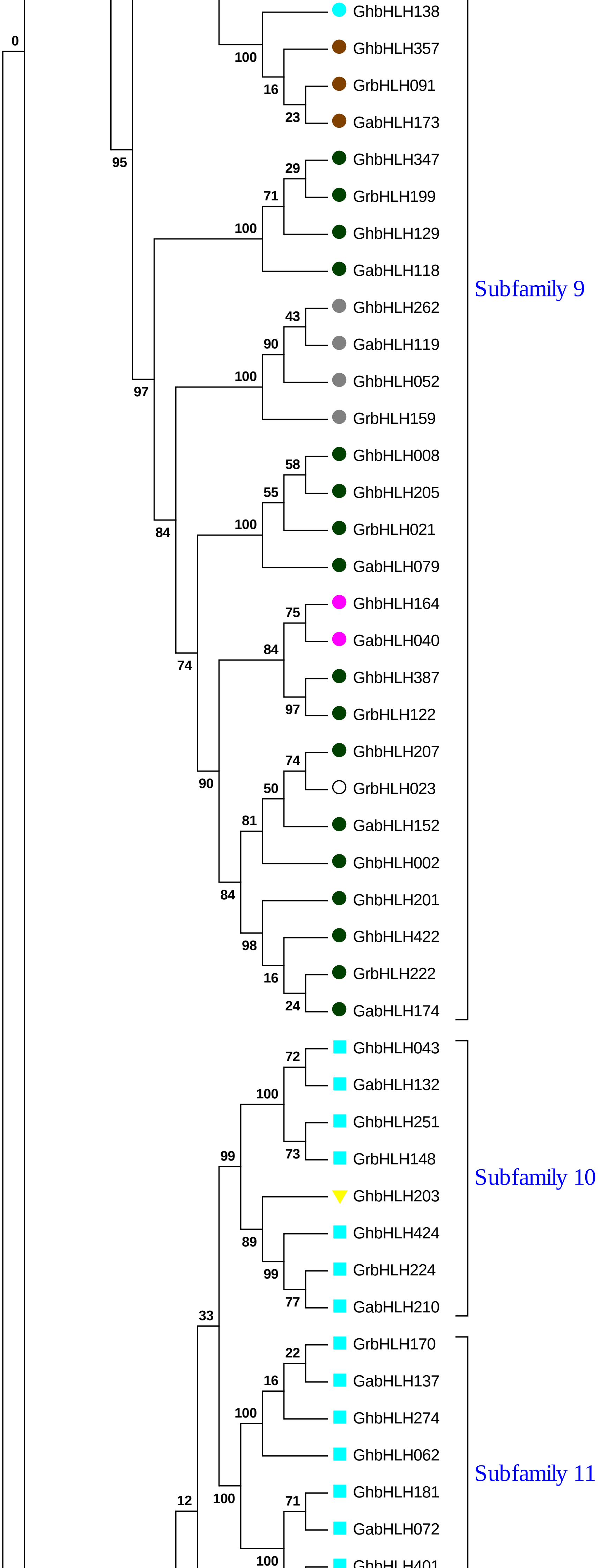
GhbHLH138
GhbHLH357
GrbHLH091
GabHLH173
GhbHLH347
GrbHLH199
GhbHLH129
GabHLH118
GhbHLH262
GabHLH119
GhbHLH052
GrbHLH159
GhbHLH008
GhbHLH205
GrbHLH021
GabHLH079
GhbHLH164
GabHLH040
GhbHLH387
GrbHLH122
GhbHLH207
GrbHLH023
GabHLH152
GhbHLH002
GhbHLH201
GhbHLH422
GrbHLH222
GabHLH174
Subfamily 9
GhbHLH043
GabHLH132
GhbHLH251
GrbHLH148
GhbHLH203
GhbHLH424
GrbHLH224
GabHLH210
Subfamily 10
GrbHLH170
GabHLH137
GhbHLH274
GhbHLH062
GhbHLH181
GabHLH072
Subfamily 11
0
95
100
16
23
29
71
100
97
43
90
100
84
58
55
100
74
75
84
97
90
74
50
81
84
98
16
24
72
100
73
99
89
99
77
33
22
16
100
12
100
71
100

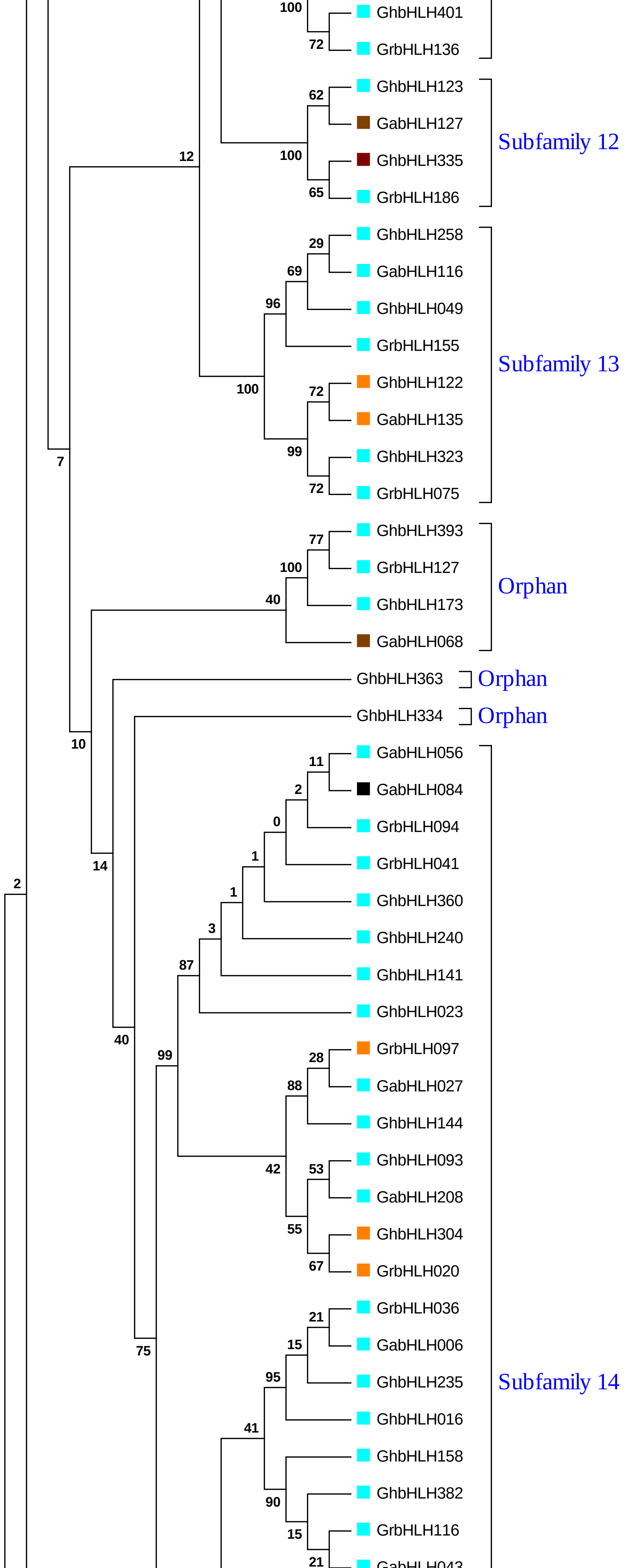
GhbHLH401
GrbHLH136
GhbHLH123
GabHLH127
GhbHLH335
GrbHLH186
Subfamily 12
GhbHLH258
GabHLH116
GhbHLH049
GrbHLH155
GhbHLH122
GabHLH135
GhbHLH323
GrbHLH075
Subfamily 13
GhbHLH393
GrbHLH127
GhbHLH173
GabHLH068
Orphan
GhbHLH363
Orphan
GhbHLH334
Orphan
GabHLH056
GabHLH084
GrbHLH094
GrbHLH041
GhbHLH360
GhbHLH240
GhbHLH141
GhbHLH023
GrbHLH097
GabHLH027
GhbHLH144
GhbHLH093
GabHLH208
GhbHLH304
GrbHLH020
GrbHLH036
GabHLH006
GhbHLH235
GhbHLH016
GhbHLH158
GhbHLH382
GrbHLH116
Subfamily 14

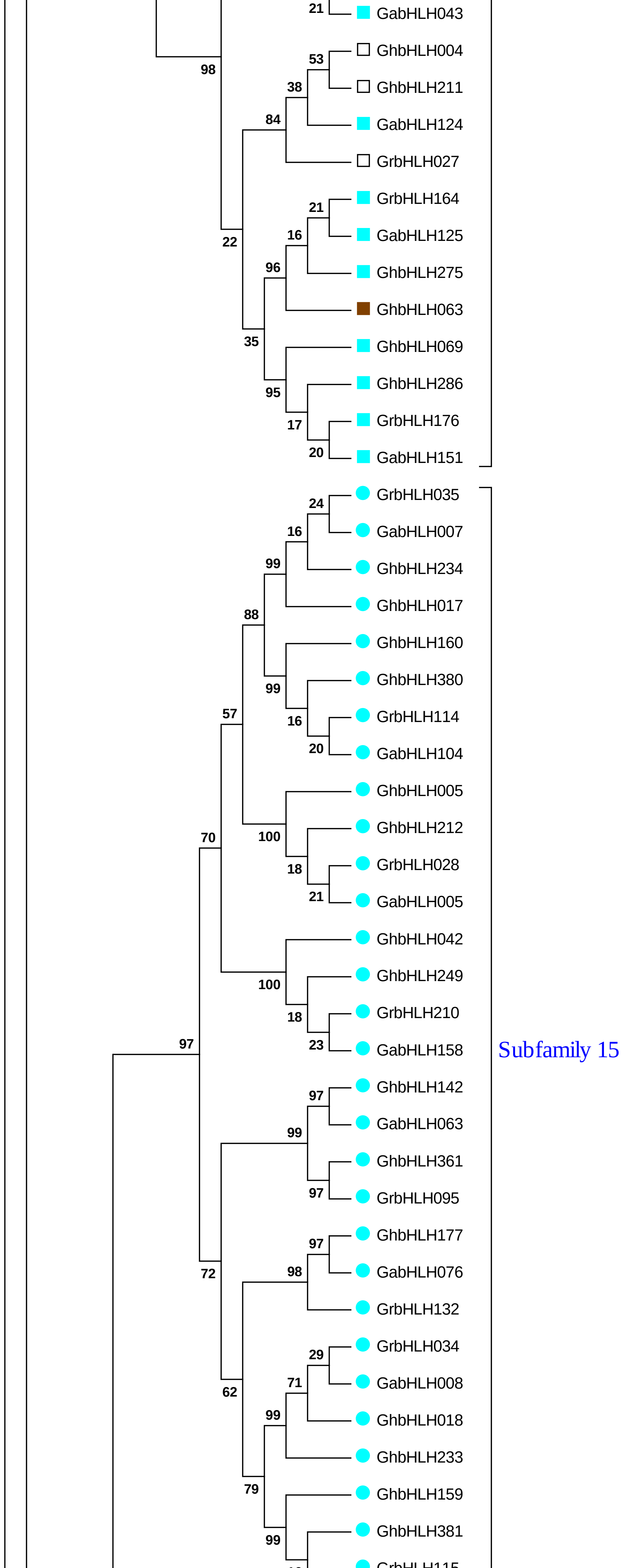
GabHLH043
GhbHLH004
GhbHLH211
GabHLH124
GrbHLH027
GrbHLH164
GabHLH125
GhbHLH275
GhbHLH063
GhbHLH069
GhbHLH286
GrbHLH176
GabHLH151
GrbHLH035
GabHLH007
GhbHLH234
GhbHLH017
GhbHLH160
GhbHLH380
GrbHLH114
GabHLH104
GhbHLH005
GhbHLH212
GrbHLH028
GabHLH005
GhbHLH042
GhbHLH249
GrbHLH210
GabHLH158
Subfamily 15
GhbHLH142
GabHLH063
GhbHLH361
GrbHLH095
GhbHLH177
GabHLH076
GrbHLH132
GrbHLH034
GabHLH008
GhbHLH018
GhbHLH233
GhbHLH159
GhbHLH381

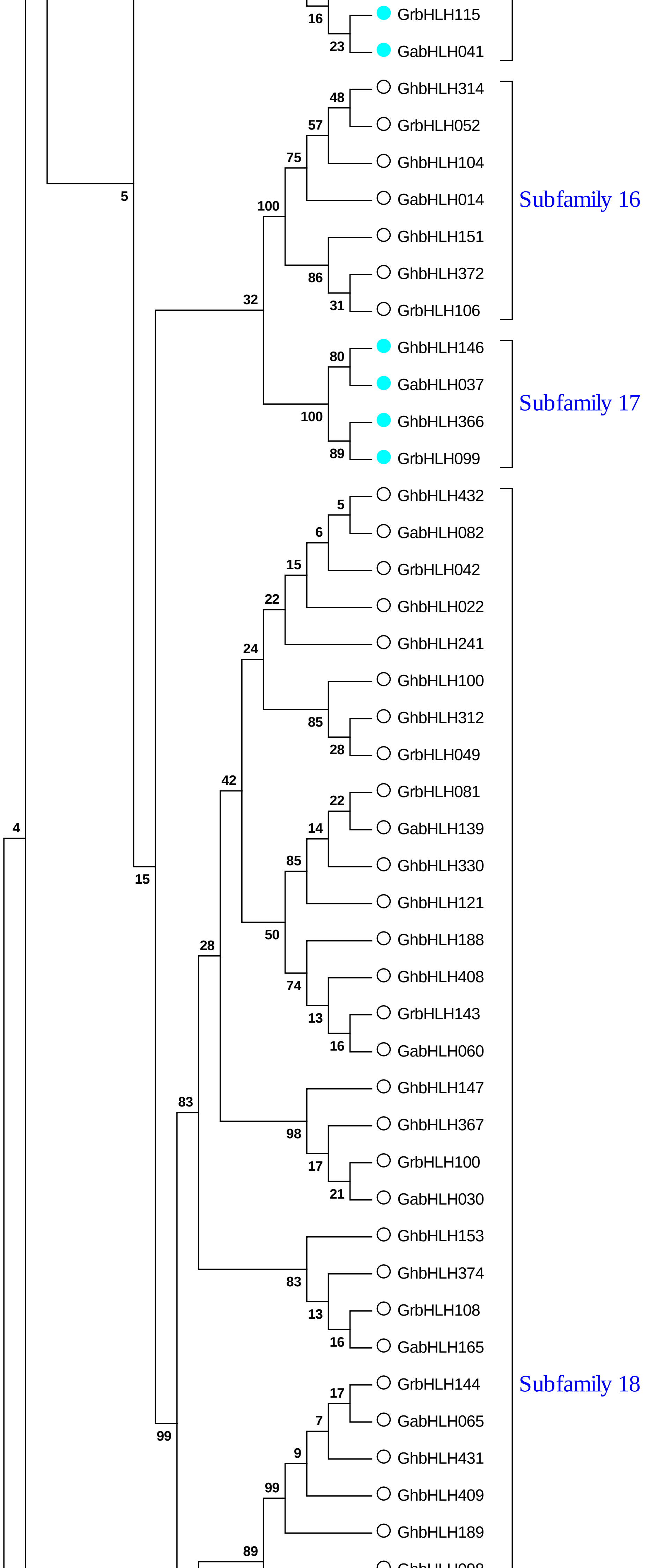
GrbHLH115
GabHLH041
GhbHLH314
GrbHLH052
GhbHLH104
GabHLH014
Subfamily 16
GhbHLH151
GhbHLH372
GrbHLH106
GhbHLH146
GabHLH037
Subfamily 17
GhbHLH366
GrbHLH099
GhbHLH432
GabHLH082
GrbHLH042
GhbHLH022
GhbHLH241
GhbHLH100
GhbHLH312
GrbHLH049
GrbHLH081
GabHLH139
GhbHLH330
GhbHLH121
GhbHLH188
GhbHLH408
GrbHLH143
GabHLH060
GhbHLH147
GhbHLH367
GrbHLH100
GabHLH030
GhbHLH153
GhbHLH374
GrbHLH108
GabHLH165
GrbHLH144
Subfamily 18
GabHLH065
GhbHLH431
GhbHLH409
GhbHLH189

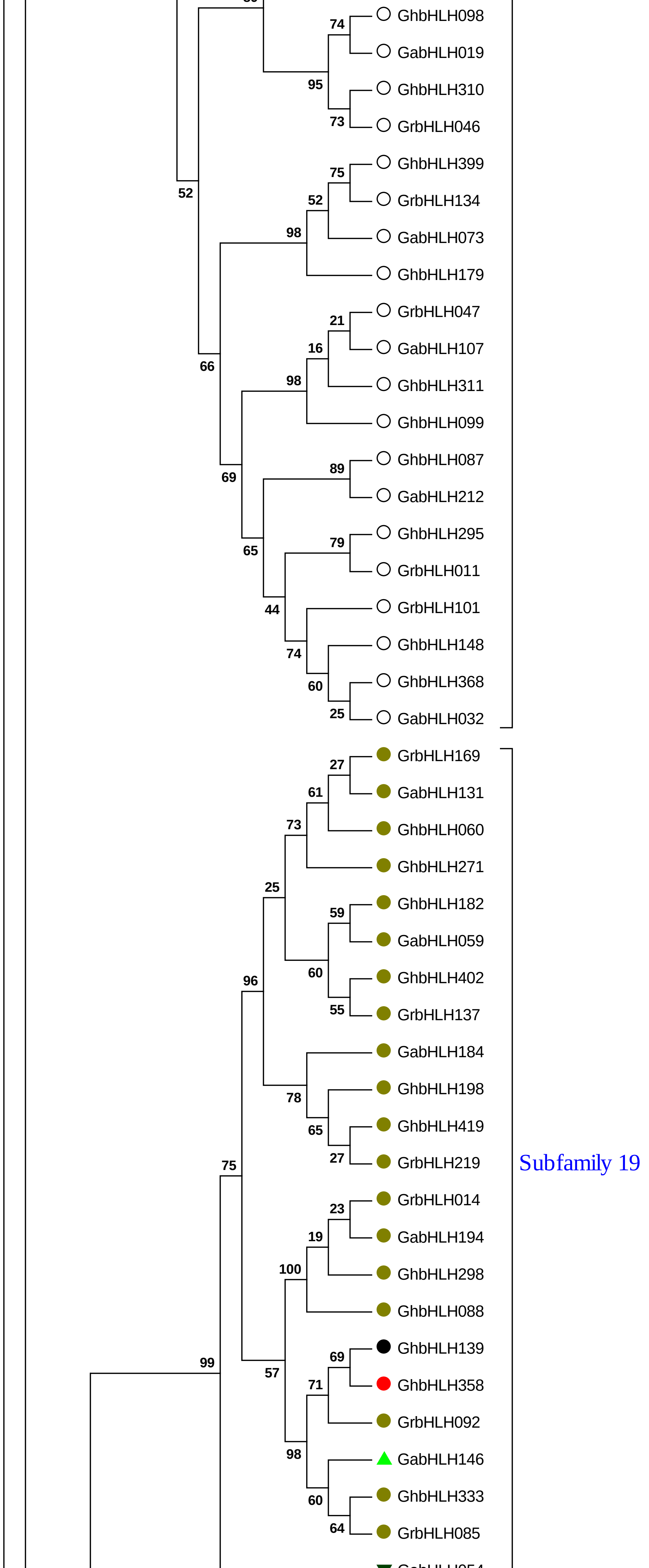

GhbHLH098
GabHLH019
GhbHLH310
GrbHLH046
GhbHLH399
GrbHLH134
GabHLH073
GhbHLH179
GrbHLH047
GabHLH107
GhbHLH311
GhbHLH099
GhbHLH087
GabHLH212
GhbHLH295
GrbHLH011
GrbHLH101
GhbHLH148
GhbHLH368
GabHLH032
GrbHLH169
GabHLH131
GhbHLH060
GhbHLH271
GhbHLH182
GabHLH059
GhbHLH402
GrbHLH137
GabHLH184
GhbHLH198
GhbHLH419
GrbHLH219
Subfamily 19
GrbHLH014
GabHLH194
GabHLH298
GhbHLH088
GhbHLH139
GhbHLH358
GrbHLH092
GabHLH146
GhbHLH333
GrbHLH085

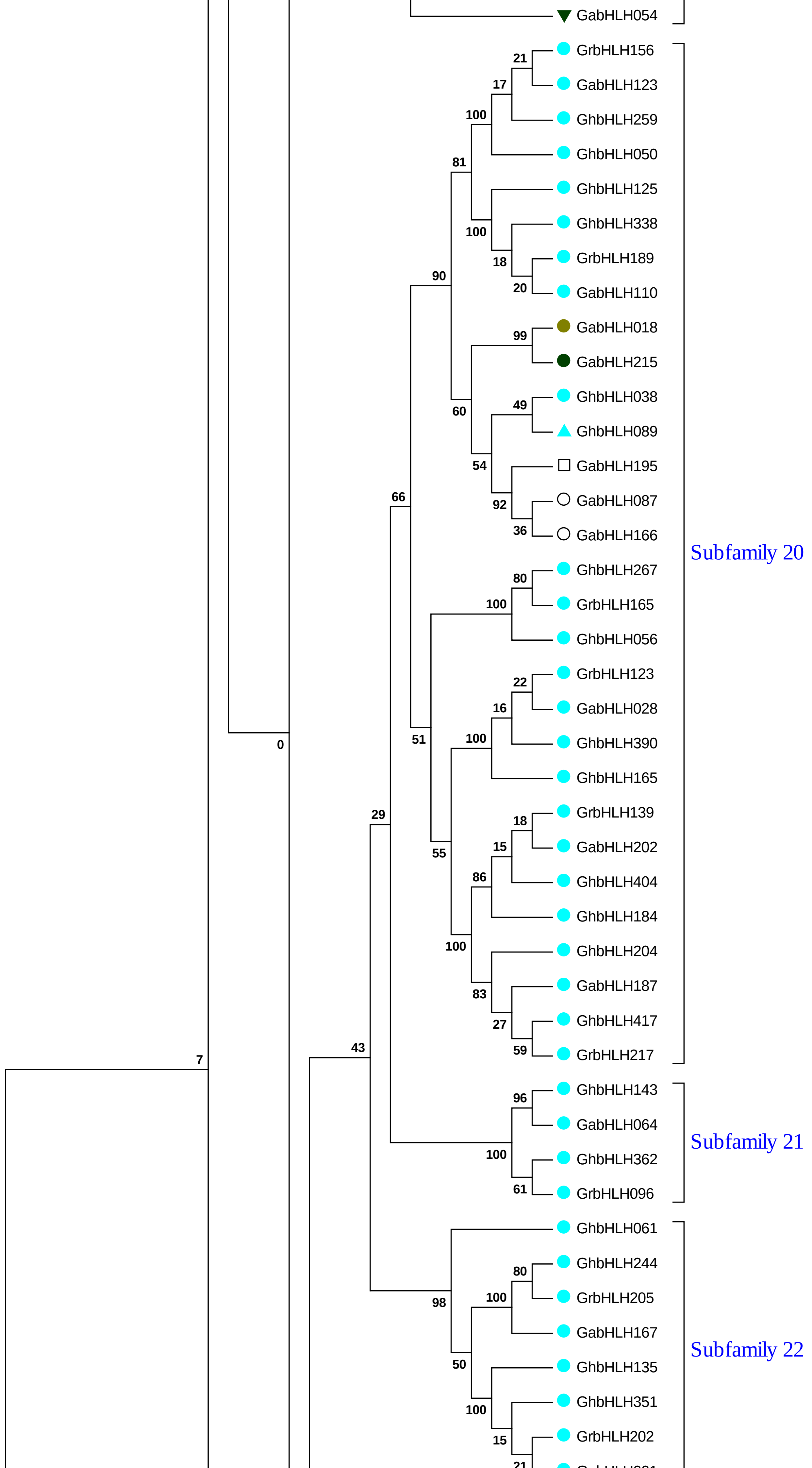
GabHLH054
GrbHLH156
GabHLH123
GhbHLH259
GhbHLH050
GhbHLH125
GhbHLH338
GrbHLH189
GabHLH110
GabHLH018
GabHLH215
GhbHLH038
GhbHLH089
GabHLH195
GabHLH087
GabHLH166
GhbHLH267
GrbHLH165
GhbHLH056
GrbHLH123
GabHLH028
GhbHLH390
GabHLH165
GrbHLH139
GabHLH202
GhbHLH404
GhbHLH184
GhbHLH204
GabHLH187
GhbHLH417
GrbHLH217
Subfamily 20
GhbHLH143
GabHLH064
GhbHLH362
GrbHLH096
Subfamily 21
GhbHLH061
GhbHLH244
GrbHLH205
GabHLH167
GhbHLH135
GhbHLH351
GrbHLH202
Subfamily 22

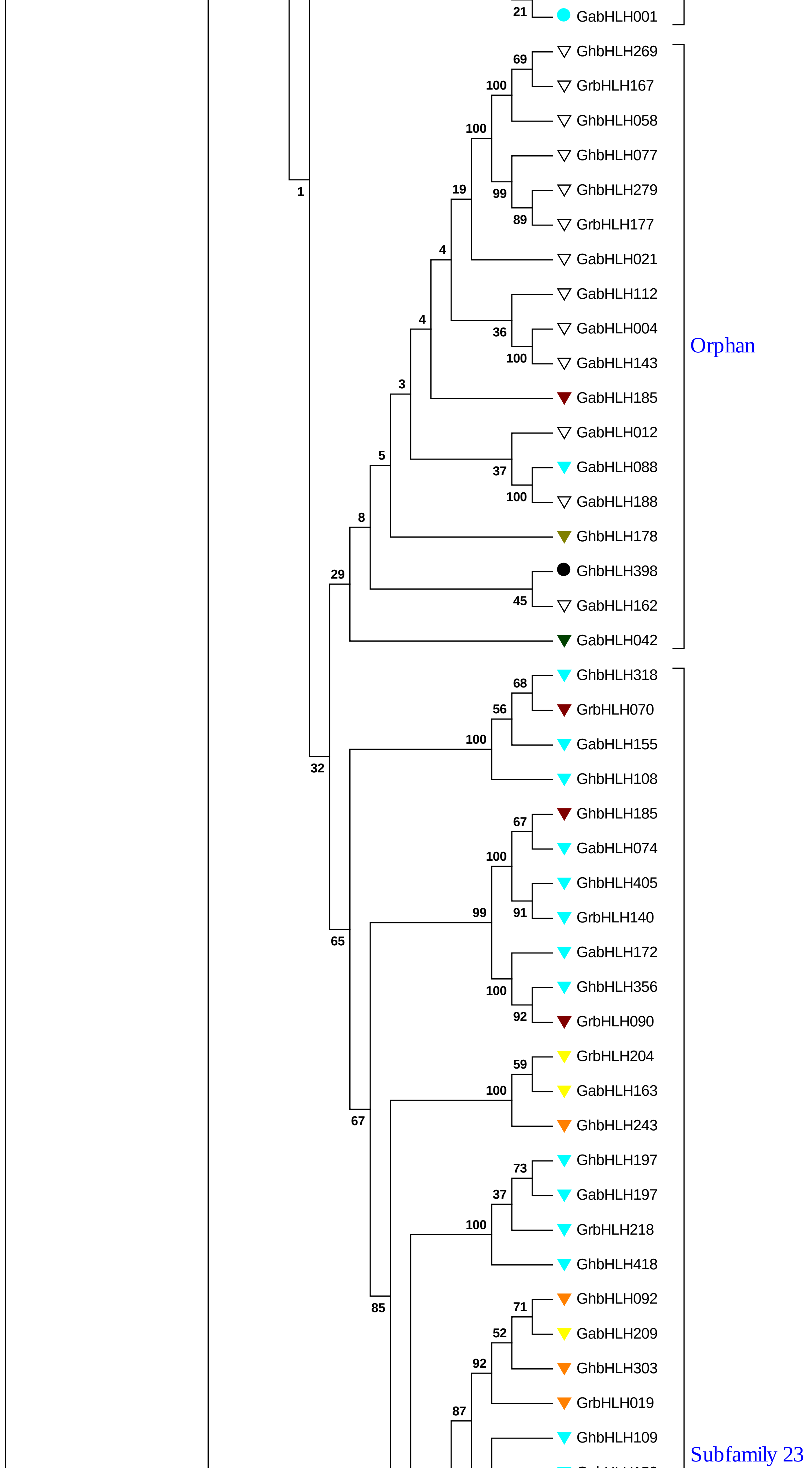

GabHLH001
GhbHLH269
GrbHLH167
GhbHLH058
GhbHLH077
GhbHLH279
GrbHLH177
GabHLH021
GabHLH112
GabHLH004
GabHLH143
GabHLH185
GabHLH012
GabHLH088
GabHLH188
GhbHLH178
GhbHLH398
GabHLH162
GabHLH042
Orphan
GhbHLH318
GrbHLH070
GabHLH155
GhbHLH108
GhbHLH185
GabHLH074
GhbHLH405
GrbHLH140
GabHLH172
GhbHLH356
GrbHLH090
GrbHLH204
GabHLH163
GhbHLH243
GhbHLH197
GabHLH197
GrbHLH218
GhbHLH418
GhbHLH092
GabHLH209
GhbHLH303
GrbHLH019
GhbHLH109
Subfamily 23

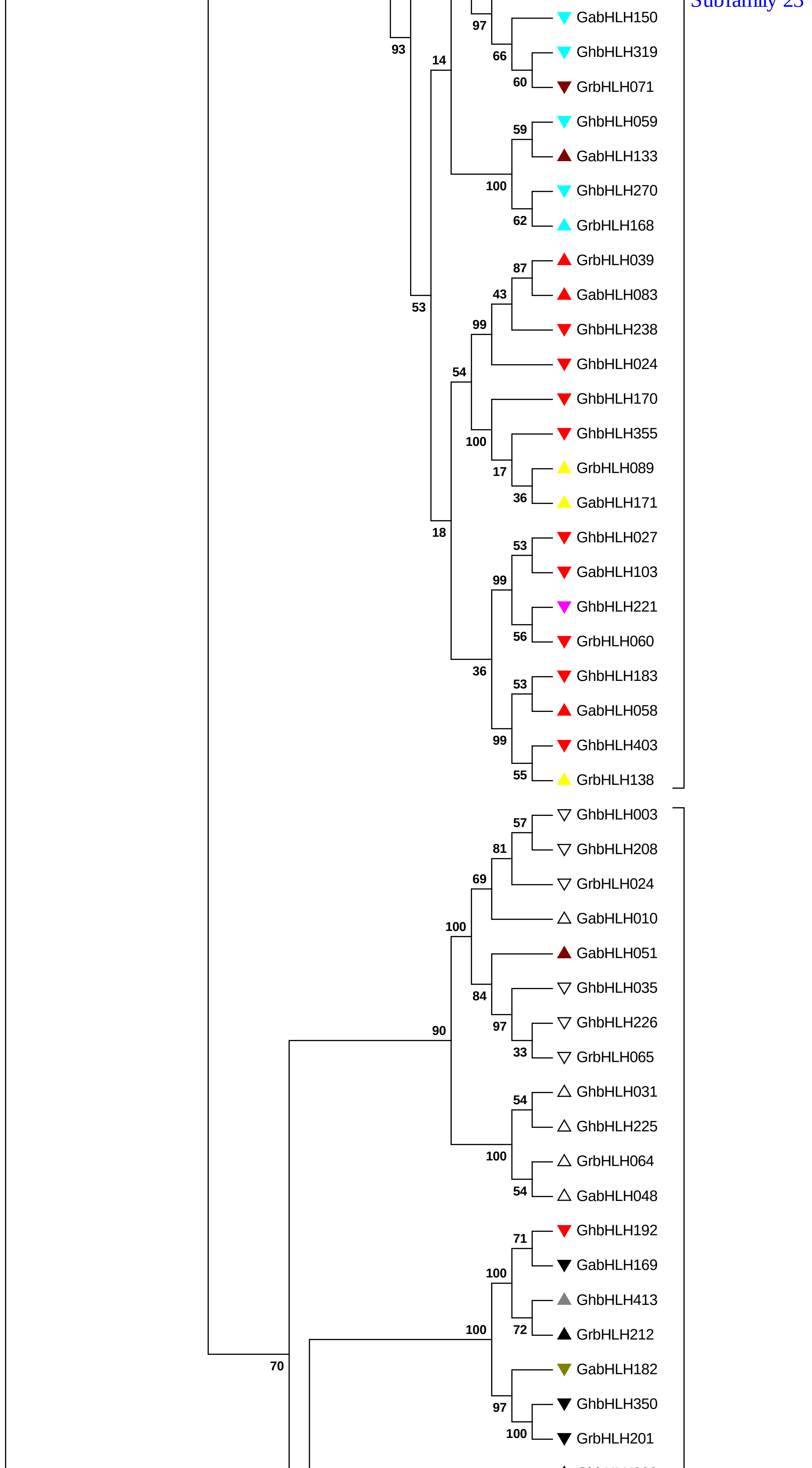
GabHLH150
GhbHLH319
GrbHLH071
GhbHLH059
GabHLH133
GhbHLH270
GrbHLH168
GrbHLH039
GabHLH083
GhbHLH238
GhbHLH024
GhbHLH170
GhbHLH355
GrbHLH089
GabHLH171
GhbHLH027
GabHLH103
GhbHLH221
GrbHLH060
GhbHLH183
GabHLH058
GhbHLH403
GrbHLH138
GhbHLH003
GhbHLH208
GrbHLH024
GabHLH010
GabHLH051
GhbHLH035
GhbHLH226
GrbHLH065
GhbHLH031
GhbHLH225
GrbHLH064
GabHLH048
GhbHLH192
GabHLH169
GhbHLH413
GrbHLH212
GabHLH182
GhbHLH350
GrbHLH201

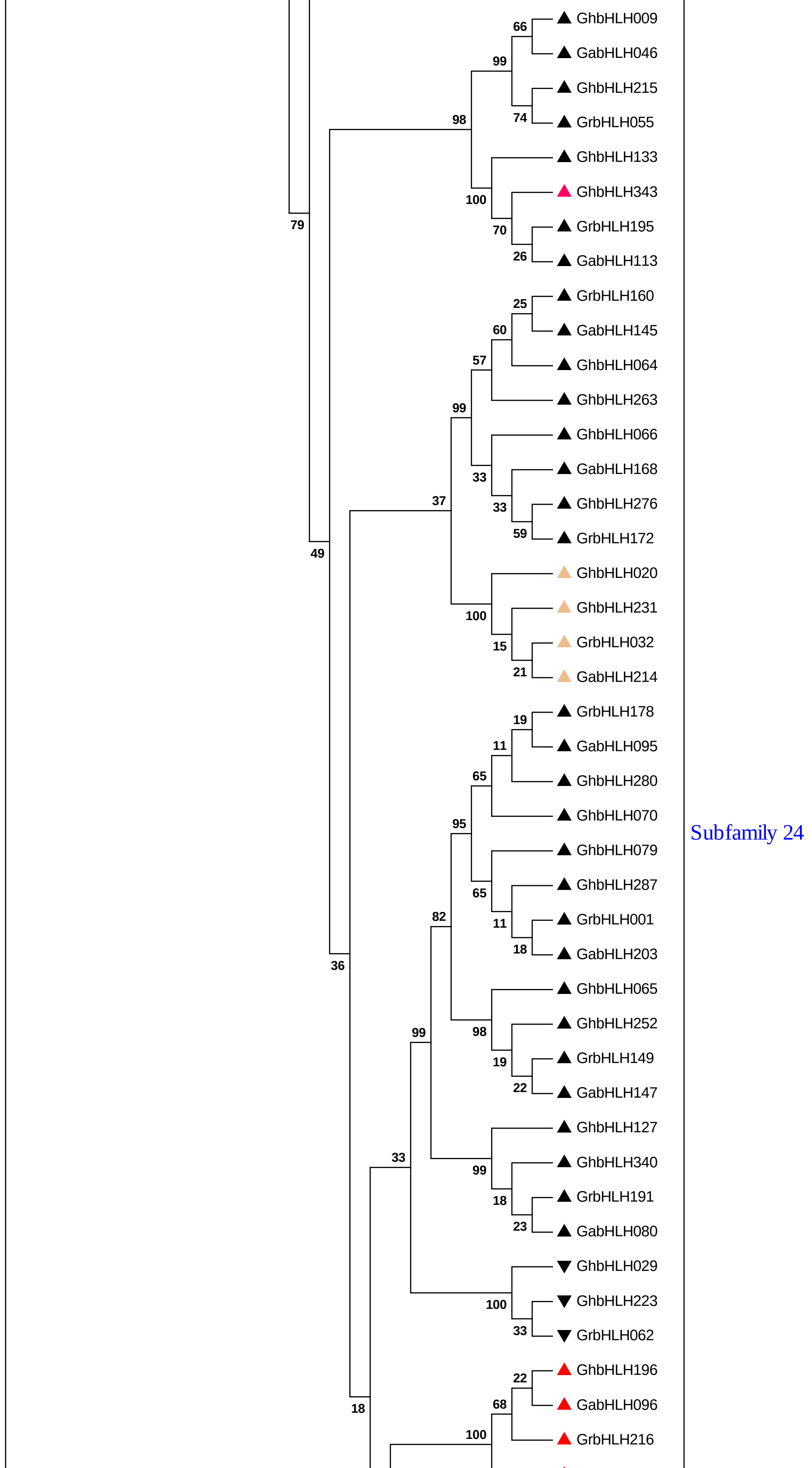

GhbHLH009
GabHLH046
GhbHLH215
GrbHLH055
GhbHLH133
GhbHLH343
GrbHLH195
GabHLH113
GrbHLH160
GabHLH145
GhbHLH064
GhbHLH263
GhbHLH066
GabHLH168
GhbHLH276
GrbHLH172
GhbHLH020
GhbHLH231
GrbHLH032
GabHLH214
GrbHLH178
GabHLH095
GhbHLH280
GhbHLH070
GhbHLH079
GhbHLH287
GrbHLH001
GabHLH203
GhbHLH065
GhbHLH252
GrbHLH149
GabHLH147
GhbHLH127
GhbHLH340
GrbHLH191
GabHLH080
GhbHLH029
GhbHLH223
GrbHLH062
GhbHLH196
GabHLH096
GrbHLH216
Subfamily 24

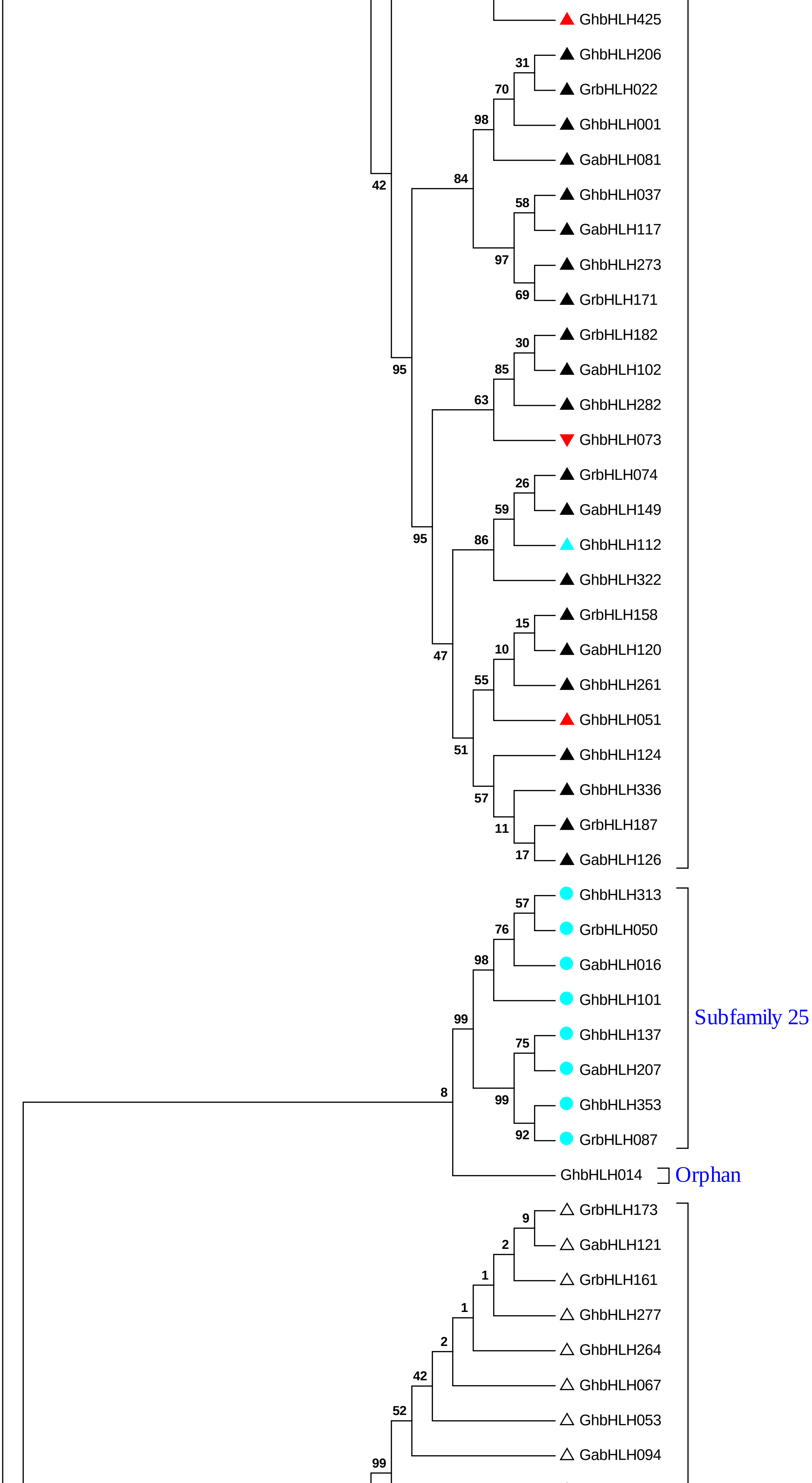
GhbHLH425
GhbHLH206
GrbHLH022
GhbHLH001
GabHLH081
GhbHLH037
GabHLH117
GhbHLH273
GrbHLH171
GrbHLH182
GabHLH102
GhbHLH282
GhbHLH073
GrbHLH074
GabHLH149
GhbHLH112
GhbHLH322
GrbHLH158
GabHLH120
GhbHLH261
GhbHLH051
GhbHLH124
GhbHLH336
GrbHLH187
GabHLH126
GhbHLH313
GrbHLH050
GabHLH016
GhbHLH101
GhbHLH137
GabHLH207
GhbHLH353
GrbHLH087
Subfamily 25
GhbHLH014
Orphan
GrbHLH173
GabHLH121
GrbHLH161
GhbHLH277
GhbHLH264
GhbHLH067
GhbHLH053
GabHLH094

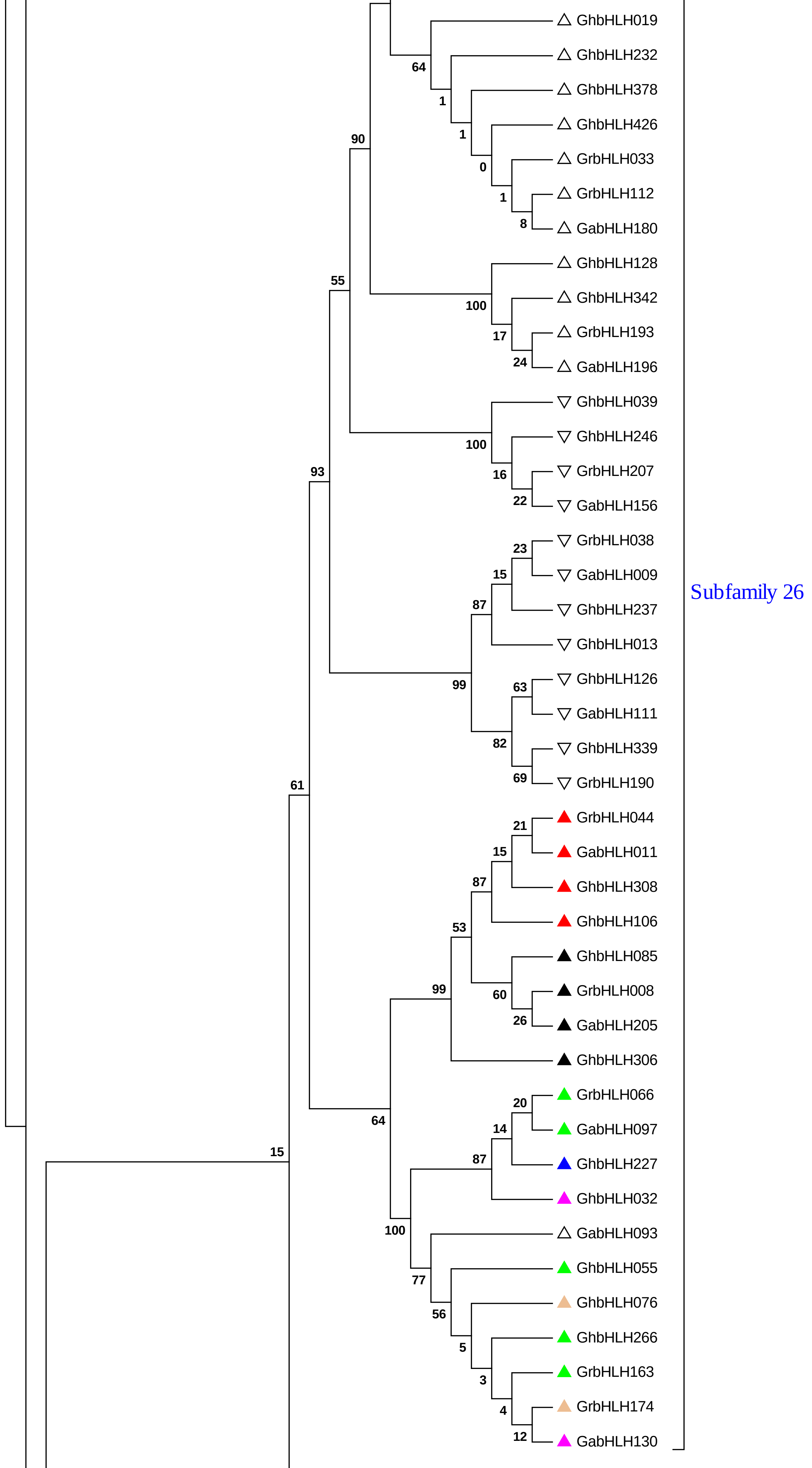

GhbHLH019
GhbHLH232
GhbHLH378
GhbHLH426
GrbHLH033
GrbHLH112
GabHLH180
GhbHLH128
GhbHLH342
GrbHLH193
GabHLH196
GhbHLH039
GhbHLH246
GrbHLH207
GabHLH156
GrbHLH038
GabHLH009
GhbHLH237
GhbHLH013
GhbHLH126
GabHLH111
GhbHLH339
GrbHLH190
GrbHLH044
GabHLH011
GhbHLH308
GhbHLH106
GhbHLH085
GrbHLH008
GabHLH205
GhbHLH306
GrbHLH066
GabHLH097
GhbHLH227
GhbHLH032
GabHLH093
GhbHLH055
GhbHLH076
GhbHLH266
GrbHLH163
GrbHLH174
GabHLH130
Subfamily 26

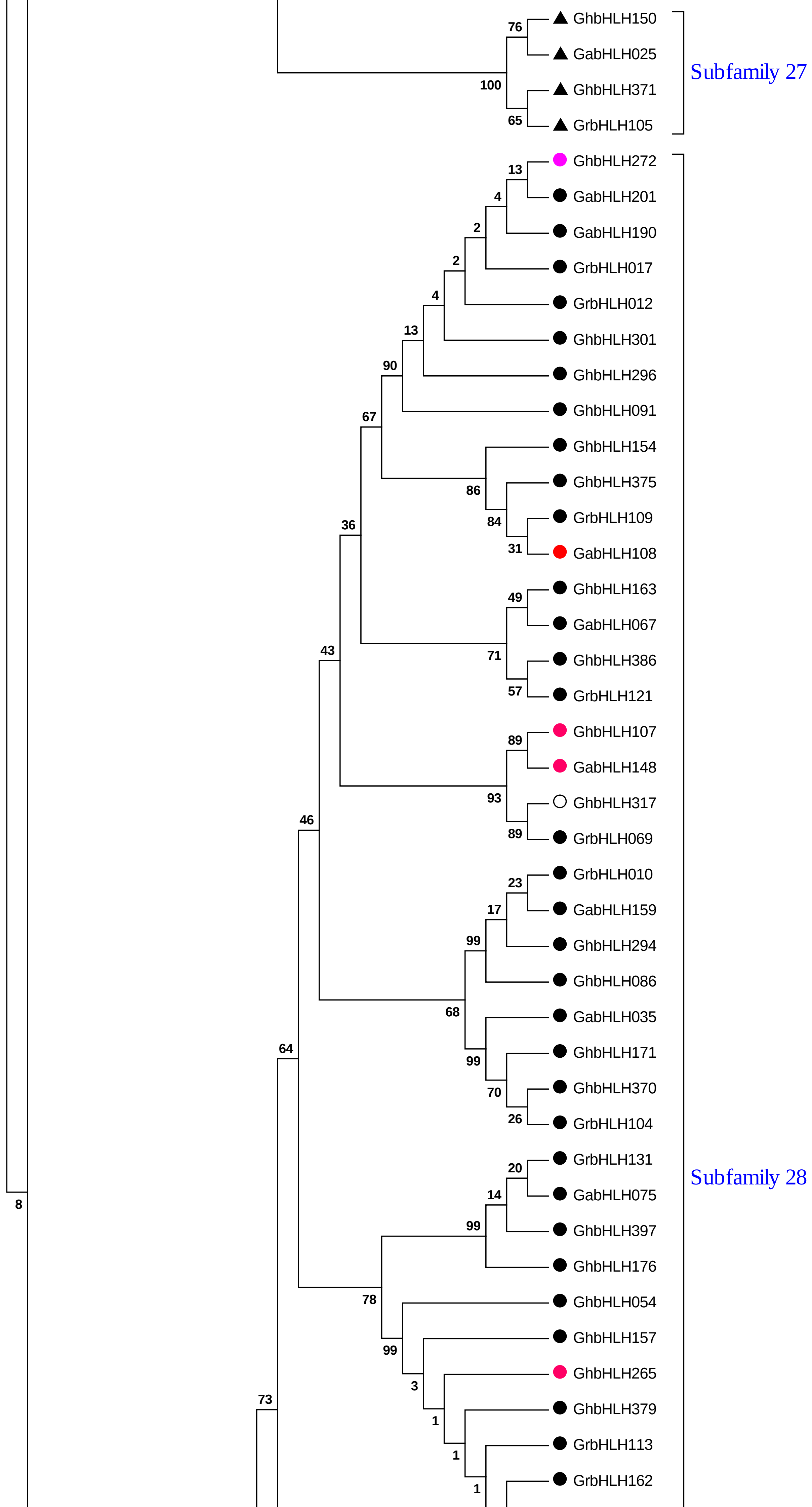
GhbHLH150
GabHLH025
GhbHLH371
GrbHLH105
Subfamily 27
GhbHLH272
GabHLH201
GabHLH190
GrbHLH017
GrbHLH012
GhbHLH301
GhbHLH296
GhbHLH091
GhbHLH154
GhbHLH375
GrbHLH109
GabHLH108
GhbHLH163
GabHLH067
GhbHLH386
GrbHLH121
GhbHLH107
GabHLH148
GhbHLH317
GrbHLH069
GrbHLH010
GabHLH159
GhbHLH294
GhbHLH086
GabHLH035
GhbHLH171
GhbHLH370
GrbHLH104
GrbHLH131
GabHLH075
GhbHLH397
GhbHLH176
GhbHLH054
GhbHLH157
GhbHLH265
GhbHLH379
GrbHLH113
GrbHLH162
Subfamily 28

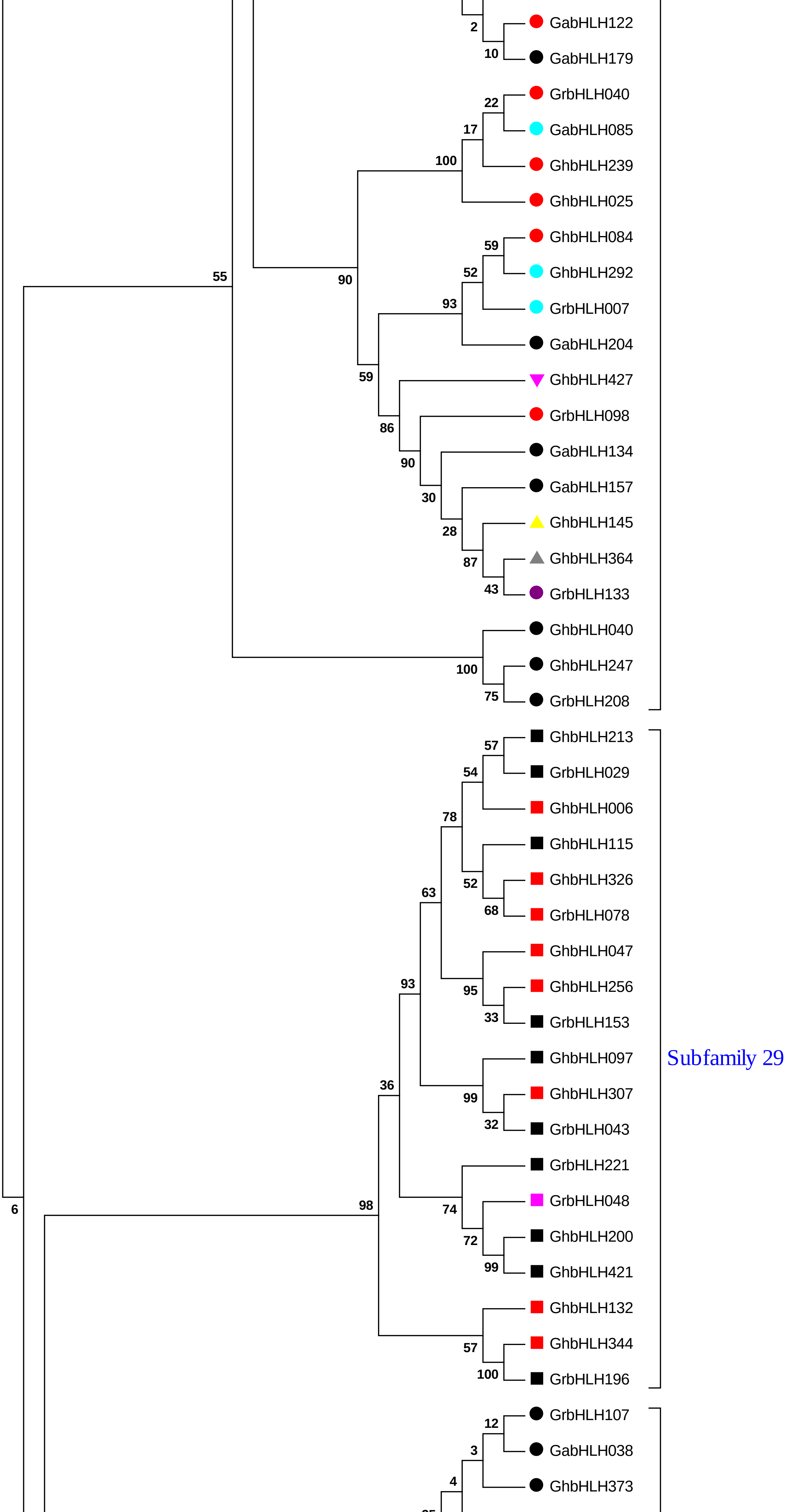
GabHLH122
GabHLH179
GrbHLH040
GabHLH085
GhbHLH239
GhbHLH025
GhbHLH084
GhbHLH292
GrbHLH007
GabHLH204
GhbHLH427
GrbHLH098
GabHLH134
GabHLH157
GhbHLH145
GhbHLH364
GrbHLH133
GhbHLH040
GhbHLH247
GrbHLH208
GhbHLH213
GrbHLH029
GhbHLH006
GhbHLH115
GhbHLH326
GrbHLH078
GhbHLH047
GhbHLH256
GrbHLH153
GhbHLH097
GhbHLH307
GrbHLH043
GrbHLH221
GrbHLH048
GhbHLH200
GhbHLH421
GhbHLH132
GhbHLH344
GrbHLH196
Subfamily 29
GrbHLH107
GabHLH038
GhbHLH373

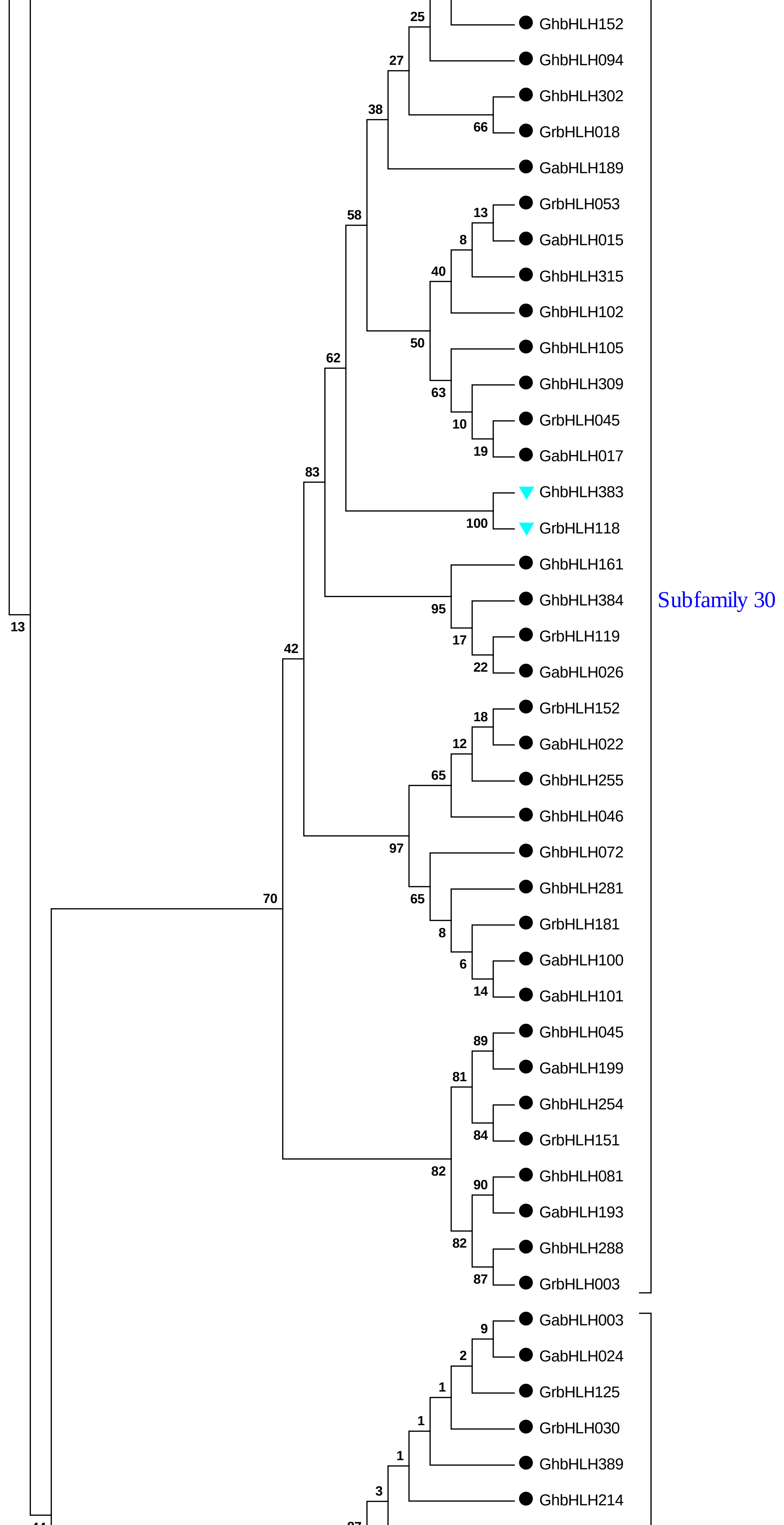
GhbHLH152
GhbHLH094
GhbHLH302
GrbHLH018
GabHLH189
GrbHLH053
GabHLH015
GhbHLH315
GhbHLH102
GhbHLH105
GhbHLH309
GrbHLH045
GabHLH017
GhbHLH383
GrbHLH118
GhbHLH161
GhbHLH384
GrbHLH119
GabHLH026
GrbHLH152
GabHLH022
GhbHLH255
GhbHLH046
GhbHLH072
GhbHLH281
GrbHLH181
GabHLH100
GabHLH101
GhbHLH045
GabHLH199
GhbHLH254
GrbHLH151
GhbHLH081
GabHLH193
GhbHLH288
GrbHLH003
GabHLH003
GabHLH024
GrbHLH125
GrbHLH030
GhbHLH389
GhbHLH214
Subfamily 30

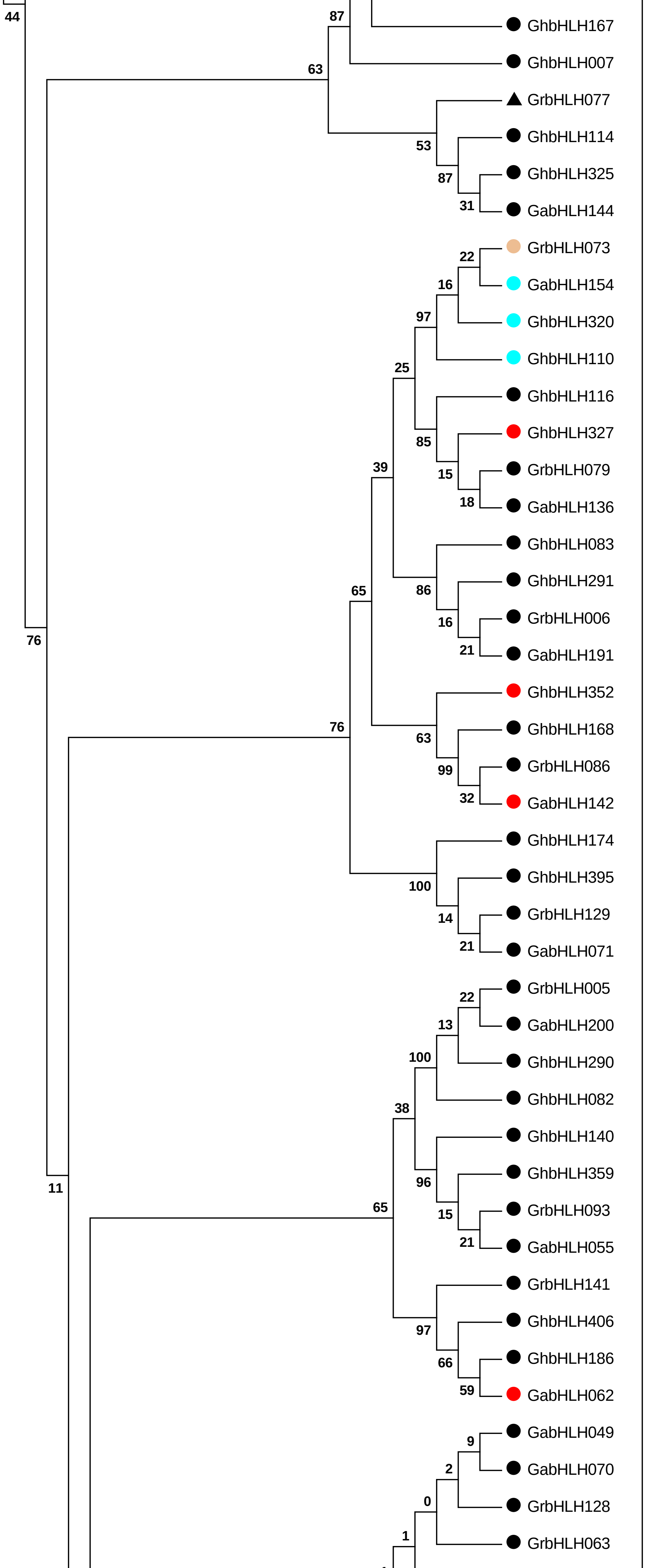
GhbHLH167
GhbHLH007
GrbHLH077
GhbHLH114
GhbHLH325
GabHLH144
GrbHLH073
GabHLH154
GhbHLH320
GhbHLH110
GhbHLH116
GhbHLH327
GrbHLH079
GabHLH136
GhbHLH083
GhbHLH291
GrbHLH006
GabHLH191
GhbHLH352
GhbHLH168
GrbHLH086
GabHLH142
GhbHLH174
GhbHLH395
GrbHLH129
GabHLH071
GrbHLH005
GabHLH200
GhbHLH290
GhbHLH082
GhbHLH140
GhbHLH359
GrbHLH093
GabHLH055
GrbHLH141
GhbHLH406
GhbHLH186
GabHLH062
GabHLH049
GabHLH070
GrbHLH128
GrbHLH063

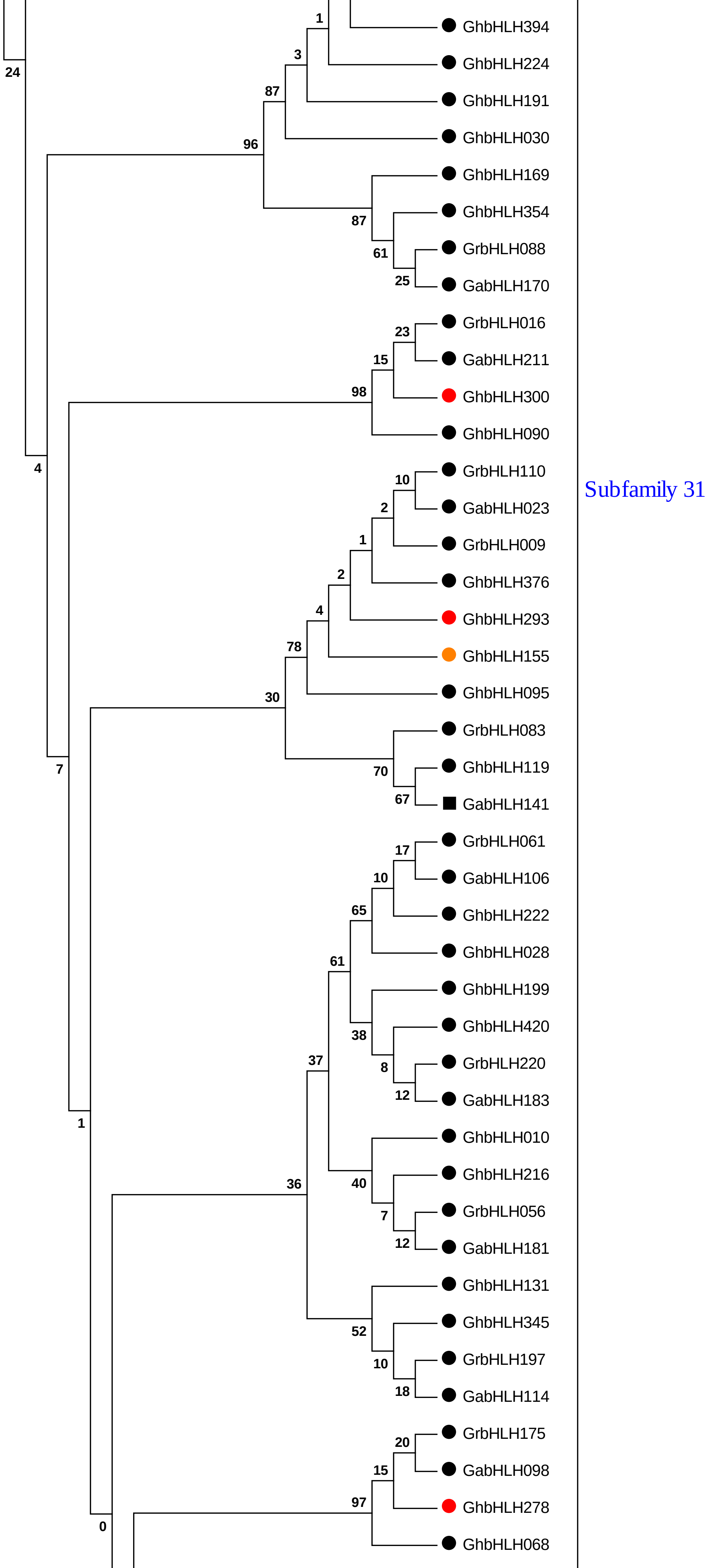
GhbHLH394
GhbHLH224
GhbHLH191
GhbHLH030
GhbHLH169
GhbHLH354
GrbHLH088
GabHLH170
GrbHLH016
GabHLH211
GhbHLH300
GhbHLH090
GrbHLH110
GabHLH023
GrbHLH009
GhbHLH376
GhbHLH293
GhbHLH155
GhbHLH095
GrbHLH083
GhbHLH119
GabHLH141
GrbHLH061
GabHLH106
GhbHLH222
GhbHLH028
GhbHLH199
GhbHLH420
GrbHLH220
GabHLH183
GhbHLH010
GhbHLH216
GrbHLH056
GabHLH181
GhbHLH131
GhbHLH345
GrbHLH197
GabHLH114
GrbHLH175
GabHLH098
GhbHLH278
GhbHLH068
Subfamily 31

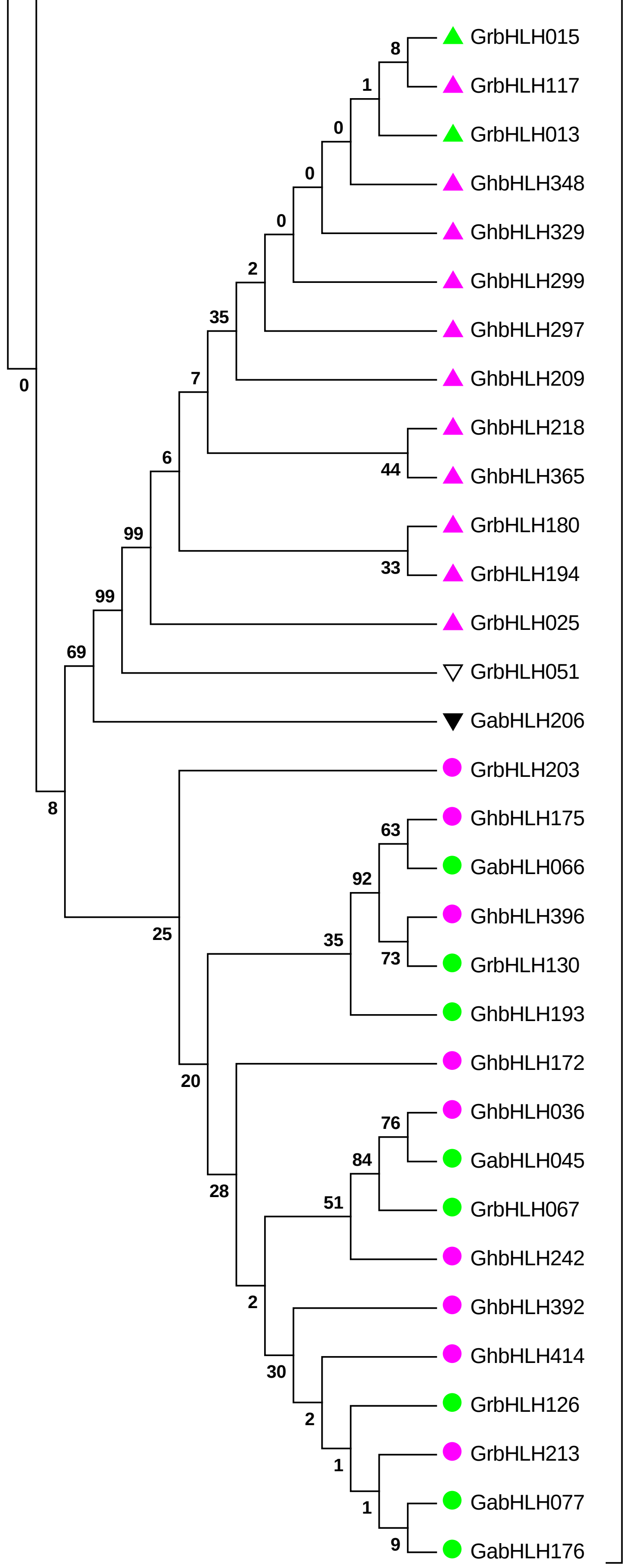
GrbHLH015
GrbHLH117
GrbHLH013
GhbHLH348
GhbHLH329
GhbHLH299
GhbHLH297
GhbHLH209
GhbHLH218
GhbHLH365
GrbHLH180
GrbHLH194
GrbHLH025
GrbHLH051
GabHLH206
GrbHLH203
GhbHLH175
GabHLH066
GhbHLH396
GrbHLH130
GhbHLH193
GhbHLH172
GhbHLH036
GabHLH045
GrbHLH067
GhbHLH242
GhbHLH392
GhbHLH414
GrbHLH126
GrbHLH213
GabHLH077
GabHLH176
