# Supplementary material for: Divergence and evolution of cotton bHLH proteins from diploid to allotetraploid
Source: BMC Genomics. 2018 Feb 23;19:162. doi: 10.1186/s12864-018-4543-y (PMC5824590; doi:10.1186/s12864-018-4543-y)
Supplement: Supplementary file 7 — Multiple sequence alignment of the bHLH domains of the 871 members of the cotton bHLH protein family. (PDF 392 kb) [file 12864_2018_4543_MOESM7_ESM.pdf]

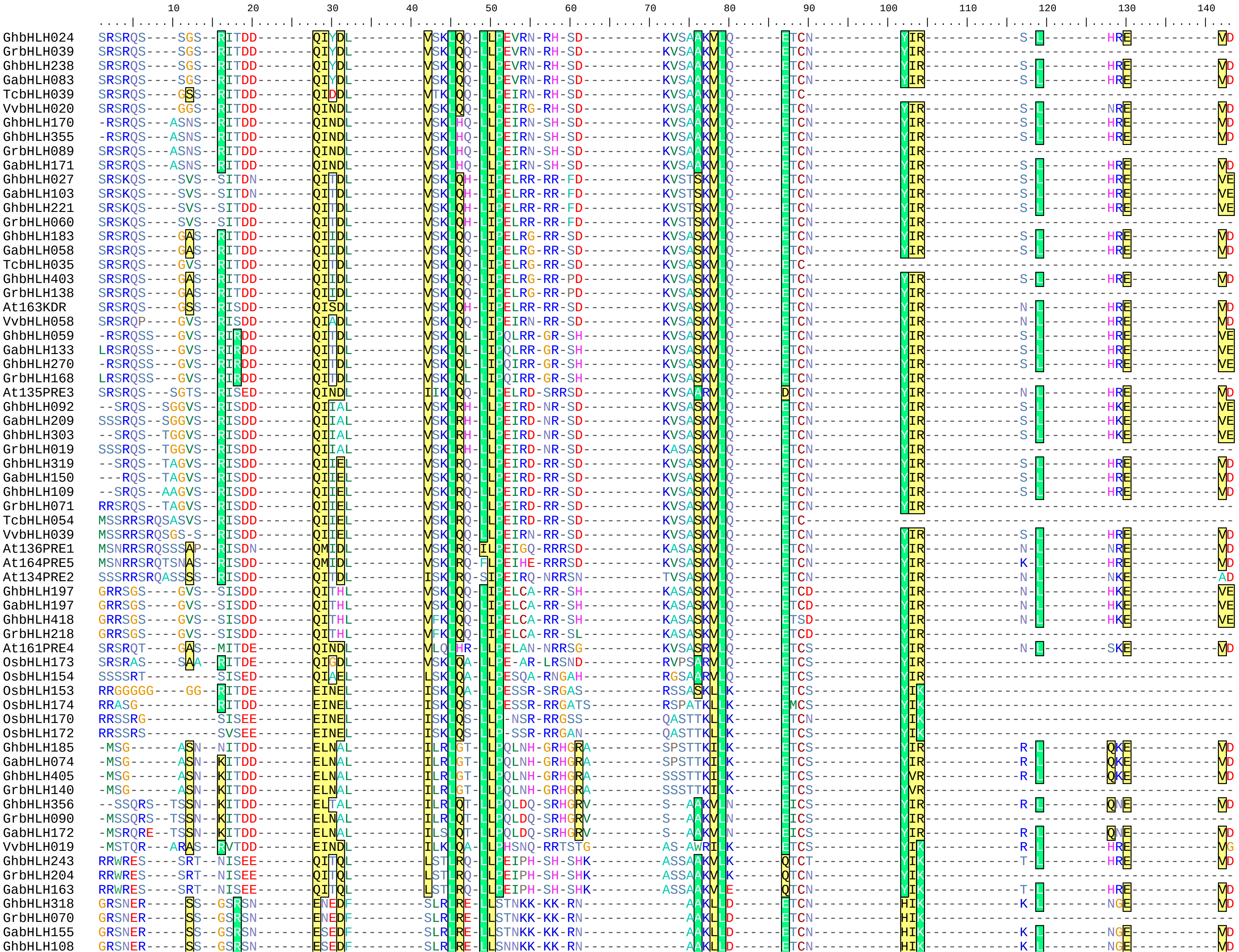

10 20 30 40 50 60 70 80 90 100 110 120 130 140
GhbHLH024
GrbHLH039
GhbHLH238
GabHLH083
TcbHLH039
VvbHLH020
GhbHLH170
GhbHLH355
GrbHLH089
GabHLH171
GhbHLH027
GabHLH103
GhbHLH221
GrbHLH060
GhbHLH183
GabHLH058
TcbHLH035
GhbHLH403
GrbHLH138
At163KDR
VvbHLH058
GhbHLH059
GabHLH133
GhbHLH270
GrbHLH168
At135PRE3
GhbHLH092
GabHLH209
GhbHLH303
GrbHLH019
GhbHLH319
GabHLH150
GhbHLH109
GrbHLH071
TcbHLH054
VvbHLH039
At136PRE1
At164PRE5
At134PRE2
GhbHLH197
GabHLH197
GhbHLH418
GrbHLH218
At161PRE4
OsbHLH173
OsbHLH154
OsbHLH153
OsbHLH174
OsbHLH170
OsbHLH172
GhbHLH185
GabHLH074
GhbHLH405
GrbHLH140
GhbHLH356
GrbHLH090
GabHLH172
VvbHLH019
GhbHLH243
GrbHLH204
GabHLH163
GhbHLH318
GrbHLH070
GabHLH155
GhbHLH108

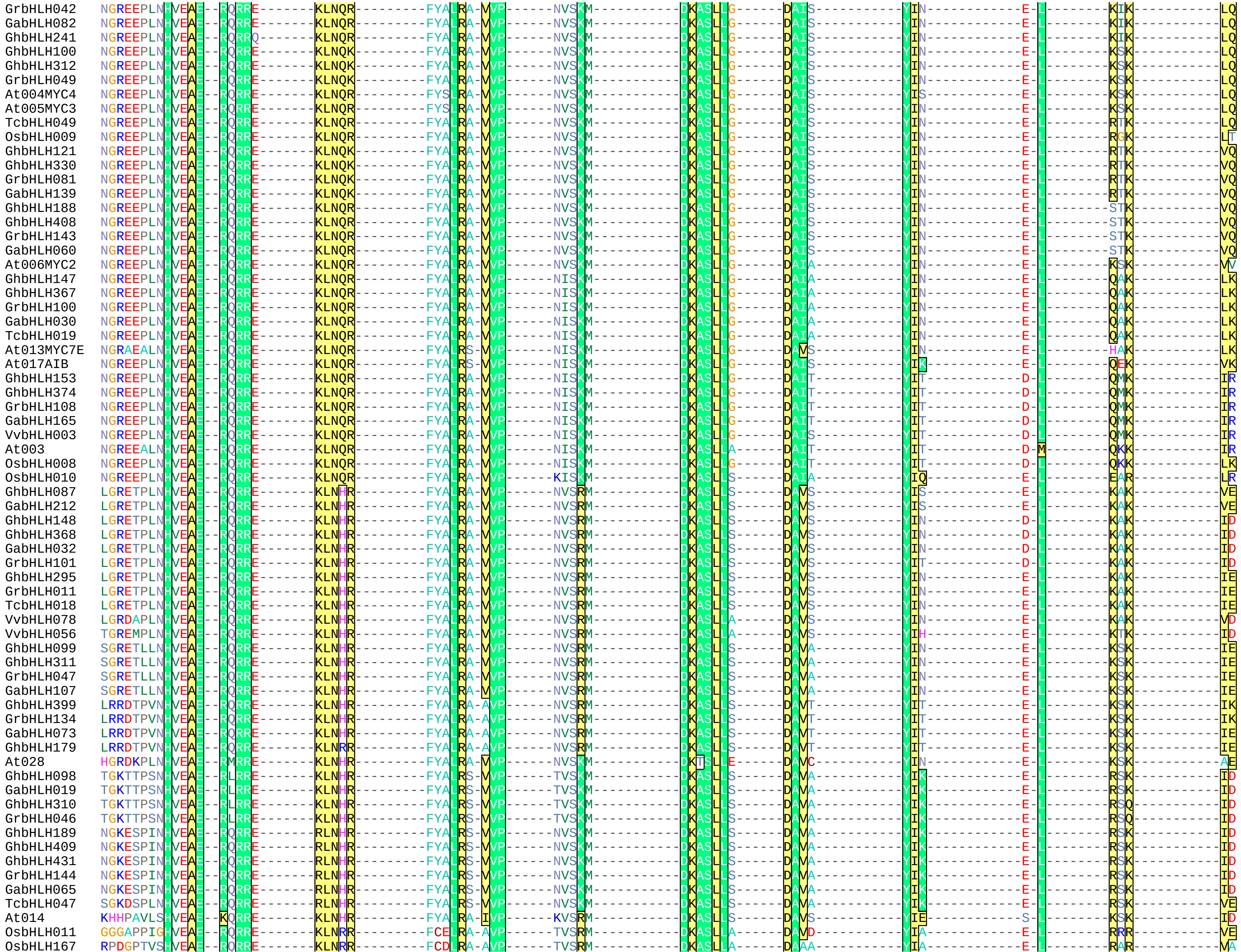
GrbHLH042
GabHLH082
GhbHLH241
GhbHLH100
GhbHLH312
GrbHLH049
At004MYC4
At005MYC3
TcbHLH049
OsbHLH009
GhbHLH121
GhbHLH330
GrbHLH081
GabHLH139
GhbHLH188
GhbHLH408
GrbHLH143
GabHLH060
At006MYC2
GhbHLH147
GhbHLH367
GrbHLH100
GabHLH030
TcbHLH019
At013MYC7E
At017AIB
GhbHLH153
GhbHLH374
GrbHLH108
GabHLH165
VvbHLH003
At003
OsbHLH008
OsbHLH010
GhbHLH087
GabHLH212
GhbHLH148
GhbHLH368
GabHLH032
GrbHLH101
GhbHLH295
GrbHLH011
TcbHLH018
VvbHLH078
VvbHLH056
GhbHLH099
GhbHLH311
GrbHLH047
GabHLH107
GhbHLH399
GrbHLH134
GabHLH073
GhbHLH179
At028
GhbHLH098
GabHLH019
GhbHLH310
GrbHLH046
GhbHLH189
GhbHLH409
GhbHLH431
GrbHLH144
GabHLH065
TcbHLH047
At014
OsbHLH011
OsbHLH167

```
GrbHLH166   ELENQRMTHIAVE--RNRRK-------QMNEY---------LAAIRS-LMPAS-----YVQ----------RGDQASIIG------GAIN-----------FVK------------E-L--------EQL-----------LQ
TcbHLH078   ELENQRMTHIAVE--RNRRK-------QMNEY---------LAVLRS-LMPPS-----YVQ----------RGDQASIIG------GAIN-----------FVK------------E-L--------EQL-----------LQ
VvbHLH026   EVENQRMTHIAVE--RNRRK-------QMNEY---------LAVLRS-LMPPS-----YTQ----------RGDQASIIG------GAIN-----------FVK------------E-L--------EQL-----------LQ
GhbHLH202   EIENQRMTHITVE--RNRRK-------QMNQY---------LSVLRS-LMPPS-----YAQ----------RGDQASIIG------GSIN-----------FVK------------E-L--------EQR-----------LQ
GabHLH178   EIENQRMTHITVE--RNRRK-------QMNQY---------LSVLRS-LMPPS-----YAQ----------RGDQASIIG------GSIN-----------FVK------------E-L--------EQR-----------LQ
GhbHLH423   EMENQRMTHITVE--RNRRK-------QMNQY---------LSLLRS-LMPPS-----YAQ----------RGDQASIIG------GAIN-----------FVK------------E-L--------EQR-----------LQ
GrbHLH223   EMENQRMTHITVE--RNRRK-------QMNQY---------LSLLRS-LMPPS-----YAQ----------RGDQASIIG------GAIN-----------FVK------------E-L--------EQR-----------LQ
GhbHLH103   EIENQRMTHIAVE--RNRRK-------QMNDY---------LSVLRS-LMPES-----YVQ----------RGDQASIIG------GAIN-----------FVK------------E-L--------EHR-----------LQ
GhbHLH316   EIENQRMTHIAVE--RNRRK-------QMNDY---------LSVLRS-LMPES-----YVQ----------RGDQASIIG------GAIN-----------FVK------------E-L--------EHR-----------LQ
GrbHLH054   EIENQRMTHIAVE--RNRRK-------QMNDY---------LSVLRS-LMPES-----YVQ----------RGDQASIIG------GAIN-----------FVK------------E-L--------EHR-----------LQ
GabHLH013   EIENQRMTHIAVE--RNRRK-------QMNDY---------LSVLRS-LMPES-----YVQ----------RGDQASIIG------GAIN-----------FVK------------E-L--------EHR-----------LQ
TcbHLH069   EIENQRMTHIAVE--RNRRK-------QMNEY---------LSVLRS-LMPES-----YVQ----------RGDQASIIG------GAIN-----------FVK------------E-L--------EHR-----------LQ
VvbHLH001   EIENQRMTHIAVE--RNRRK-------QMNEY---------LSVLRS-LMPES-----YVQ----------RGDQASIIG------GAIN-----------FVK------------E-L--------EQR-----------LQ
GhbHLH162   EIENQRMTHIAVE--RNRRR-------QMNEY---------LSVLRS-LMPES-----YVQ----------RGDQASIIG------GAIN-----------FVK------------E-L--------EHC-----------VQ
GhbHLH385   EIENQRMTHIAVE--RNRRR-------QMNEY---------LSVLRS-LMPES-----YVQ----------RGDQASIIG------GAIN-----------FVK------------E-L--------EHC-----------VQ
GrbHLH120   EIENQRMTHIAVE--RNRRR-------QMNEY---------LSVLRS-LMPES-----YVQ----------RGDQASIIG------GAIN-----------FVK------------E-L--------EHC-----------VQ
GabHLH186   EIENQRMTHIAVE--RNRRR-------QMNEY---------LSVLRS-LMPES-----YVQ----------RGDQASIIG------GAIN-----------FVK------------E-L--------EHC-----------VQ
GhbHLH015   EIENQRMIHIAVE--RNRRR-------QMNDY---------LAVLRT-MMPNS-----YVQ----------RGDQASIIG------GAIN-----------FVK------------V-L--------EQI-----------LQ
GhbHLH236   EIENQRMIHIAVE--RNRRR-------QMNDY---------LAVLRT-MMPNS-----YVQ----------RGDQASIIG------GAIN-----------FVK------------V-L--------EQI-----------LQ
GrbHLH037   EIENQRMIHIAVE--RNRRR-------QMNDY---------LAVLRT-MMPNS-----YVQ----------RGDQASIIG------GAIN-----------FVK------------V-L--------EQI-----------LQ
GabHLH002   EIENQRMIHIAVE--RNRRR-------QMNDY---------LAVLRT-MMPNS-----YVQ----------RGDQASIIG------GAIN-----------FVK------------V-L--------EQI-----------LQ
GhbHLH341   EIEHQRMTHIAVE--RNRRK-------QMSDY---------LALLKS-MMPTS-----YVQ----------RGDQASIIG------GAIN-----------FVK------------E-L--------EQF-----------LQ
GrbHLH192   EIEHQRMTHIAVE--RNRRK-------QMSDY---------LGLLKS-MMPTS-----YVQ----------RGDQASIIG------GAIN-----------FVK------------E-L--------EQF-----------LQ
TcbHLH009   EIENQRMTHIAVE--RNRRR-------QMNDY---------LAVLKS-MMPTS-----YVQ----------RGDQASIIG------GAIN-----------FVK------------E-L--------EQL-----------LQ
At094       EIENQRMTHIAVE--RNRRK-------QMNEY---------LAVLRS-LMPSS-----YAQ----------RGDQASIVG------GAIN-----------YVK------------E-L--------EHI-----------LQ
At096       EIENQRMTHIAVE--RNRRK-------QMNEY---------LAVLRS-LMPPY-----YAQ----------RGDQASIVG------GAIN-----------YLK------------E-L--------EHH-----------LQ
VvbHLH006   QVENQRMTHIAVE--RNRRR-------QMNEH---------LAVLRS-LMPAS-----YVQ----------RGDQASIIG------GAIN-----------FVK------------E-L--------EQL-----------LQ
OsbHLH047   EVESQRMTHIAVE--RNRRK-------QMNEY---------LAVLRS-LMPAS-----YVQ----------RGDQASIIG------GAIN-----------YVK------------E-M--------EQL-----------LQ
OsbHLH044   EIECQRMTHIAVE--RNRRR-------QMNEY---------LAVLRS-LMPAS-----YSQ----------RGDQASIVG------GAIN-----------YVK------------E-L--------EQL-----------LQ
OsbHLH045   EIESQRMTHIAVE--RNRRR-------QMNEY---------LAVLRS-LMPPS-----YAQ----------RGDQASIVG------GAIN-----------YVR------------E-L--------EQL-----------LQ
OsbHLH046   EVESQRMTHIAVE--RNRRK-------QMNEY---------LAVLRS-LMPPS-----YAQ----------RGDQASIVG------GAIN-----------FVK------------E-L--------EQL-----------LQ
OsbHLH048   EAESQRRNHIAVE--RNRRR-------QMNEY---------LAVLRS-LMPPS-----YAQ----------RGDQASIVA------GAIN-----------FVK------------E-L--------EQL-----------LQ
GhbHLH118   EAETQRMTHIAVE--RNRRK-------QMNEH---------LTVLRS-LMPES-----YVQ----------RGDQASIVG------GAIE-----------FVK------------E-L--------EHL-----------LQ
GhbHLH331   EAETQRMTHIAVE--RNRRK-------QMNEH---------LTVLRS-LMPES-----YVQ----------RGDQASIVG------GAIE-----------FVK------------E-L--------EHL-----------LQ
GrbHLH082   EAETQRMTHIAVE--RNRRK-------QMNEH---------LTVLRS-LMPES-----YVQ----------RGDQASIVG------GAIE-----------FVK------------E-L--------EHL-----------LQ
GabHLH140   EAETQRMTHIAVE--RNRRK-------QMNEH---------LTVLRS-LMPES-----YVQ----------RGDQASIVG------GAIE-----------FVK------------E-L--------EHL-----------LQ
GhbHLH187   EAETQRMTHIAVE--RNRRK-------QMNEH---------LAVLRS-LMPES-----YVQ----------RGDQASIVG------GAIE-----------FVK------------E-L--------EHL-----------LQ
GhbHLH407   EAETQRMTHIAVE--RNRRK-------QMNEH---------LAVLRS-LMPES-----YVQ----------RGDQASIVG------GAIE-----------FVK------------E-L--------EHL-----------LQ
GrbHLH142   EAETQRMTHIAVE--RNRRK-------QMNEH---------LAVLRS-LMPES-----YVQ----------RGDQASIVG------GAIE-----------FVK------------E-L--------EHL-----------LQ
GabHLH061   EAETQRMTHIAVE--RNRRK-------QMNEH---------LAVLRS-LMPES-----YVQ----------RGDQASIVG------GAIE-----------FVK------------E-L--------EHL-----------LQ
TcbHLH048   EAETQRMTHIAVE--RNRRK-------QMNEH---------LAVLRS-LMPES-----YVQ----------RGDQASIVG------GAIE-----------FVK------------E-L--------EHL-----------LQ
OsbHLH050   DAESQRMTHIAVE--RNRRR-------QMNEY---------LAVLRS-LMPES-----YVH----------RGDQASIVG------GAID-----------FVK------------E-L--------EQL-----------LQ
OsbHLH049   ETETQRMTHIAVE--RNRRR-------QMNEY---------LAILRS-LMPEP-----YVQ----------RGDQASIVG------GAIE-----------FVK------------E-L--------EQQ-----------LQ
GhbHLH130   EVESQRMTHIAVE--RNRRK-------QMNEH---------LRVLRS-LMPGS-----YVQ----------RGDQASIIG------GAIE-----------FVR------------E-L--------EQL-----------LQ
GhbHLH346   EVESQRMTHIAVE--RNRRK-------QMNEH---------LRVLRS-LMPGS-----YVQ----------RGDQASIIG------GAIE-----------FVR------------E-L--------EQL-----------LQ
GrbHLH198   EVESQRMTHIAVE--RNRRK-------QMNEH---------LRVLRS-LMPGS-----YVQ----------RGDQASIIG------GAIE-----------FVR------------E-L--------EQL-----------LQ
At097FMA    EVESQRMTHIAVE--RNRRK-------QMNEH---------LRVLRS-LMPGS-----YVQ----------RGDQASIIG------GAIE-----------FVR------------E-L--------EQL-----------LQ
TcbHLH059   EVESQRMTHIAVE--RNRRK-------QMNEH---------LRVLRS-LMPGS-----YVQ----------RGDQASIIG------GAIE-----------FVR------------E-L--------EQL-----------LQ
VvbHLH064   EVESQRMTHIAVE--RNRRK-------QMNEH---------LRVLRS-LMPSS-----YVQ----------RGDQASIIG------GAIE-----------FVR------------E-L--------EQL-----------LQ
OsbHLH051   EVESQRMTHIAVE--RNRRR-------QMNEY---------LRVLRS-LMPGS-----YVQ----------RGDQASIIA------GAIE-----------FIR------------E-L--------EQL-----------IQ
GhbHLH149   EVESQRMTHIAVE--RNRRR-------QMNDY---------LNSLRS-LMPPC-----YIQ----------RGDQASIIA------GAID-----------FVK------------E-L--------EQL-----------LQ
GhbHLH369   EVESQRMTHIAVE--RNRRR-------QMNDY---------LNSLRS-LMPPC-----YIQ----------RGDQASIIA------GAID-----------FVK------------E-L--------EQL-----------LQ
GhbHLH391   EVESQRMTHIAVE--RNRRR-------QMNDY---------LNSLRS-LMPPC-----YIQ----------RGDQASIIA------GAID-----------FVK------------E-L--------EQL-----------LQ
GrbHLH102   EVESQRMTHIAVE--RNRRR-------QMNDY---------LNSLRS-LMPPC-----YIQ----------RGDQASIIA------GAID-----------FVK------------E-L--------EQL-----------LQ
GrbHLH103   EVESQRMTHIAVE--RNRRR-------QMNDY---------LNSLRS-LMPPC-----YIQ----------RGDQASIIA------GAID-----------FVK------------E-L--------EQL-----------LQ
GabHLH034   EVESQRMTHIAVE--RNRRR-------QMNDY---------LNSLRS-LMPPC-----YIQ----------RGDQASIIA------GAID-----------FVK------------E-L--------EQL-----------LQ
GabHLH033   EVESQRMTHIAVE--RNRRR-------QMNDY---------LNSLRS-LMPPC-----YIQ----------RGDQASIIA------GAID-----------FVK------------E-L--------EQF-----------LQ
TcbHLH017   EVESQRMTHIAVE--RNRRR-------QMNDY---------LNSLRS-LMPPS-----YIQ----------RGDQASIIG------GAID-----------FVK------------E-L--------EQL-----------LQ
VvbHLH079   EVESQRMTHIAVE--RNRRR-------QMNDH---------LNALRS-LMPTS-----YIQ----------RGDQASIIG------GAID-----------FVK------------E-L--------EQL-----------LE
At057       EVENQRMTHIAVE--RNRRR-------QMNEH---------LNSLRS-LMPPS-----FLQ----------RGDQASIVG------GAID-----------FIK------------E-L--------EQL-----------LQ
At070       EIESQRMTHIAVE--RNRRR-------QMNVH---------LNSLRS-IIPSS-----YIQ----------RGDQASIVG------GAID-----------FVK------------I-L--------EQQ-----------LQ
At071       EAENQRMTHIAVE--RNRRR-------QMNQH---------LSVLRS-LMPQP-----FAH----------KGDQASIVG------GAID-----------FIK------------E-L--------EHK-----------LL
OsbHLH052   EAENQRMTHIAVE--RNRRR-------LMNDH---------LASLRS-LIPSN-----YIP----------RGDQATVVG------GAID-----------YVK------------Q-L--------EQQ-----------LV
At067       EIENQRINHIAVE--RNRRR-------QMNEH---------INSLRA-LLPPS-----YIQ----------RGDQASIVG------GAIN-----------YVK------------V-L--------EQI-----------IQ
At099       DKENQRMNHIAVE--RNRRK-------QMNHF---------LSILKS-MMPLS-----YSQ----------PNDQASIIE------GTIS-----------YLK------------K-L--------EQR-----------LQ
GhbHLH111   ------MSHIAVE--RNRRR-------QMNEH---------LKVLRS-LTPCF-----YIK----------RGDQASVIG------GVIE-----------FIK------------E-L--------QQV-----------LQ
GrbHLH072   ------MSHIAVE--RNRRR-------QMNEH---------LKVLRS-LTPCF-----YIK----------RGDQASVIG------GVIE-----------FIK------------E-L--------QQV-----------LQ
```

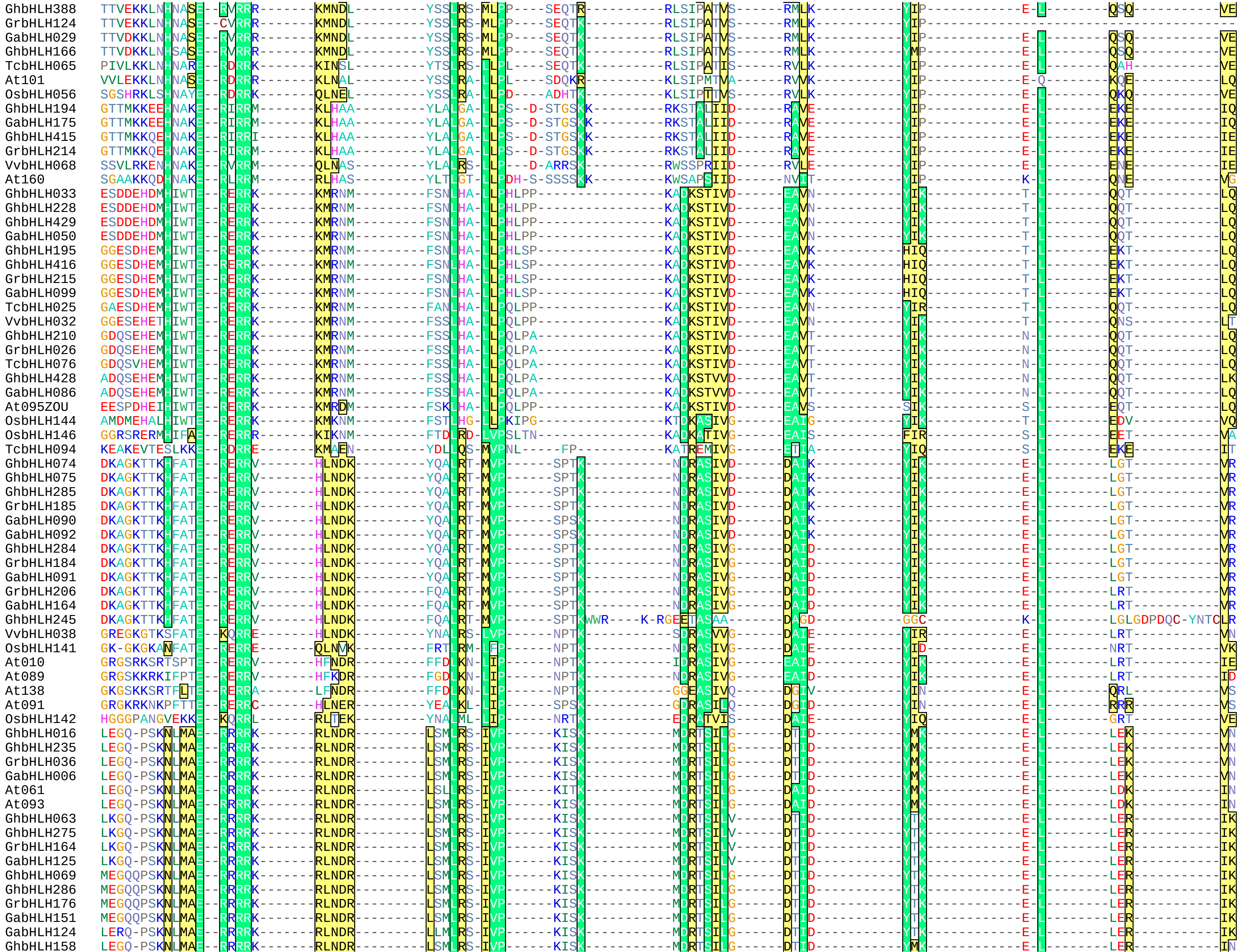
GhbHLH388
GrbHLH124
GabHLH029
GhbHLH166
TcbHLH065
At101
OsbHLH056
GhbHLH194
GabHLH175
GhbHLH415
GrbHLH214
VvbHLH068
At160
GhbHLH033
GhbHLH228
GhbHLH429
GabHLH050
GhbHLH195
GhbHLH416
GrbHLH215
GabHLH099
TcbHLH025
VvbHLH032
GhbHLH210
GrbHLH026
TcbHLH076
GhbHLH428
GabHLH086
At095ZOU
OsbHLH144
OsbHLH146
TcbHLH094
GhbHLH074
GhbHLH075
GhbHLH285
GrbHLH185
GabHLH090
GabHLH092
GhbHLH284
GrbHLH184
GabHLH091
GrbHLH206
GabHLH164
GhbHLH245
VvbHLH038
OsbHLH141
At010
At089
At138
At091
OsbHLH142
GhbHLH016
GhbHLH235
GrbHLH036
GabHLH006
At061
At093
GhbHLH063
GhbHLH275
GrbHLH164
GabHLH125
GhbHLH069
GhbHLH286
GrbHLH176
GabHLH151
GabHLH124
GhbHLH158

```
GhbHLH322  RIETPLP----LPTFKVRKE-------KLGDR---------ITALQQ-LVSP------FGK-----------TDTASMLH------EIIE-----------HIK------------F-L--------HDQ-----------MN
OsbHLH068  RLETPSP----LPTFKVRKE-------KLGDR---------ITALQQ-LVSP------FGK-----------TDTASMLH------EIIE-----------YIK------------F-L--------HDQ-----------MG
GhbHLH282  KLESPSP----LPTFKVRKE-------KLGDR---------ITALQQ-LVSP------FGK-----------TDTASMLH------EAIE-----------YIK------------F-L--------HDQ-----------MN
GrbHLH182  KLESPSP----LPTFKVRKE-------KLGDR---------ITALQQ-LVSP------FGK-----------TDTASMLH------EAIE-----------YIK------------F-L--------HDQ-----------MN
GabHLH102  KLESPSP----LPTFKVRKE-------KLGDR---------ITALQQ-LVSP------FGK-----------TDTASMLH------EAIE-----------YIK------------F-L--------HDQ-----------MN
OsbHLH067  RIEAPSP----MPTFKVRKE-------KLGDR---------ITALQQ-LVSP------FGK-----------TDTASMLH------EAIE-----------YIK------------F-L--------HDQ-----------MA
At112      RVTTPSP----LPTFKVRKE-------NLRDQ---------ITSLQQ-LVSP------FGK-----------TDTASMLQ------EAIE-----------YIK------------F-L--------HDQ-----------MT
At103      RLETPSH----FPSFKVRKE-------KLGDR---------ITALQQ-LVSP------FGK-----------TDTASMLH------DAID-----------YIK------------F-L--------QEQ-----------IT
At114      RLETLSP----LPSFKVRKE-------KLGDR---------ITALQQ-LVSP------FGK-----------TDTASMLN------EAVE-----------YIK------------F-L--------QEQ-----------MT
GhbHLH064  RLESRAS----CPPFKVRKE-------KLGDR---------IAALQQ-LVAP------FGK-----------TDTASMLM------EAIG-----------YIK------------F-L--------QNQ-----------VE
GrbHLH160  RLESRAS----CPPFKVRKE-------KLGDR---------IAALQQ-LVAP------FGK-----------TDTASMLM------EAIG-----------YIK------------F-L--------QNQ-----------VE
GabHLH145  RLESRAS----CPPFKVRKE-------KLGDR---------IAALQQ-LVAP------FGK-----------TDTASMLM------EAIG-----------YIK------------F-L--------QNQ-----------VE
GhbHLH276  RLESRA-----CPPFKVRKE-------KLGDR---------IAALQH-LVAP------FGK-----------TDTASMLM------EAIG-----------YIK------------F-L--------QNQ-----------VE
GrbHLH172  RLESRA-----CPPFKVRKE-------KLGDR---------IAALQH-LVAP------FGK-----------TDTASMLM------EAIG-----------YIK------------F-L--------QNQ-----------VE
GhbHLH263  RLESPAS----CPPFKVRKE-------KLGDR---------IAALQQ-LVAP------FGK-----------TDTASMLM------EAIG-----------YIK------------F-L--------QNQ-----------VE
GhbHLH066  RLESRA-----CPLFKVRKE-------KLGDR---------IAALQH-LVAP------YGK-----------TDTASMLM------EAIG-----------YIK------------F-L--------QNQ-----------VE
GabHLH168  RLESRA-----CPPFKVRKE-------KLGDR---------IAALQH-LVAP------YGK-----------TDTASMLM------EAIG-----------YIK------------F-L--------QNQ-----------VE
OsbHLH071  RLESHSSSM-LPSFKVRKE-------KLGDR---------IAALQQ-LVSP------FGK-----------TDTASMLM------EAIG-----------YIK------------F-L--------QDQ-----------VE
OsbHLH069  SCSSNAN----TLLAKVRKE-------KLGDR---------ITALQQ-LVSP------FGK-----------TDTASMLH------EAIE-----------YIK------------F-L--------HEQ-----------MA
OsbHLH070  QMVIDFL----PIKFHVRKE-------KLGDR---------VTALQQ-LVSP------FGK-----------TDTASMLH------EIIE-----------YIK------------F-L--------HDQ-----------MG
GhbHLH020  RSSTARSP---CPTLKVRKE-------KLGDR---------VAALQK-LVAP------FGK-----------TDTASMLT------EAIG-----------YIQ------------F-L--------HDQ-----------VE
GhbHLH231  RSSTARSP---CPTLKVRKE-------KLGDR---------VAALQK-LVAP------FGK-----------TDTASMLT------EAIG-----------YIQ------------F-L--------HDQ-----------VE
GrbHLH032  RSSTARSP---CPTLKVRKE-------KLGDR---------VAALQK-LVAP------FGK-----------TDTASMLT------EAIG-----------YIQ------------F-L--------HDQ-----------VE
GabHLH214  RSSTARSP---CPTLKVRKE-------KLGDR---------VAALQK-LVAP------FGK-----------TDTASMLT------EAIG-----------YIQ------------F-L--------HDQ-----------VE
GhbHLH065  RVQPS-AT---QSTLKVRKE-------KLGDR---------ITSLHQ-LVSP------FGK-----------TDTASMLS------EAIG-----------YIR------------F-L--------QSQ-----------IE
GhbHLH252  RVQPS-AT---QSTLKVRKE-------KLGDR---------ITSLHQ-LVSP------FGK-----------TDTASMLS------EAIG-----------YIR------------F-L--------QSQ-----------IE
GrbHLH149  RVQPS-AT---QSTLKVRKE-------KLGDR---------ITSLHQ-LVSP------FGK-----------TDTASMLS------EAIG-----------YIR------------F-L--------QSQ-----------IE
GabHLH147  RVQPS-AT---QSTLKVRKE-------KLGDR---------ITSLHQ-LVSP------FGK-----------TDTASMLS------EAIG-----------YIR------------F-L--------QSQ-----------IE
GhbHLH070  RVQPS-AT---QSTFKVRKE-------KLGDR---------ITALHQ-LVSP------FGK-----------TDTASMLL------EAIG-----------YIR------------F-L--------QSQ-----------IE
GhbHLH079  RVQPS-AT---QSTFKVRKE-------KLGDR---------ITALHQ-LVSP------FGK-----------TDTASMLL------EAIG-----------YIR------------F-L--------QSQ-----------IE
GhbHLH280  RVQPS-AT---QSTFKVRKE-------KLGDR---------ITALHQ-LVSP------FGK-----------TDTASMLL------EAIG-----------YIR------------F-L--------QSQ-----------IE
GhbHLH287  RVQPS-AT---QSTFKVRKE-------KLGDR---------ITALHQ-LVSP------FGK-----------TDTASMLL------EAIG-----------YIR------------F-L--------QSQ-----------IE
GrbHLH001  RVQPS-AT---QSTFKVRKE-------KLGDR---------ITALHQ-LVSP------FGK-----------TDTASMLL------EAIG-----------YIR------------F-L--------QSQ-----------IE
GrbHLH178  RVQPS-AT---QSTFKVRKE-------KLGDR---------ITALHQ-LVSP------FGK-----------TDTASMLL------EAIG-----------YIR------------F-L--------QSQ-----------IE
GabHLH203  RVQPS-AT---QSTFKVRKE-------KLGDR---------ITALHQ-LVSP------FGK-----------TDTASMLL------EAIG-----------YIR------------F-L--------QSQ-----------IE
GabHLH095  RVQPS-AT---QSTFKVRKE-------KLGDR---------ITALHQ-LVSP------FGK-----------TDTASMLL------EAIG-----------YIR------------F-L--------QSQ-----------IE
At068      RLQPSPSS---QSTLKVRKE-------KLGGR---------IAALHQ-LVSP------FGK-----------TDTASMLS------EAIG-----------YIR------------F-L--------QSQ-----------IE
GhbHLH127  RVQTS-SS---QPPLKVRKE-------KLGDR---------ITALHQ-LVSP------FGK-----------TDTASMLL------EAIG-----------YIR------------F-L--------QSQ-----------IE
GhbHLH340  RVQTS-SS---QPPLKVRKE-------KLGDR---------ITALHQ-LVSP------FGK-----------TDTASMLL------EAIG-----------YIR------------F-L--------QSQ-----------IE
GrbHLH191  RVQTS-SS---QPPLKVRKE-------KLGDR---------ITALHQ-LVSP------FGK-----------TDTASMLL------EAIG-----------YIR------------F-L--------QSQ-----------IE
GabHLH080  RVQTS-SS---QPPLKVRKE-------KLGDR---------ITALHQ-LVSP------FGK-----------TDTASMLL------EAIG-----------YIR------------F-L--------QSQ-----------IE
OsbHLH073  RVQASSSA---QSTLKVRKE-------RLGDR---------ITALHQ-IVSP------FGK-----------TDTASMLQ------EIIG-----------YIR------------F-L--------LSQ-----------IE
OsbHLH074  RVQASSSA---QSTLKVRKE-------RLGDR---------ITALHQ-IVSP------FGK-----------TDTASMLQ------EIIG-----------YIR------------F-L--------LGQ-----------IE
OsbHLH072  RTQEPSPA---QATVKVRKE-------KLGDR---------ITALHQ-LVSP------FGK-----------TDTASMLL------EAIG-----------YIR------------F-L--------HGQ-----------IE
GhbHLH029  KLENSA-----SSSVKMHAPKV-----KLGDR---------ITALQQ-IVSP------FGK-----------TDTASMLL------EAIG-----------YIN------------F-L--------QEQ-----------VQ
GhbHLH223  KLENSA-----SSSVKMHAPKV-----KLGDR---------ITALQQ-IVSP------FGK-----------TDTASMLL------EAIG-----------YIN------------F-L--------QEQ-----------VQ
GrbHLH062  KLENSA-----SSSVKMHAPKV-----KLGDR---------ITALQQ-IVSP------FGK-----------TDTASMLL------EAIG-----------YIN------------F-L--------QEQ-----------VQ
OsbHLH075  KSETSHS----TSSPKHQSPKV-----KLGEK---------ITALQQ-IVSP------FGK-----------TDTASMLL------EIIT-----------YIK------------F-L--------HEQ-----------IQ
OsbHLH076  KQEASK-----ASPPKQPVPKV-----KLGEK---------ITALQQ-IVSP------FGK-----------TDTASMLF------EIIK-----------YIK------------F-L--------HEQ-----------VQ
OsbHLH077  KHEATSP----TSSLKSQVPKV-----KLGDK---------ITALQQ-IVSP------FGK-----------TDTASMLY------EAIN-----------YIK------------W-L--------HEQ-----------VQ
GhbHLH009  SLASKRQ-----KATKEKKD-------QVSEQ---------IAALQQ-LVSP------YGK-----------TDTASMLL------EAKG-----------YIR------------F-L--------HEQ-----------VK
GabHLH046  SLASKRQ-----KATKEKKD-------QVSER---------IAALQQ-LVSP------YGK-----------TDTASMLL------EAKG-----------YIR------------F-L--------HEQ-----------VK
GhbHLH215  SLASKRQ-----KATKEKKD-------QVSER---------IAALQQ-LVSP------YGK-----------TDTASMLL------EAMG-----------YIR------------F-L--------HEQ-----------VK
GrbHLH055  SLASKRQ-----KATKEKKD-------QVSER---------IAALQQ-LVSP------YGK-----------TDTASMLL------EAMG-----------YIR------------F-L--------HEQ-----------VK
GhbHLH343  RQKSDLS-----ISTKDRKD-------KVGER---------IVTLQQ-LVSP------YGK-----------TDTASMLL------QAME-----------YIQ------------F-L--------HEQ-----------VK
GrbHLH195  RQKSDLS-----ISTKDRKD-------KVGER---------IVTLQQ-LVSP------YGK-----------TDTASMLL------QAME-----------YIQ------------F-L--------HEQ-----------VK
GabHLH113  RQKSDLS-----ISTKDRKD-------KVGER---------IVTLQQ-LVSP------YGK-----------TDTASMLL------QAME-----------YIQ------------F-L--------HEQ-----------VK
GhbHLH133  RQKSDLL-----ISTKDRKD-------KVGER---------IVTLQQ-LVSP------YGK-----------TDTASMLL------QAME-----------YIQ------------F-L--------HEQ-----------VK
At153      -HKSDLS-----FSSKERKD-------KVGER---------ISALQQ-IVSP------YGK-----------TDTASMLL------DAMH-----------YIE------------F-L--------HEQ-----------VK
At154ERP   HKSSDLS-----FSSKERKD-------KLAER---------ISALQQ-LVSP------YGK-----------TDTASMLL------EGMQ-----------YIQ------------F-L--------QEQ-----------VK
OsbHLH083  TSPRDAP-----VSPKEKKD-------KIGER---------VAALQQ-LVSP------FGK-----------TDTASMLQ------EASG-----------YIK------------F-L--------HQQ-----------LE
GhbHLH196  KVENPTTG---GHAKVVRKE-------KIGDR---------INALQQ-LVSP------FGK-----------TDTASMLH------EAMG-----------YIR------------F-L--------HDQ-----------VQ
GrbHLH216  KVENPTTG---GHAK-VRKE-------KIGDR---------INALQQ-LVSP------FGK-----------TDTASMLH------EAMG-----------YIR------------F-L--------HDQ-----------VQ
GabHLH096  KVENPTTG---GHAK-VRKE-------KIGDR---------INALQQ-LVSP------FGK-----------TDTASMLH------EAMG-----------YIR------------F-L--------HDQ-----------VQ
GhbHLH425  KVENPTTS---GHAKVVRKE-------KIGDR---------INALQQ-LVSP------FGK-----------TDTASMLH------EAMG-----------YIR------------F-L--------HDQ-----------VQ
OsbHLH066  PAAATTTN---KRPR-VRRE-------RLGER---------IIALQQ-LVSP------FGK-----------SDTASMLH------EALG-----------YIR------------F-L--------HDQ-----------VQ
OsbHLH078  KPRNQTT--K-ATCK-KRSQ-------KLGDR---------ITALQQ-LVSP------YGK-----------TDTASMLH------EAAA-----------CIR------------Q-L--------HQQ-----------IQ
```

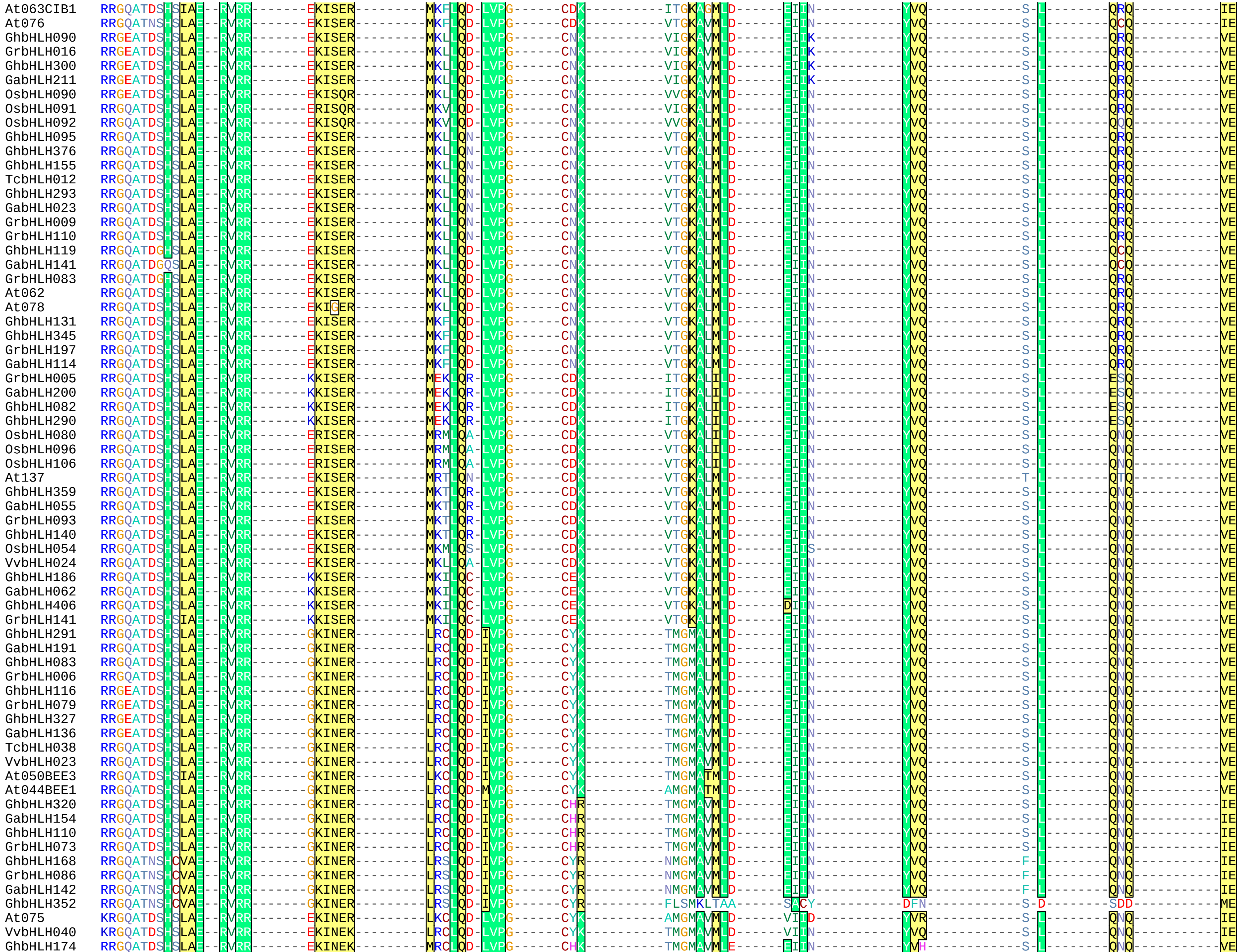
At063CIB1
At076
GhbHLH090
GrbHLH016
GhbHLH300
GabHLH211
OsbHLH090
OsbHLH091
OsbHLH092
GhbHLH095
GhbHLH376
GhbHLH155
TcbHLH012
GhbHLH293
GabHLH023
GrbHLH009
GrbHLH110
GhbHLH119
GabHLH141
GrbHLH083
At062
At078
GhbHLH131
GhbHLH345
GrbHLH197
GabHLH114
GrbHLH005
GabHLH200
GhbHLH082
GhbHLH290
OsbHLH080
OsbHLH096
OsbHLH106
At137
GhbHLH359
GabHLH055
GrbHLH093
GhbHLH140
OsbHLH054
VvbHLH024
GhbHLH186
GabHLH062
GhbHLH406
GrbHLH141
GhbHLH291
GabHLH191
GhbHLH083
GrbHLH006
GhbHLH116
GrbHLH079
GhbHLH327
GabHLH136
TcbHLH038
VvbHLH023
At050BEE3
At044BEE1
GhbHLH320
GabHLH154
GhbHLH110
GrbHLH073
GhbHLH168
GrbHLH086
GabHLH142
GhbHLH352
At075
VvbHLH040
GhbHLH174

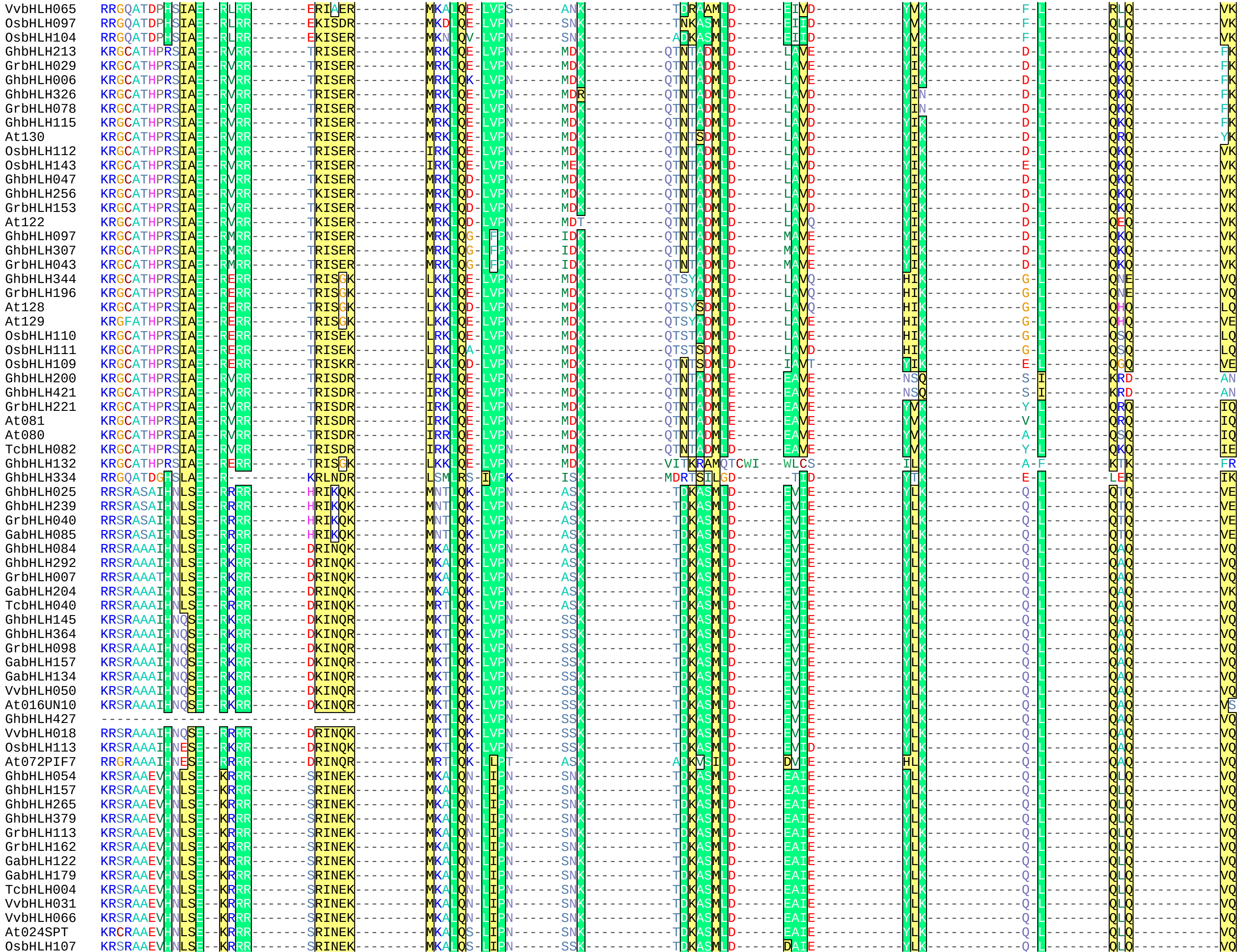

VvbHLH065
OsbHLH097
OsbHLH104
GhbHLH213
GrbHLH029
GhbHLH006
GhbHLH326
GrbHLH078
GhbHLH115
At130
OsbHLH112
OsbHLH143
GhbHLH047
GhbHLH256
GrbHLH153
At122
GhbHLH097
GhbHLH307
GrbHLH043
GhbHLH344
GrbHLH196
At128
At129
OsbHLH110
OsbHLH111
OsbHLH109
GhbHLH200
GhbHLH421
GrbHLH221
At081
At080
TcbHLH082
GhbHLH132
GhbHLH334
GhbHLH025
GhbHLH239
GrbHLH040
GabHLH085
GhbHLH084
GhbHLH292
GrbHLH007
GabHLH204
TcbHLH040
GhbHLH145
GhbHLH364
GrbHLH098
GabHLH157
GabHLH134
VvbHLH050
At016UN10
GhbHLH427
VvbHLH018
OsbHLH113
At072PIF7
GhbHLH054
GhbHLH157
GhbHLH265
GhbHLH379
GrbHLH113
GrbHLH162
GabHLH122
GabHLH179
TcbHLH004
VvbHLH031
VvbHLH066
At024SPT
OsbHLH107

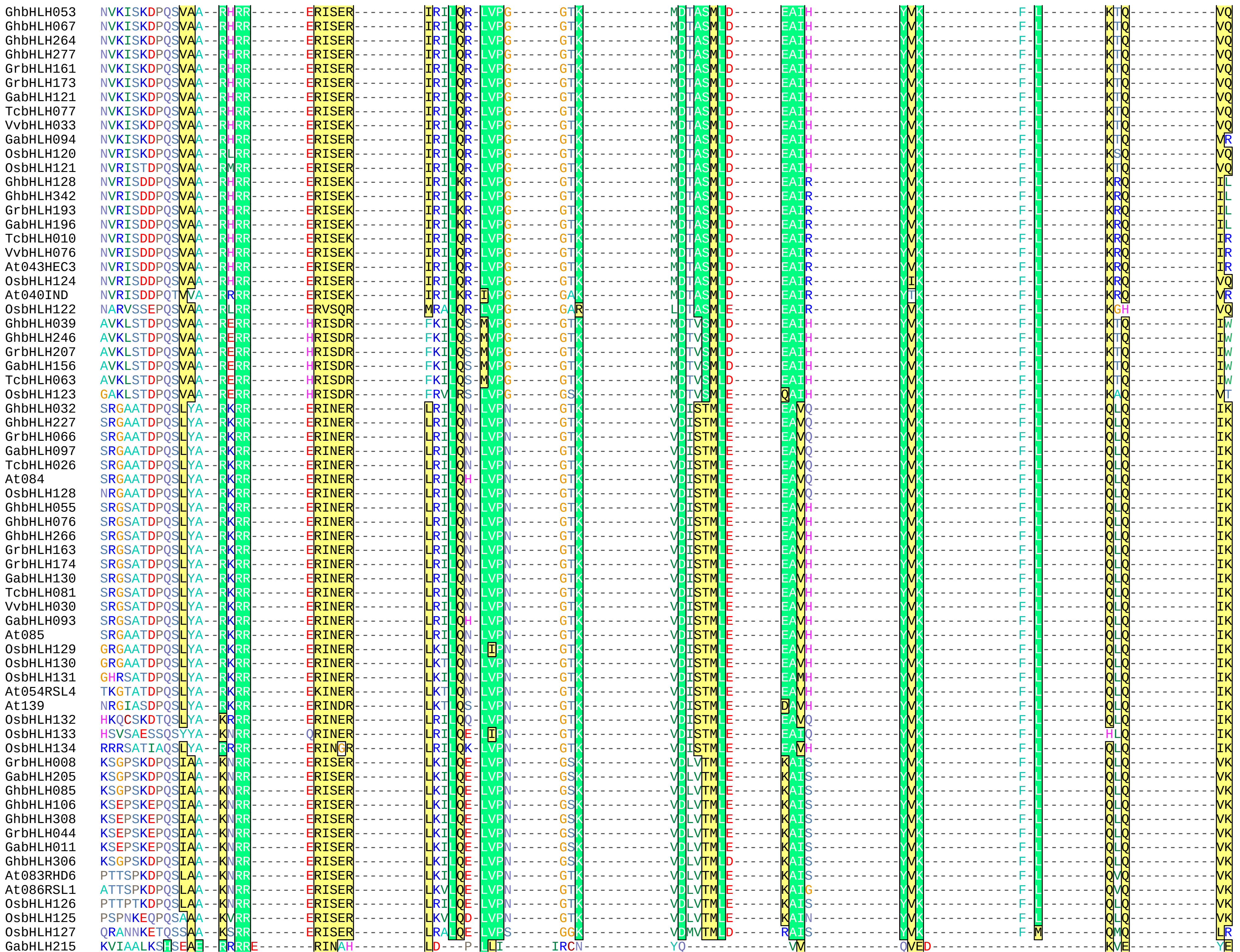
GhbHLH053
GhbHLH067
GhbHLH264
GhbHLH277
GrbHLH161
GrbHLH173
GabHLH121
TcbHLH077
VvbHLH033
GabHLH094
OsbHLH120
OsbHLH121
GhbHLH128
GhbHLH342
GrbHLH193
GabHLH196
TcbHLH010
VvbHLH076
At043HEC3
OsbHLH124
At040IND
OsbHLH122
GhbHLH039
GhbHLH246
GrbHLH207
GabHLH156
TcbHLH063
OsbHLH123
GhbHLH032
GhbHLH227
GrbHLH066
GabHLH097
TcbHLH026
At084
OsbHLH128
GhbHLH055
GhbHLH076
GhbHLH266
GrbHLH163
GrbHLH174
GabHLH130
TcbHLH081
VvbHLH030
GabHLH093
At085
OsbHLH129
OsbHLH130
OsbHLH131
At054RSL4
At139
OsbHLH132
OsbHLH133
OsbHLH134
GrbHLH008
GabHLH205
GhbHLH085
GhbHLH106
GhbHLH308
GrbHLH044
GabHLH011
GhbHLH306
At083RHD6
At086RSL1
OsbHLH126
OsbHLH125
OsbHLH127
GabHLH215

```
OsbHLH178   ----PPPPAARPA--PRSR-------PPLASK---------AKVLGR-LVPGCRK-LAFPA----------------LLA------EASD-----------YIA-------------A-L--------EMQ-----------MR
OsbHLH139   -SSSYGGGQARPG---RKK-------ERMKKM---------VRTLKG-IIPGGNQ-MDTPA----------------VLD------EAVR-----------YLK-------------S-L--------KVE-----------VK
OsbHLH140   -G---GGRKPEAGGGERKK-------ERMKKM---------MRTLKG-IIPGGDR-MDTPA----------------VLD------EAVR-----------YLK-------------S-L--------KVE-----------VK
At168P1r3   -MRTLKTQTT----RGRR--------RANV-----------SSRTR-VLHT-----CCGN------------GS---------------------------------------------------------------------
At169       -MRILKTQRS-----RGGR--------R--------------TSKK------------FGN------------RR---------------------------------------------------------------------
GhbHLH269   ---PGPISKDVFDDNE----------TDVQSE--------MHED-TE-ELN------ALLY-----------SDDDSDYTE----DEEVT---------------------------S-T--------GH--------SPSTMT
GrbHLH167   ---PGPISKDVFDDNE----------TDVQSE--------MHED-TE-ELN------ALLY-----------SDDDSDYTE----DEEVT---------------------------S-T--------GH--------SPSTMT
GhbHLH058   ---PGPISKDVFDDNE----------TNVQSE--------MHED-TE-ELN------ALLY-----------SDDDSDYTE----DEEVT---------------------------S-T--------GH--------SPSTMT
GhbHLH279   ---SGPISSNVFDENG----------TNTQSE--------MHED-TE-ELN------ALLY-----------SDDDSYYT-----DDEVT---------------------------S-A--------VH--------SPSRMT
GrbHLH177   ---SGPISSNVFDENG----------TNTQSE--------MHED-TE-ELN------ALLY-----------SDDDSYYT-----DDEVT---------------------------S-A--------VH--------SPSRMT
GhbHLH077   ---SGLISSNVYDENG----------TNTQSE--------MHED-TE-ELN------ALLY-----------SDDDSYYT-----DDEVT---------------------------S-A--------VH--------SPCTMT
At142SAC5   -SELQGIEKAFKEDGEEFHK-----SDGTESE--------MHED-TE-EIN------ALLY-----------SDDD    Y----DDDCE---------------------------S--------------------------
At143       -SKDHAIQEKILQHED--HE-----NGEEDSE--------MHED-TE-EIN------ALLY-----------SDDD    D----NDDWE---------------------------S--------------------------
At034       ----RVVNQLRGEAHE---------LQETNQK--------LLEE-IK-SLK------ADKN----------ELREEKLVL-----KAEKE---------------------------K-M--------EQ-----------QLK
At104       ----RILNQLRDEALK---------LEETNQK--------LLEE-IK-SLK------AEKN----------ELREEKLVL-----KADKE---------------------------K-T--------EQ-----------QLK
At105ILR3   ----RMVTQLRGEAQK---------LKDSNSS--------LQDK-IK-ELK------TEKN----------ELRDEKQRL-----KTEKE---------------------------K-L--------EQ-----------QLK
At115       ----RMVNQARDEAQK---------LKDLNSS--------LQEK-IK-ELK------DEKN----------ELRDEKQKL-----KVEKE---------------------------R-I--------DQ-----------QLK
At146       ----FSLEPNKNPSAVF--------TRKYTSH--------LVPA-LK-KLN------MNKN----------SSK-----------QTVKH---------------------------E-V--------DM-----------AL-
At170       ----FSLQPNKNPKAVF--------ARRYVSH--------LVPA-LK-KIN------MNKS----------SSKTNKQSLE----QTVKH---------------------------E-V--------DM-----------AF-
TcbHLH067   -YGTGYQYGSWNDSAH---------LTKNFSG--------LKRA-QD-NDR------KFF----------STNQNGDLGN-----CVHVL---------------------------S-H--------HL-----------SLP
TcbHLH086   -YSSGFPVTSWEDSMM---------ISDNMPG--------VKRL-RE-DDR------SLSGLDL---DGAETQNTDAGNR-----PPPIL---------------------------A-H--------HL-----------SLP
TcbHLH020   -GSNGHFMSNLGTDSW---------NNASLSG--------LKRA-RE-SDG------DLFR------NLSRSQTQDYRD------RSTAL---------------------------T-H--------HL-----------SLP
GabHLH088   -YTDDEVTSAVHSPCT---------MTAHPEQ--------LEGGGTE-SVA------SSIG----------PTKKRKLLDD----GNDYL---------------------------P-L--------LT----------DAAS
GabHLH188   -TEDEEVTSTGHSPST---------MTAHCEQ--------FEGG-AE-GVA------SSSG----------LTKKRKLLDG----SNDYT---------------------------P-L--------LM----------DNTS
At159P1r2   --------TSVHER-----------KKAIKLS----------ADVAM-AS---------LR-----------KGTTCWSR-----ALIEKT-------ATEDNFLVRQMLSG-----I-K--------AE-----------TLI
At167P1r1   --------LNVTER-----------KNAIRLS----------SDLAM-AA---------AR-----------NGSTVWSR-----ALISR--------------------SG-----N-K--------T---------------
At165PAR1   -----------EET-----------LATPDAT---------RRSLSP-SCS------ATVK-----------SRAAGFER-----RTKRRL--------------------------S-E--------TN-----------A--
At166PAR2   ----------MEKT-----------LAT-SHT---------KRSSPP-SPS------SAVN-----------TSSTGFNR-----RTRQRL--------------------------S-D--------AT-----------A--
At052       --SPSLFGFDHYDHF----------YESFLPS---------QEIFLP-SPKT-----RVFN----------ESQELDSFH-----TPKHQK--------------------------L-I--------DS-----------SF-
At053       --QEEAPSQTHFDPF----------CDQFLSP---------QEIFLP-NPKN-----EIFN----------ETHDLDFFLP----TPKRQR--------------------------L-V--------NS-----------SY-
GabHLH143   -LSPSSSSNRLKNQIS---------FSSRLPS---------SLGILS-QIS------EVVD---------D----AKLETG----   CQ---------------------------Y-G--------SW------------ND
GabHLH004   -LSPS--SNRLKNQIS---------FSSRQPS---------SLGILS-QIS------EIEN---------ENLGANSFDGGK--- PEYQ---------------------------C-G--------SW------------NE
GabHLH112   -------VSRLKSQLS---------FTR-QDS----------LSQIS-EVSE-----NLVD---------GVSSNSNHQNPA--- HSFA---------------------------A-A--------GF-----------GMD
VvbHLH042   -CNGGHGISRLKSQLS---------FKR-QDS----------LSQIS-EVSE-----NMVD---------GISSDNGHRNAT--- HSYA---------------------------T-A--------SF-----------PMD
At147       -PSPSISLEKWRSEKQQQIY-----STKLVHA---------LRELRI-SQQ------PSSS-----------SSSS- IPRG---GRAVR---------------------------E-V--------AD-----------RAL
At148       -SVSGEIHARWRSEKQQRIY-----SAKLFQA---------LQQVRL-NSS------ASTS-----------SSPT  AQKR---GKAVR---------------------------E-A--------AD-----------RAL
At149       -RVNEESLKRWKTNRVQQIY-----ACKLVEA---------LRRVRQ-RSS------TTSN-----------NETDKLVSGA---AREIR---------------------------D-T--------AD-----------RVL
OsbHLH160   --PEQLGSTSS-SMPMPRV------SVSSVEL---------EKKIGF-RIN------VSME-----------KSQPELLTSV---LEAFE---------------------------E-L--------GL-----------DVL
OsbHLH161   --PEQLGSTSS-SMPMPRV------SVSSVEL---------EKKRGF-RIN------VSME-----------KSQPELLTSV---LEAFE---------------------------E-L--------GL-----------DVL
OsbHLH163   ---KEIACAEAAALRQNSI------PTVTVET---------LGH-GF-LIN------VFSD-----------KSCPGLLVSI---LEAFD---------------------------E-L--------GL-----------NVL
OsbHLH166   RLNQEIACAQD-ALRQN---------RVTVET---------LRH-GF-LVN------VFSG-----------KSCPGLLVSI---LEAFD---------------------------E-L--------GL-----------SVL
OsbHLH162   ---KELGCAKNMNICEEPS------PVVRVQV---------LDK-GF-LIN------VFMD-----------KSSPGLLSSI---LQAFD---------------------------E-L--------GL-----------TVI
At108MEE8   KSSDKSDHDTLLK--KKRRE-------RIRRQ---------LETLKE-ITP-------NCP----------QSDINAILD------CVIE-----------YTNN-----------LR-L--------AHY-------------
TcbHLH085   SGGSRTSPIEVLEL-KVTH---------MGEK----------TVVVS-ITCS-------KR-----------TDTMVKLC------EVFE-----------SLK               L        KII
GhbHLH014   SQSIADKVFFYIQ--NRRE--------RIGER---------LKILQE-LVP------NGPKMMK---------QRHKMKREAN-- IMLK            LDGR-------RIRKL--------QRLIQS--------LQ
GabHLH042   -AGGCSVPEVLPATANSRD----MIGEIENRS-----T-HLLAIKT-DVETQG--DFIR------FLIKEVENAAFTD---IEDVVP           FVK              WL        DDE           LS
GabHLH162   CTIPAKLLLQLTS--AFRER-------GLCDRNGK------FFFKDH-LHKSN--VPVLA----------IAGDQDLICP----PEAVEE          TVK              LL        PRN----------LVT
VvbHLH073   NSGGFITGFPIGS-WDDSA--------IMSES---------FSSLKS-VRD--------DE-----------AKTFSGLN-----  ASE-----------AQKG------------EPA--------NRPP----------VL
GhbHLH178   ---IENTKTATDN--DHDS---------ASHR----------AELQL-SITN--------------------------LN-------VKE-----------EIR----------------------------------------
At011       SVQQHSSSSADAS-MKQDS--------KIKPL-----------DLDL-MMNS---------------------NHSGQGN-----DQKD------------DVRL-------------KL--------ELK-------------
OsbHLH064   EKNELRDEKVSLK-FEVDN---------LNTQY--------QQRMRV-LYP-------WTG-----------MEPSVVIG------PPLP-----------YPF--------------SV--------PVP-----------VP
At110       SMDDMSNISRPFD-INMQV---------FDGR---------LFEGNV-LVPPFN-AQEISSLG---------MSRGSLPS------FGLP-----------FHH--------------HL--------QQT-----------LP
GabHLH012   -NGEPTSSGRLYN--HISFS---------SAP---------MPQIAQ-VEN--------ED-----------LASNGNGR------NGMN-----------FIP--------------NL--------MTD-----------SR
At102BIM2   ESDKARAITGISI--ESQP--------ELDDKG--------LPPLQP-ILPMVQ----GEQ-----------ANECPATS------DGLG-----------QSN-------------DLV--------IEG------------G
At121       APCPTYMPYMPPNTVVEQQS------VHIPQN----------PGNRS-REPR---AKVSR-----------ESRSEKAE------DSNE-----------VAT--------------QL--------ELK-------------
At144       NYGNYEQEVSSSY--QEDPN-------EIDAL---------LSADED-YEEN-----DDNE-----------GEEDGGDS------EEVS-----------TAR---------------T--------SSRD----------YG
At150       LMEALRRSRVSSE--EAPVR-------HLSRR---------WRATTA-QKVY------SLK-----------LYDALQRS------RRSA-----------TVR-------------DTA--------DK------------VL
OsbHLH017   GMSGSDTNNTDTE--RNSG--------QLQLQ---------MQDQLN-MVSN-----DHQT-----------IPNNAVSS------ELMQC----------EMS-------------EVV--------RDG-----------CS
OsbHLH033   ICGKGFKRDANLR-MHMRG---------HGDE---------YKTAAA-LAKP------SKDS---------SLESAPVTR------YSCP-----------YVG---------------C--------KRNK----------EH
GhbHLH398   DNDHDSASHSKPQ--EETGE---------EDK---------KETMKS-SIS-------TKR------------SRAAAIH------NQSE-----------RVS-------------LFF--------AN------------PE
GabHLH021   SASRPPTPSGLMT--PIA---------EMGNKS--------MGPFSS-ENAG------FGE------------NRPSNYS------SGLP----------- VT--------------SW--------DDS-----------M-
At145       RFLVFDQSGDQTT-LLLAS--------DIRKS---------FETLKQHACPDMK--EELQR-----------SNKDLFVC------HGMQG----------NSEP-------------DL--------KED-------------
At155CPu    QSLLTNMELAEPS--GQKKH-------NIVNP---------INSAMN-QPPMA----EVDTQ----------QNSSDICG---AFSSIG------FSSTYPSSSSD--QFQTSLDI--------PKKNKKR-------AK
OsbHLH145   LQAPPPSEEENEE--HDSVV-------AAATR---------EMALAD-MVHA------WEQ-----------QQEAAATG------GSHGG----------HAV---------------P--------PPPP----------AA
At046BIM1   TATTSPFPLSIQS--NSFFS------PVIAGN--------PVPQFHARVASS-----EAVE-----------PSPSSRSQ------KEEE-----------DEE--------------VL--------EGN-------------
At157       FPAESFQGFSFDD--IFAED---------NPP----------SLLSPEMISS-----EAAS------------SNQDLTNG-----DDYG------------FD---------------I--------LQS-----------YS
OsbHLH155   TFTASYEQPPPAP--AAGFD-------CLSEVYG--------NAAAA-FGPN------AGG------------GGGEYGGG-----GDMG-----------FLD--------------VV--------EPK-----------AS
```
