# Supplementary figures and images for: Divergence and evolution of cotton bHLH proteins from diploid to allotetraploid
Source: BMC Genomics. 2018 Feb 23;19:162. doi: 10.1186/s12864-018-4543-y (PMC5824590; doi:10.1186/s12864-018-4543-y)

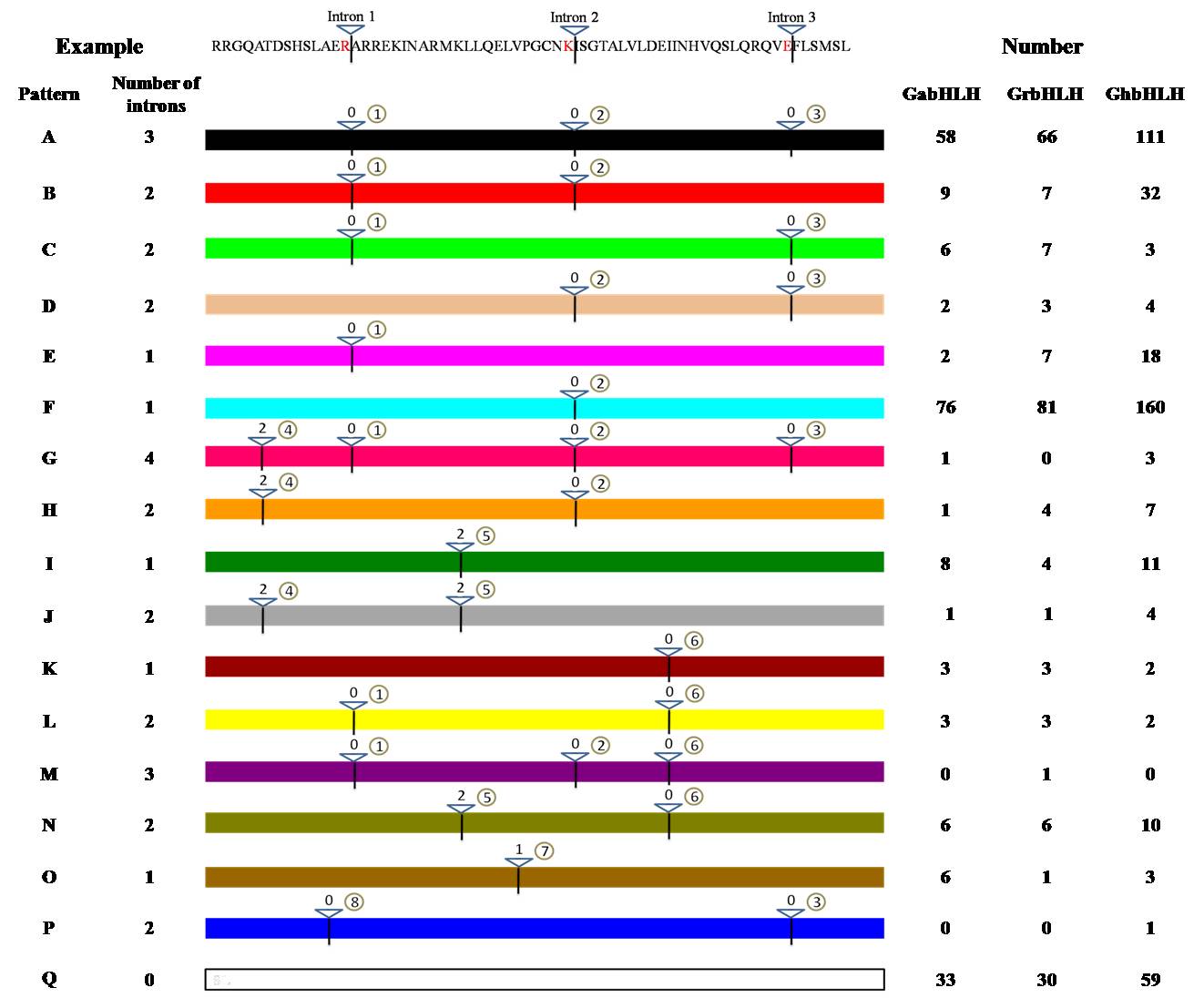

Supplement: Supplementary file 8 — Intron distribution within the bHLH domains of three cotton species. (JPEG 122 kb) [file 12864_2018_4543_MOESM8_ESM.jpg]

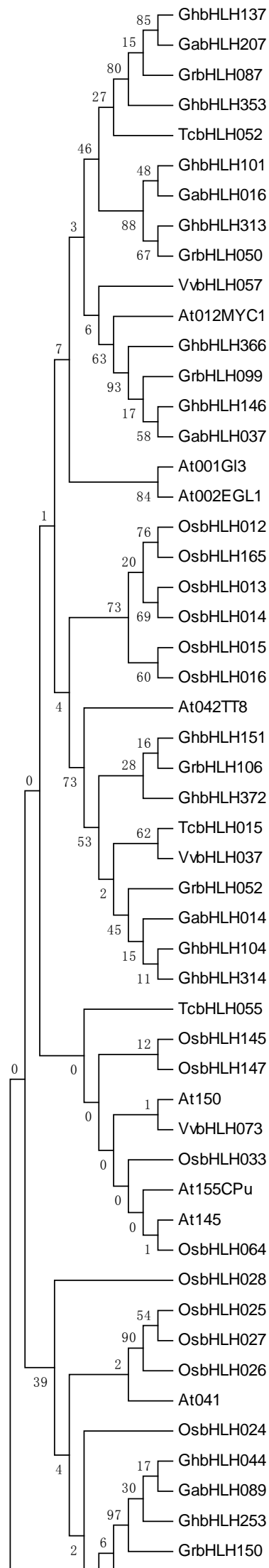

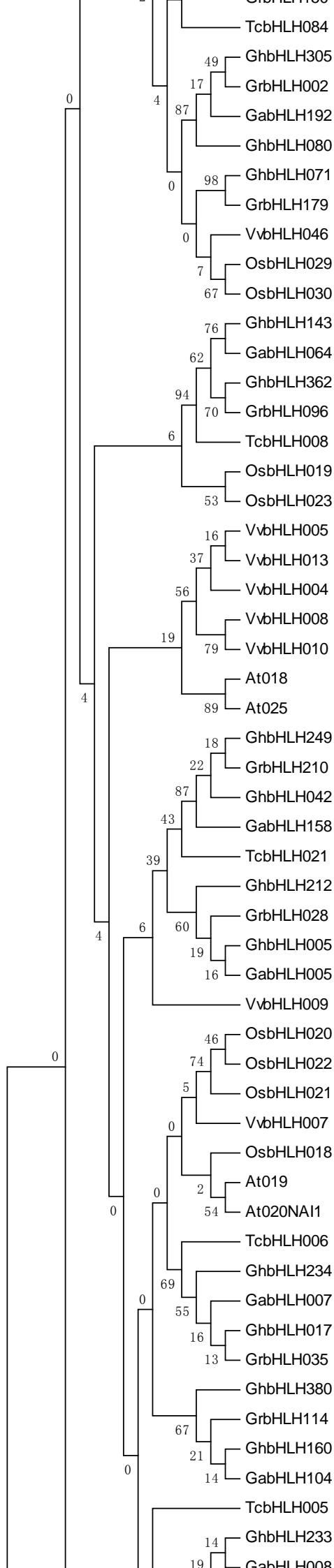

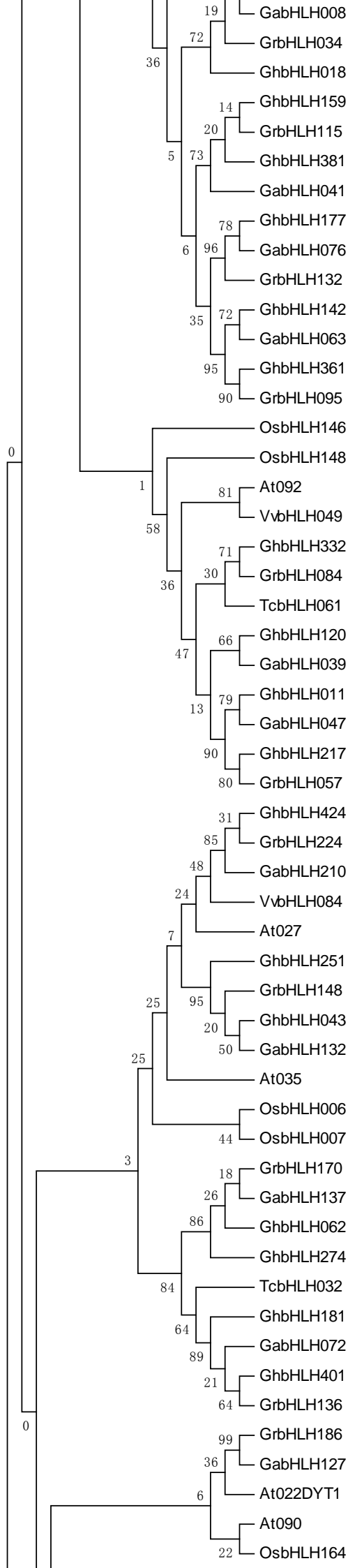

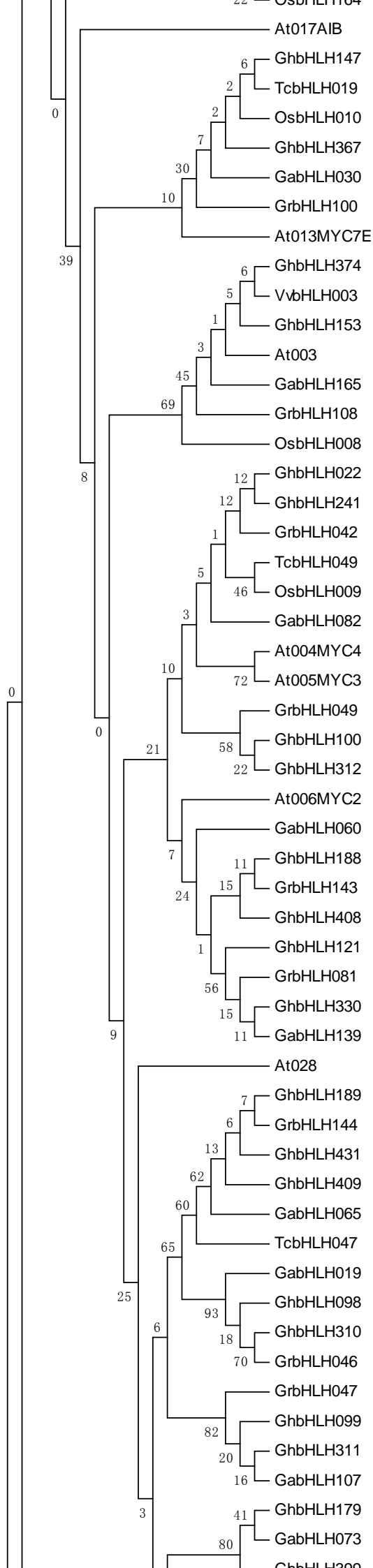

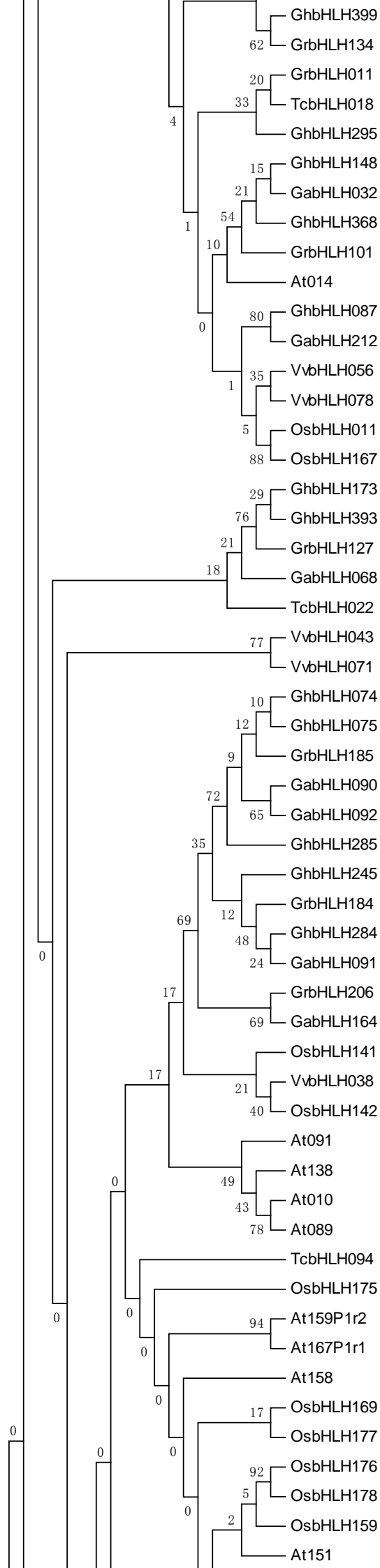

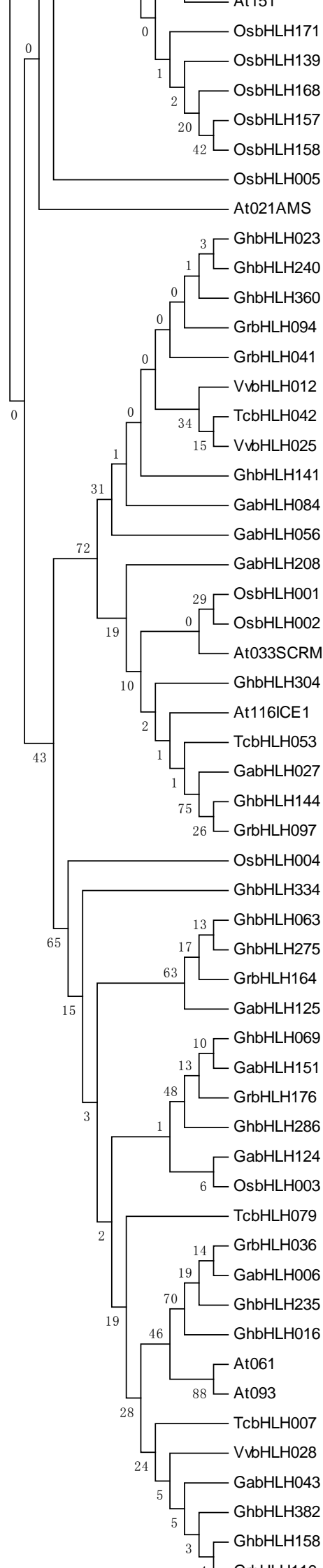

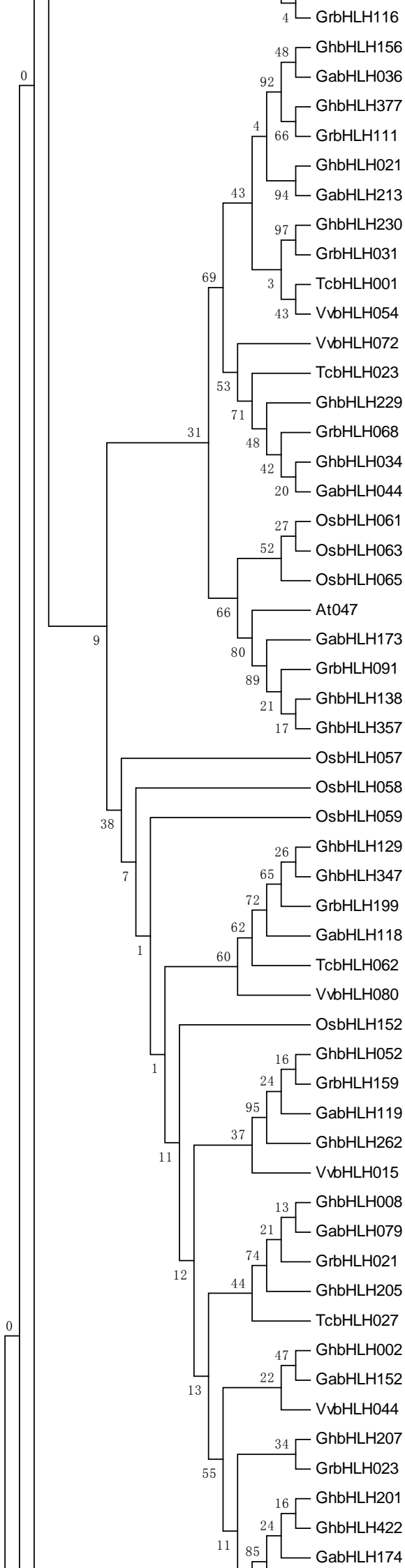

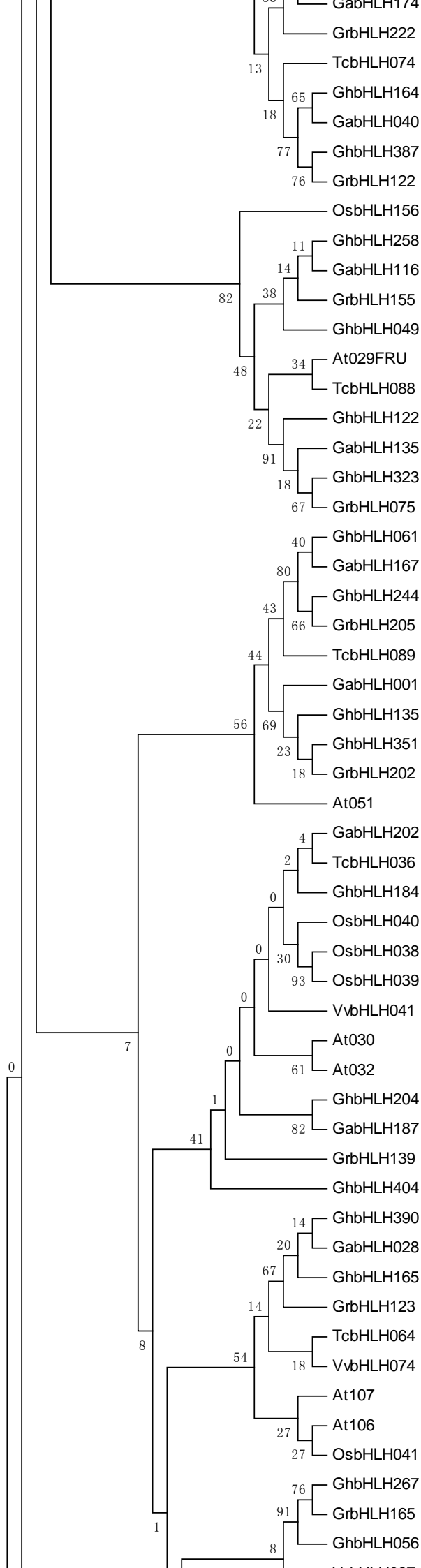

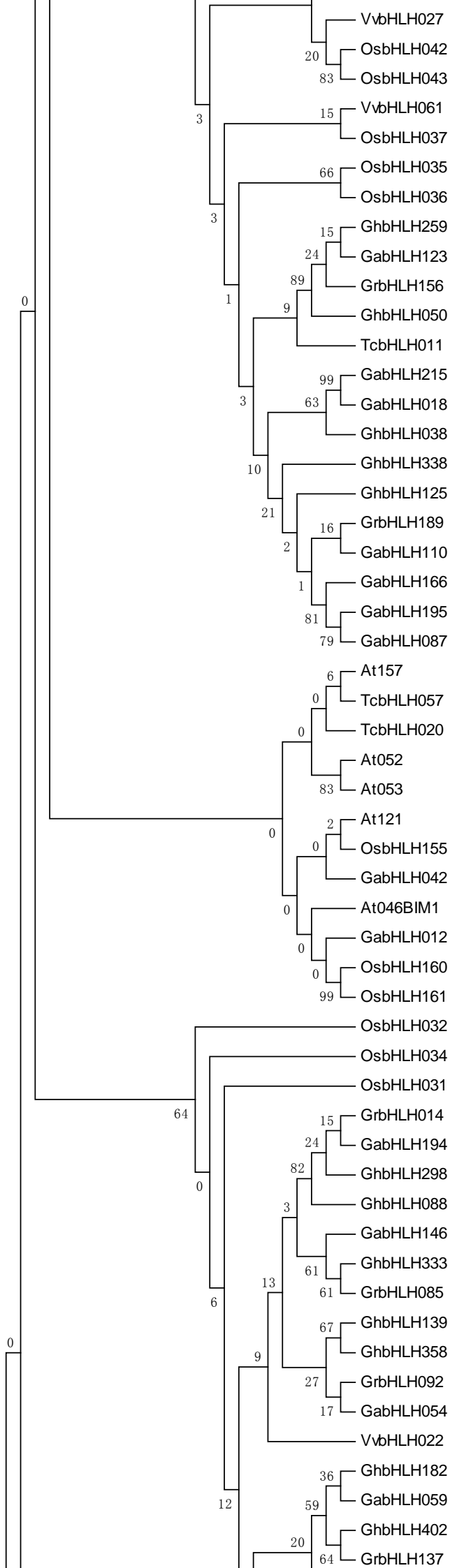

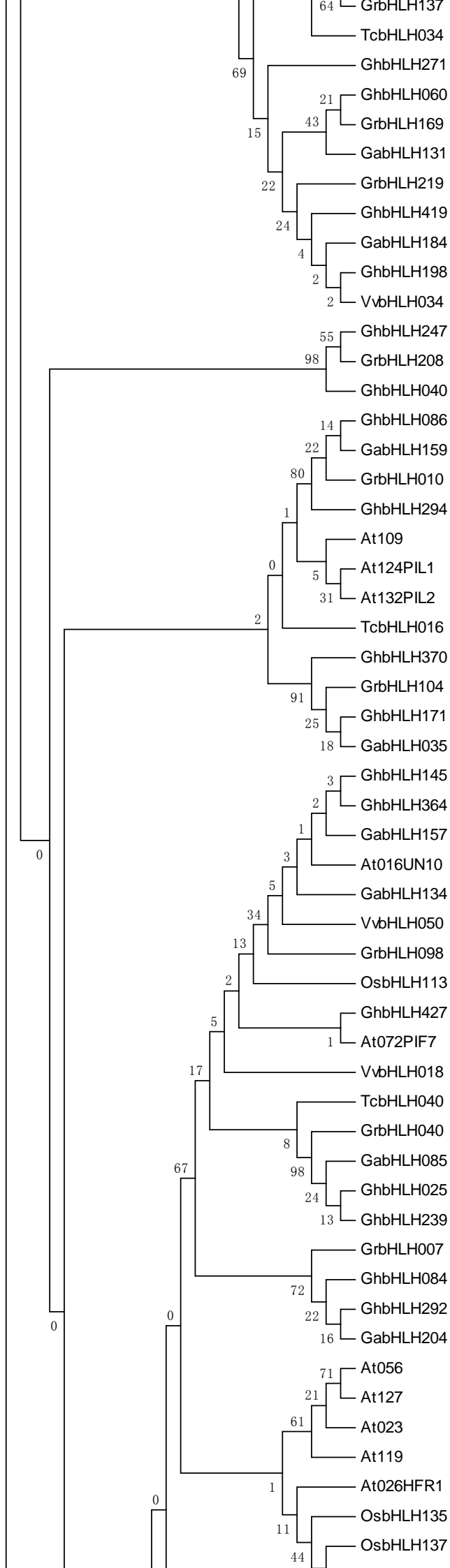

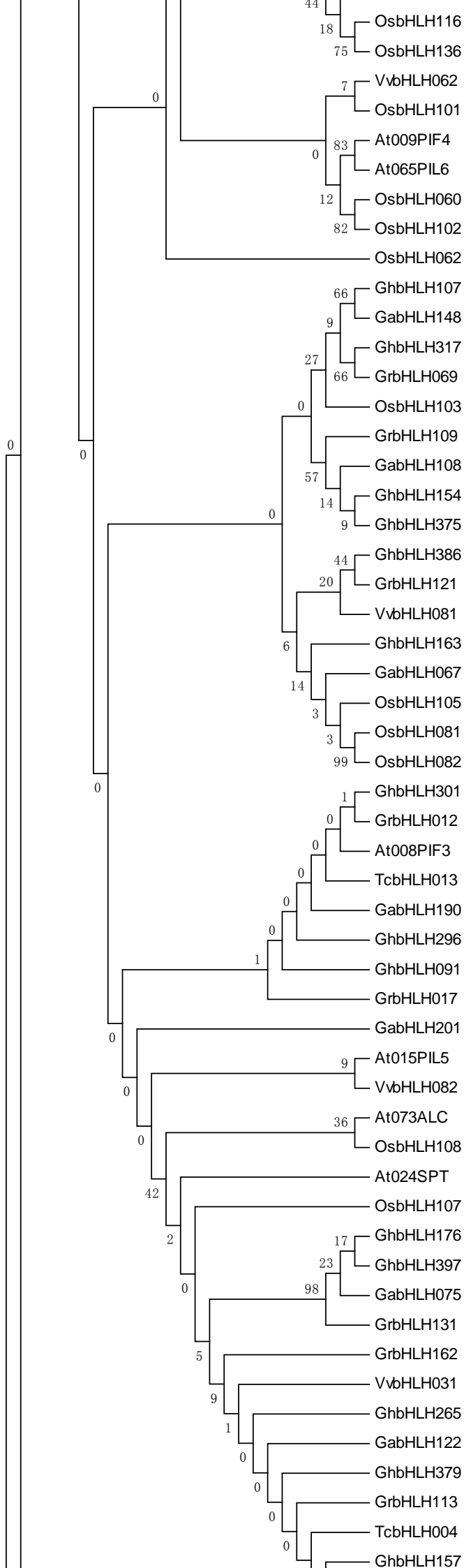

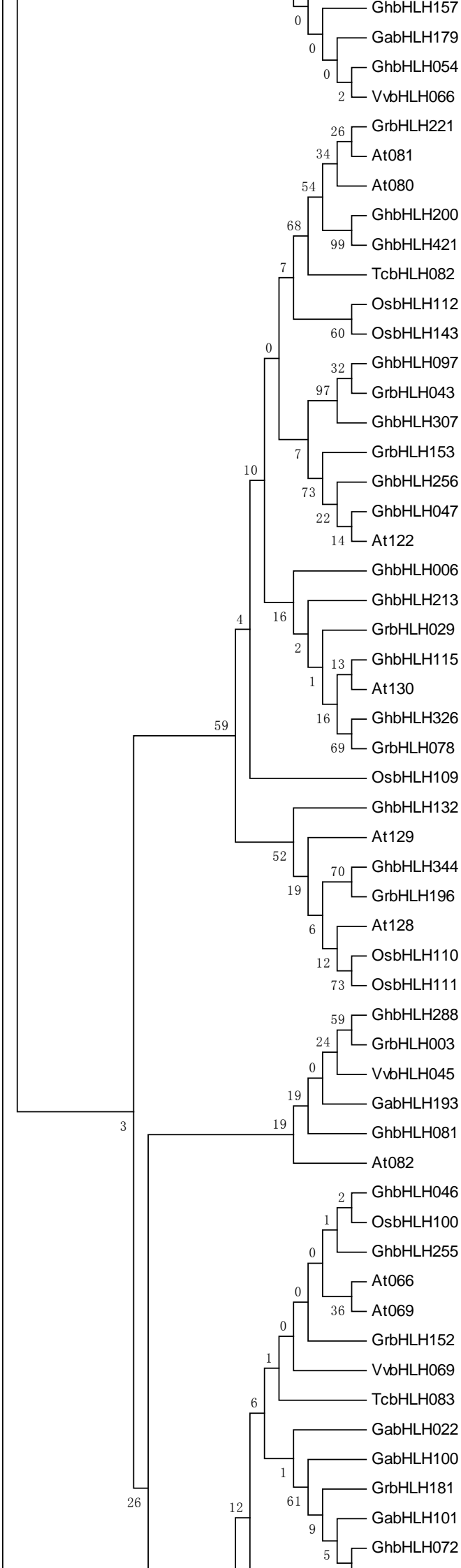

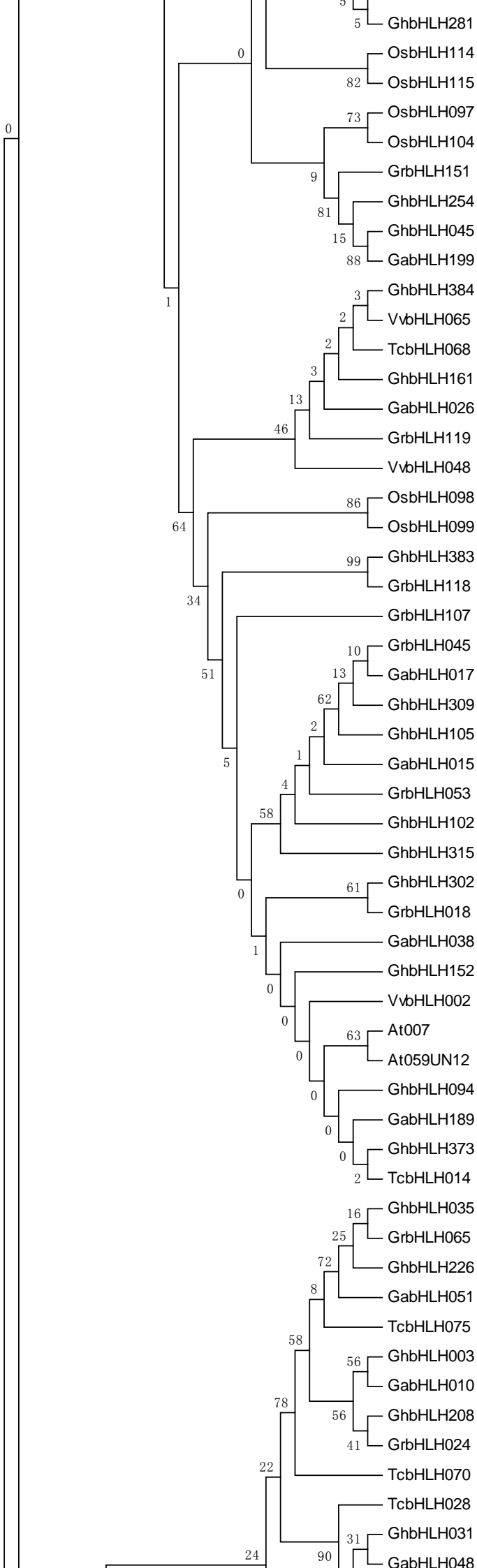

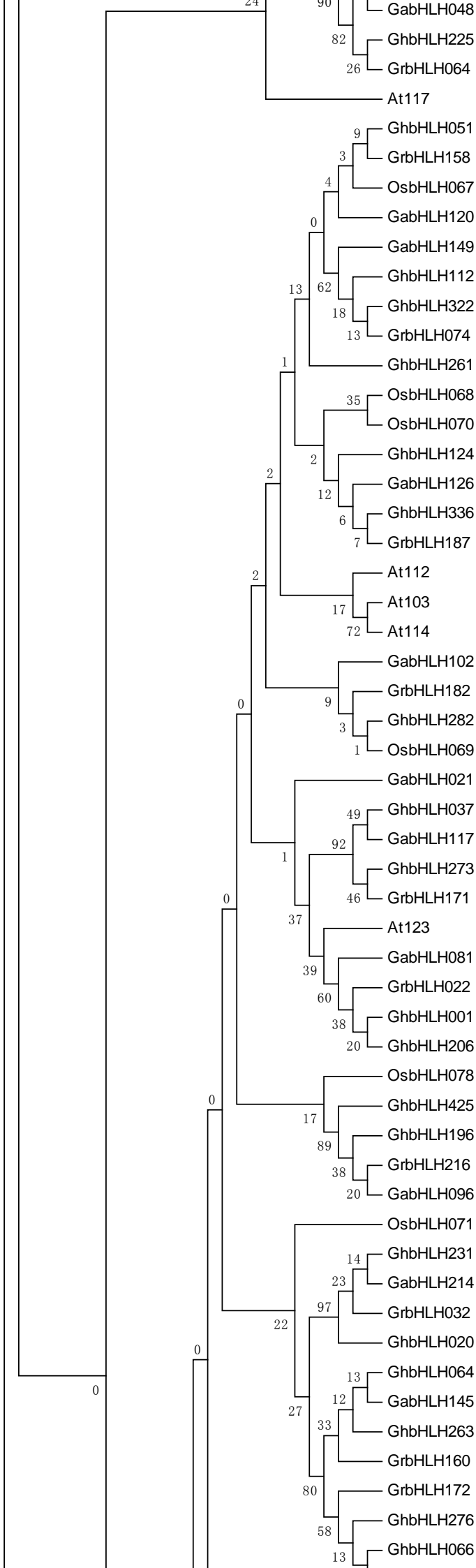

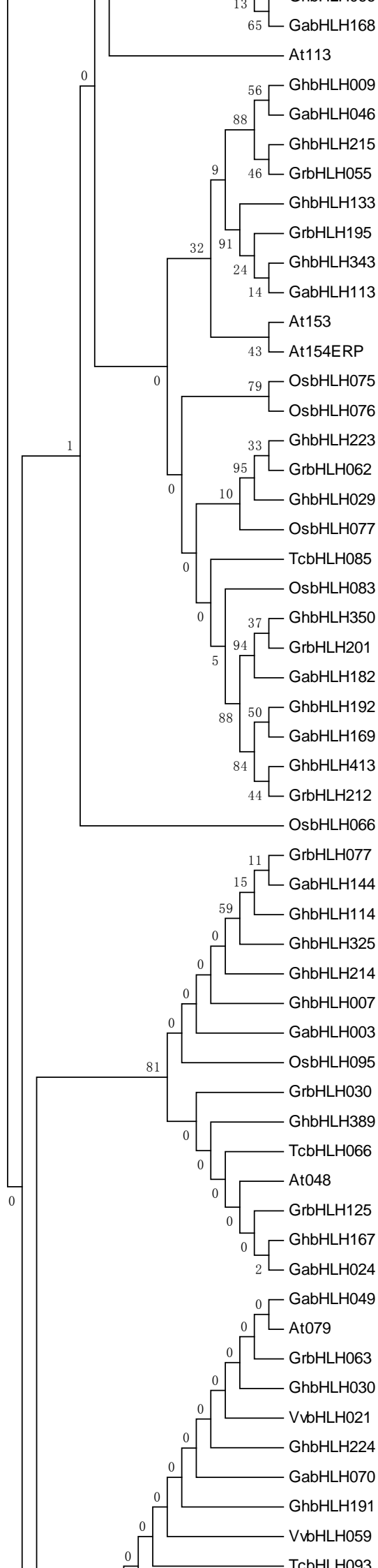

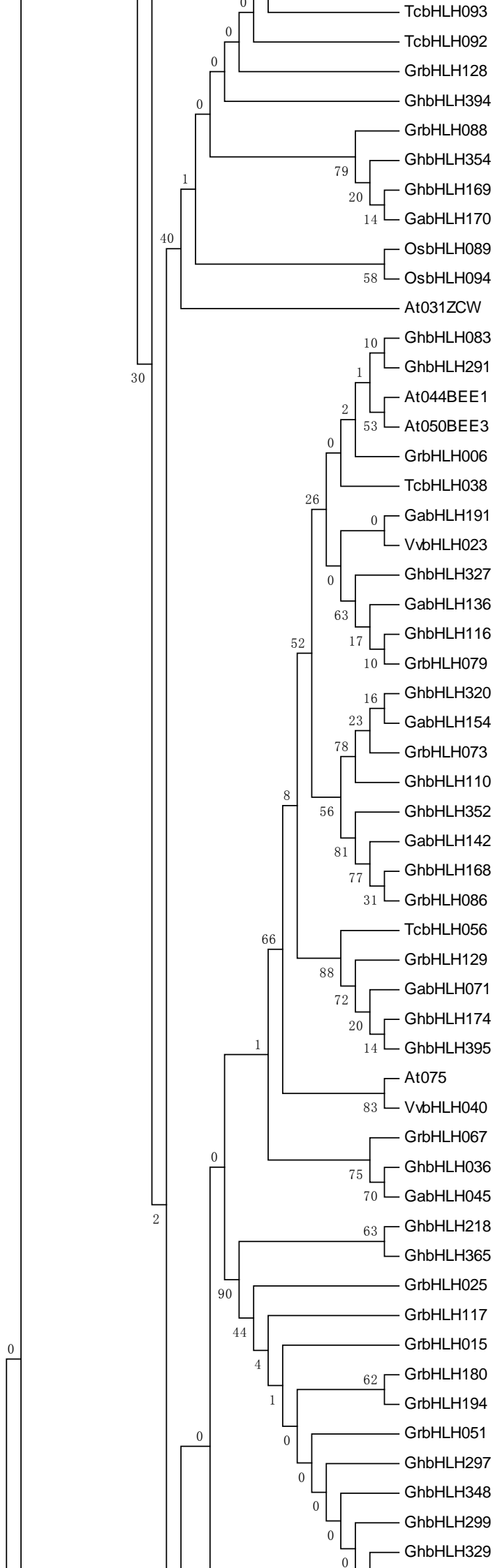

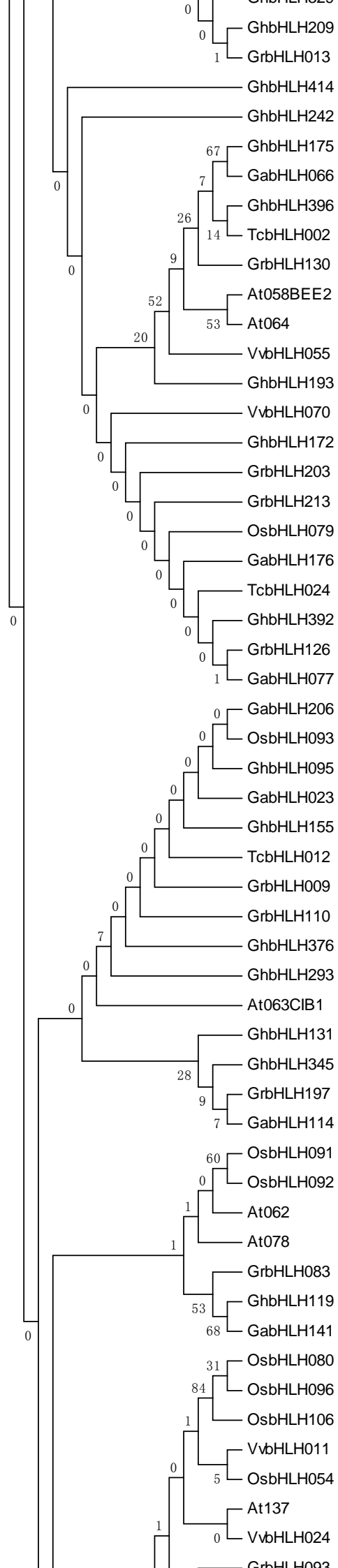

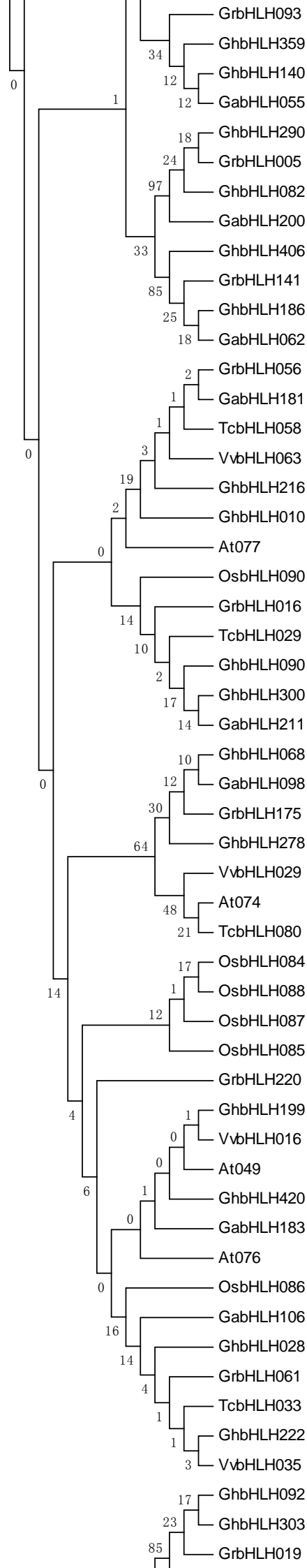

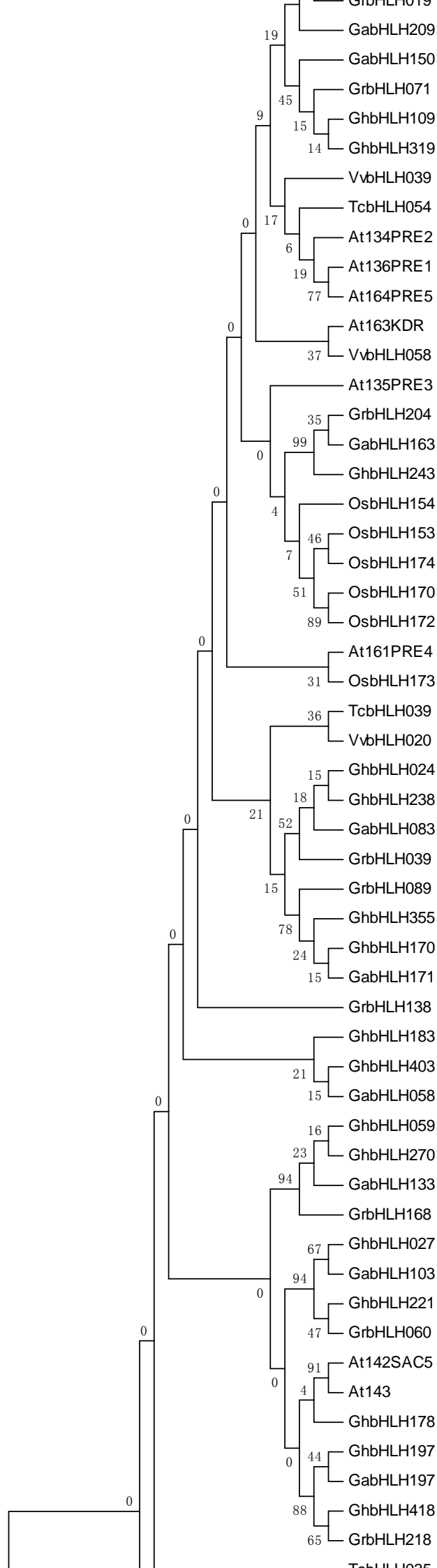

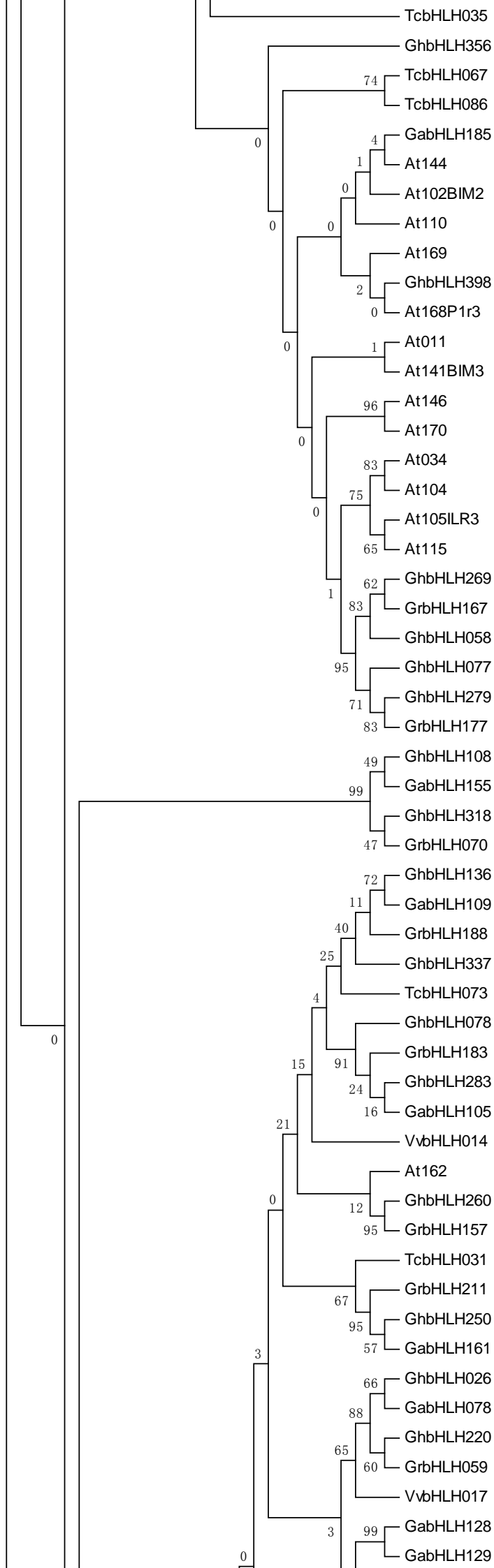

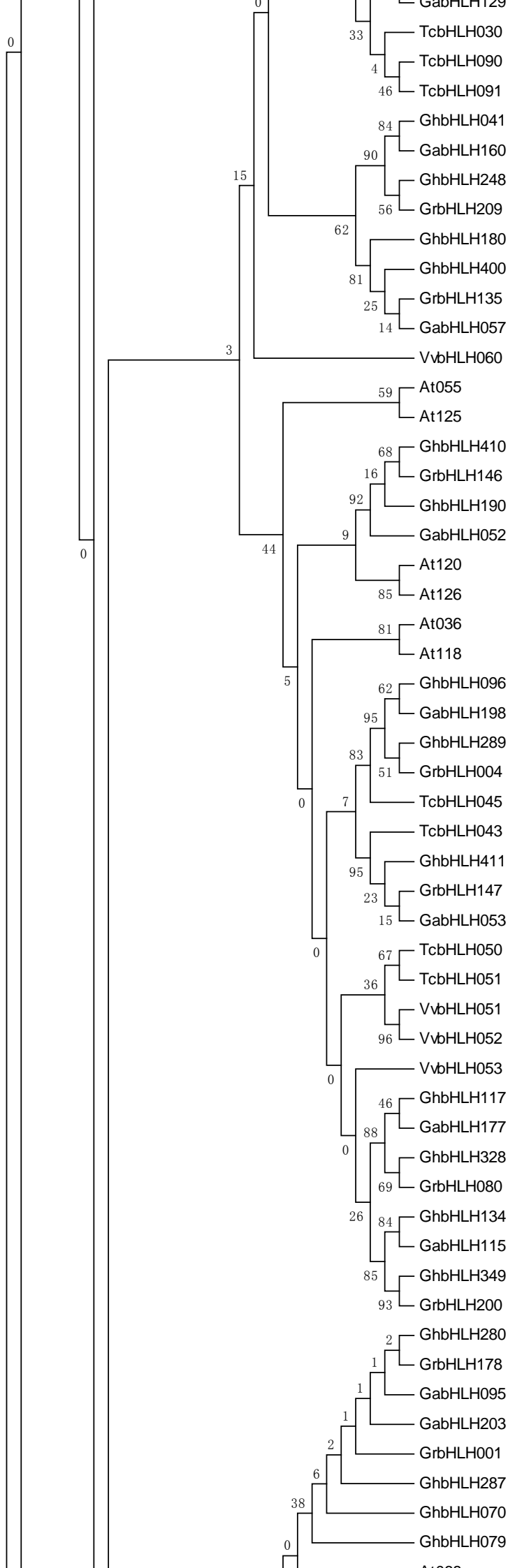

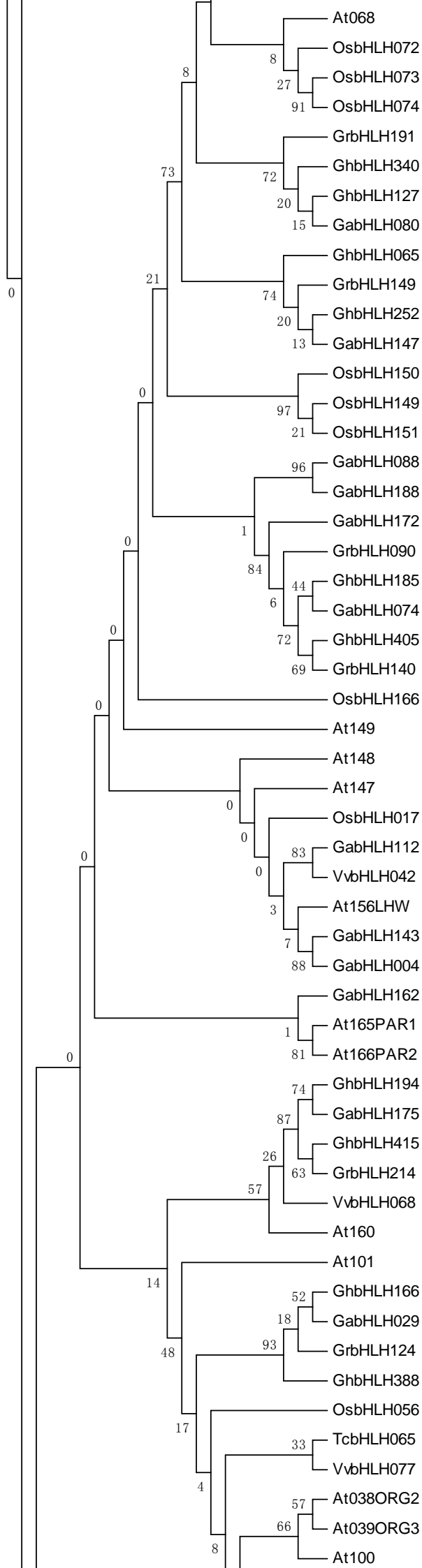

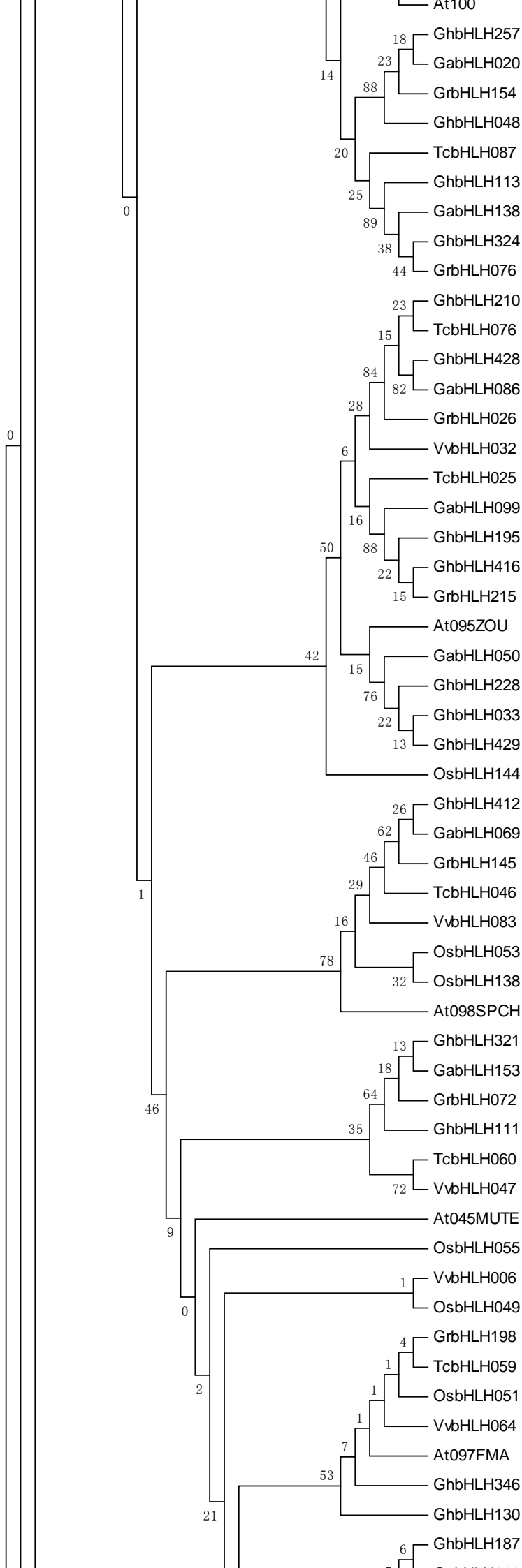

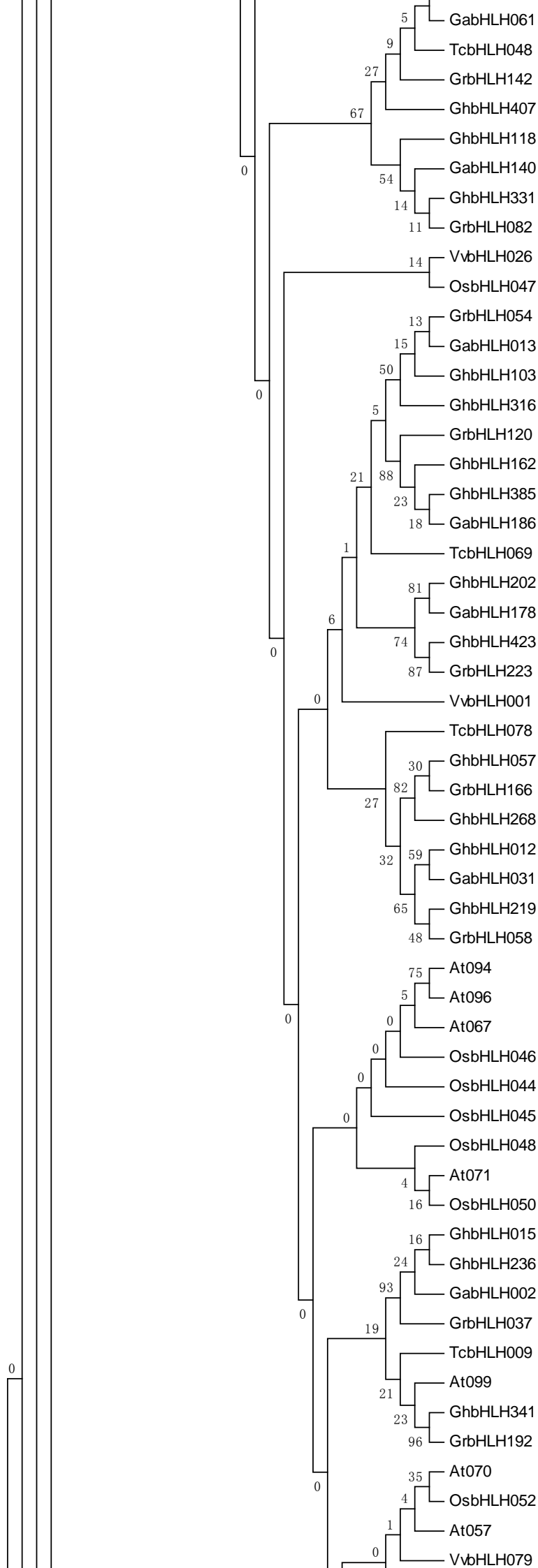

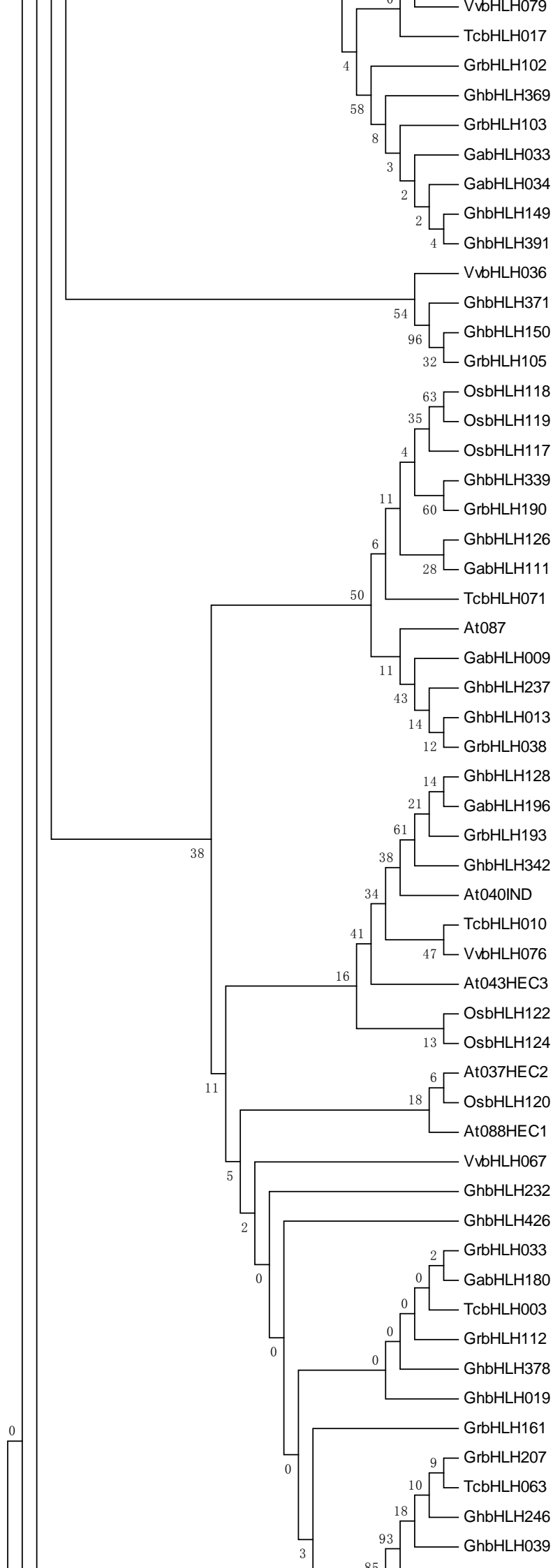

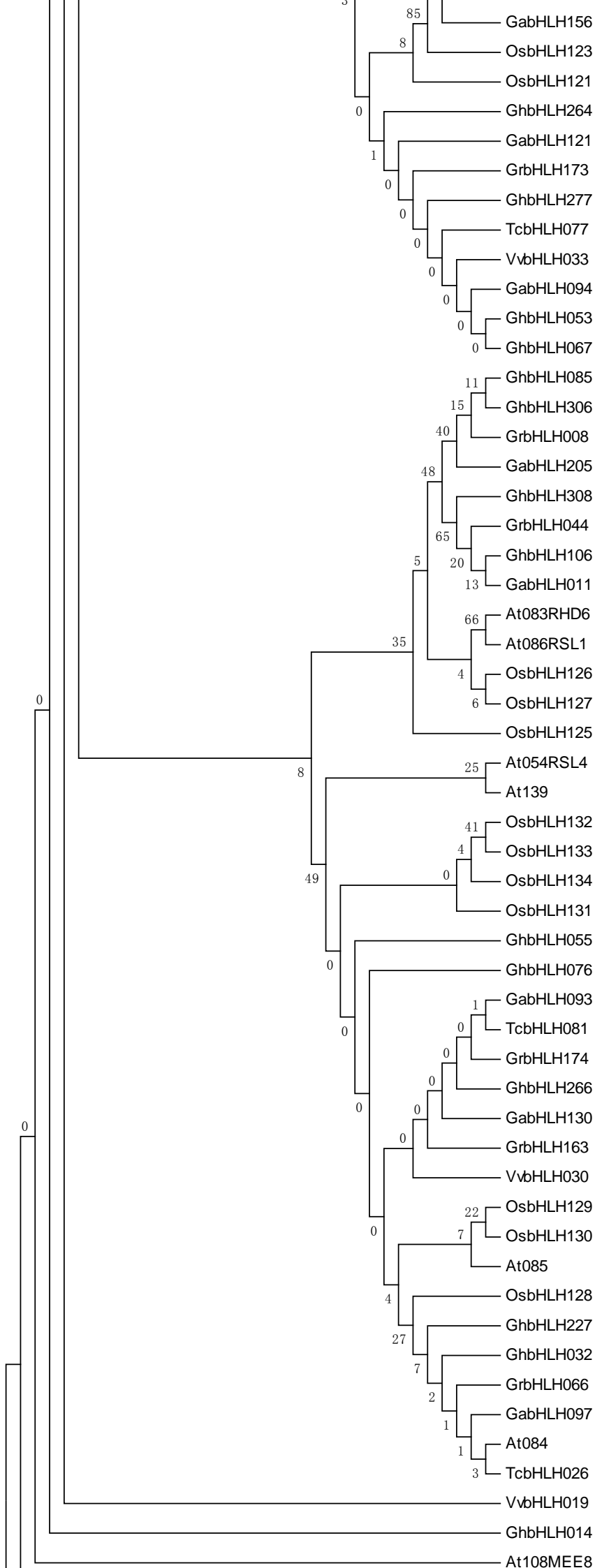

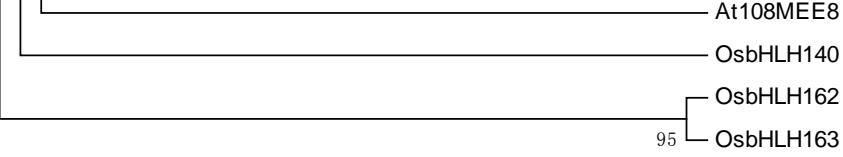

Supplement: Supplementary file 10 — MP phylogenetic tree of the bHLH members in cotton and other species. (PDF 103 kb) [file 12864_2018_4543_MOESM10_ESM.pdf]

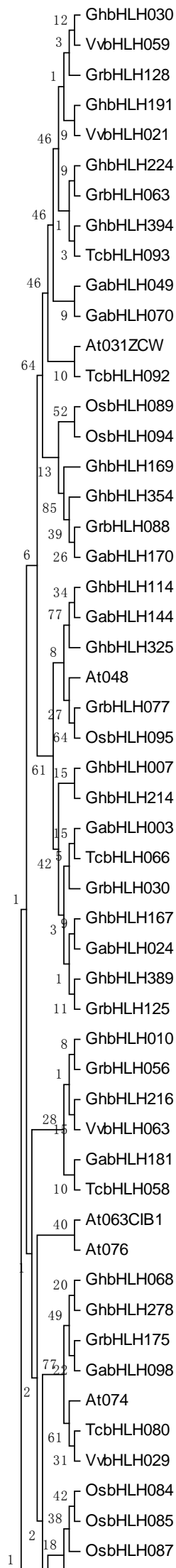

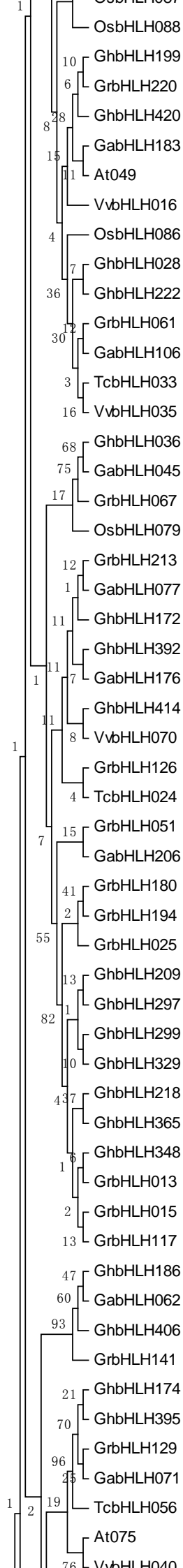

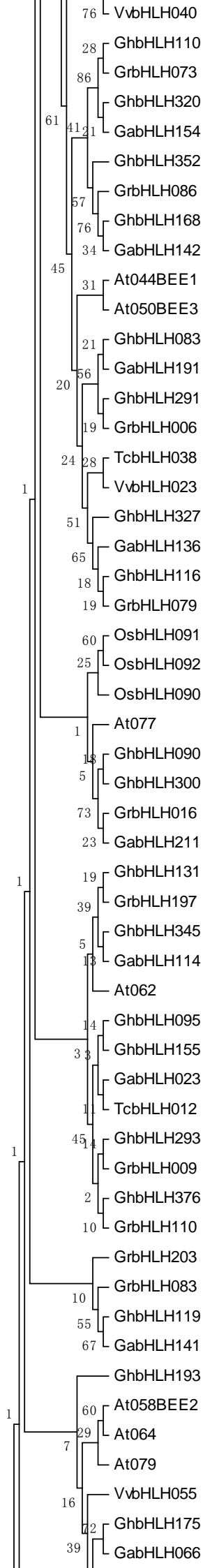

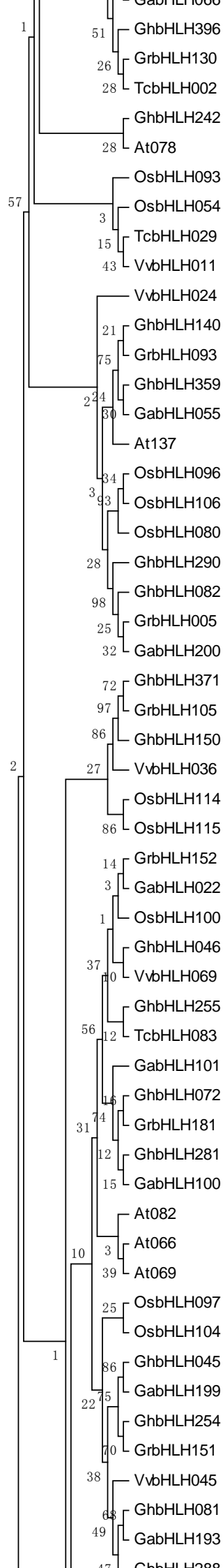

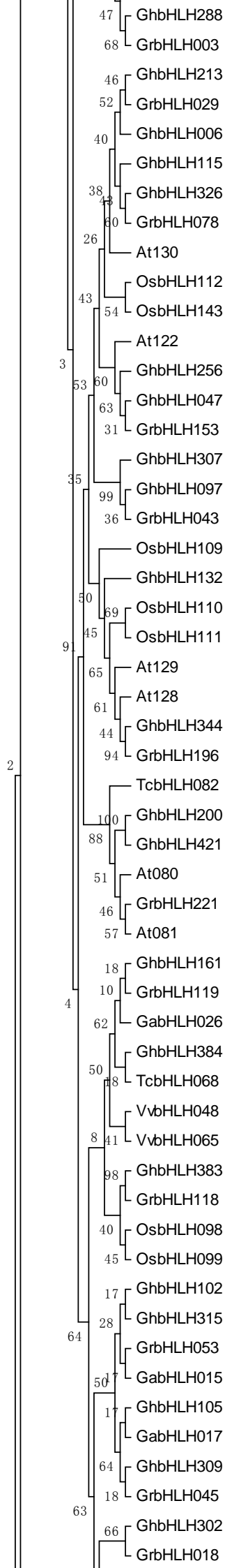

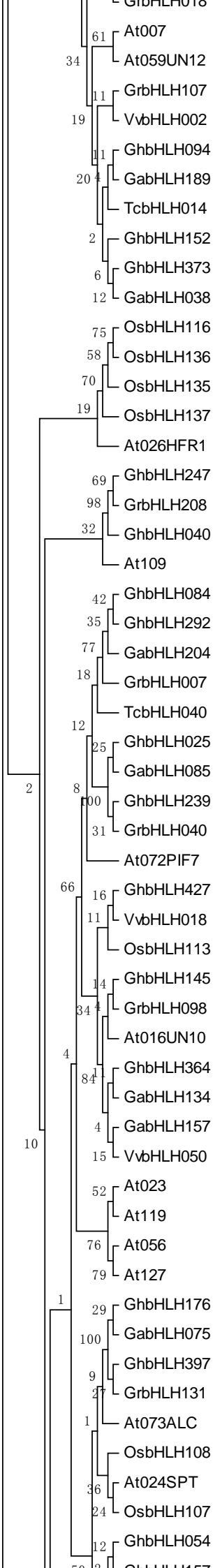

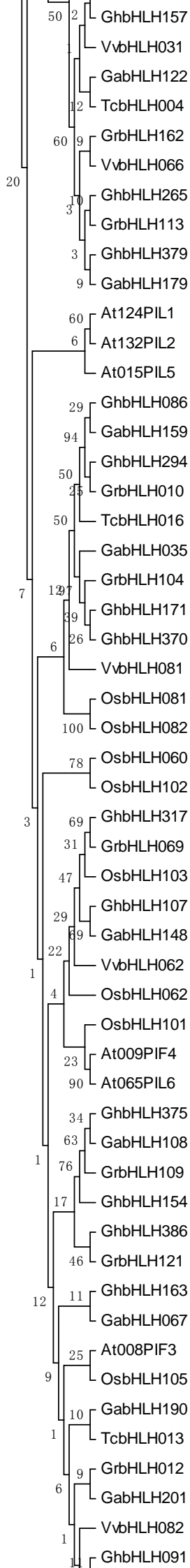

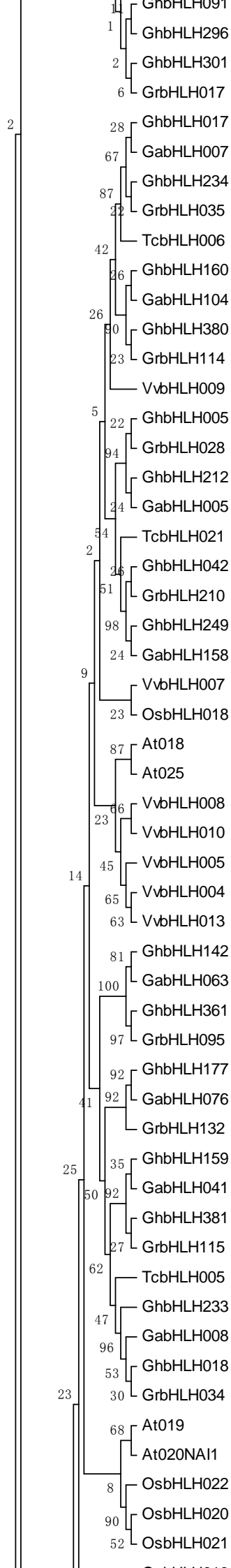

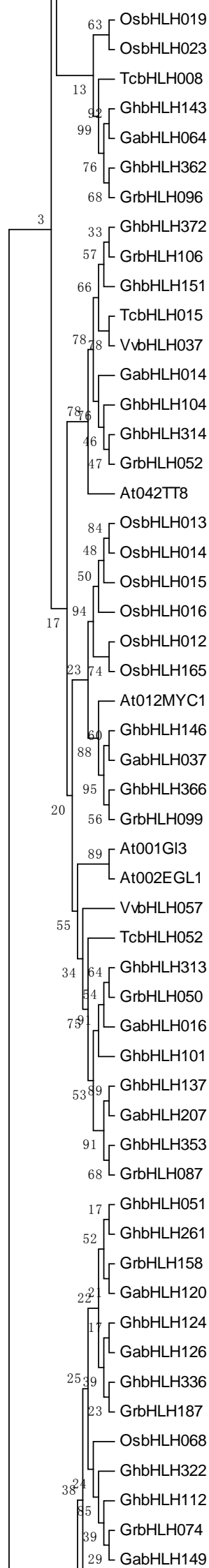

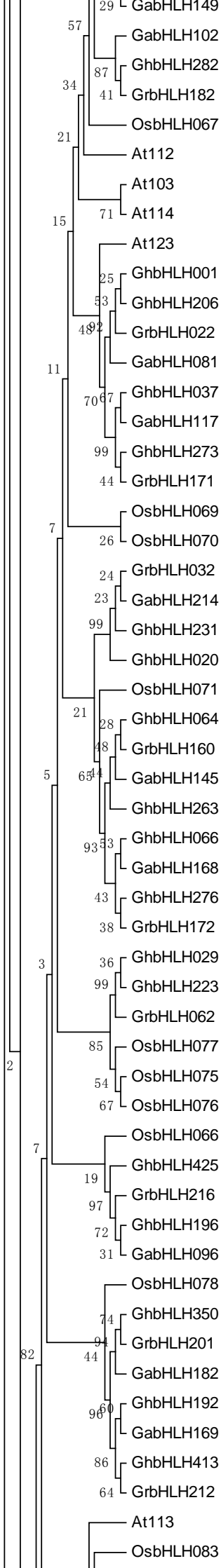

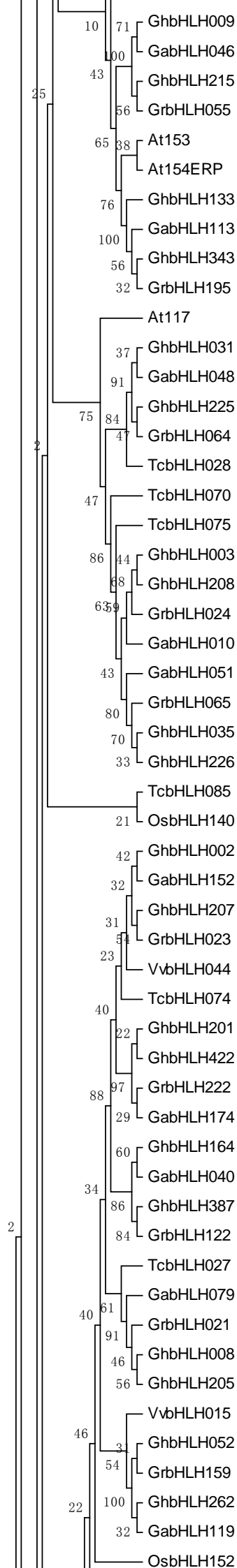

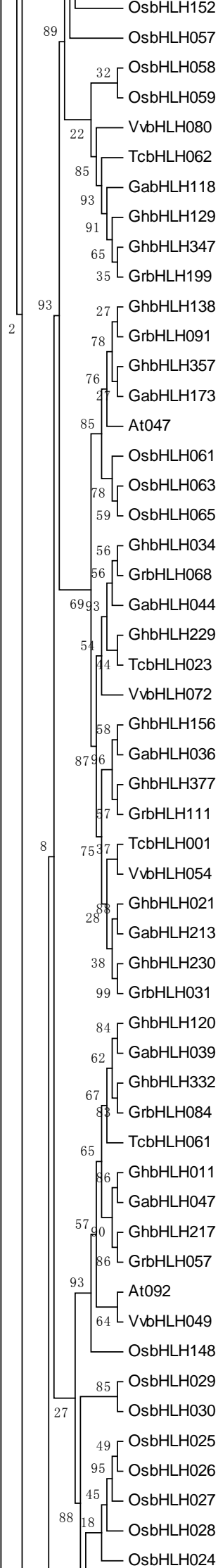

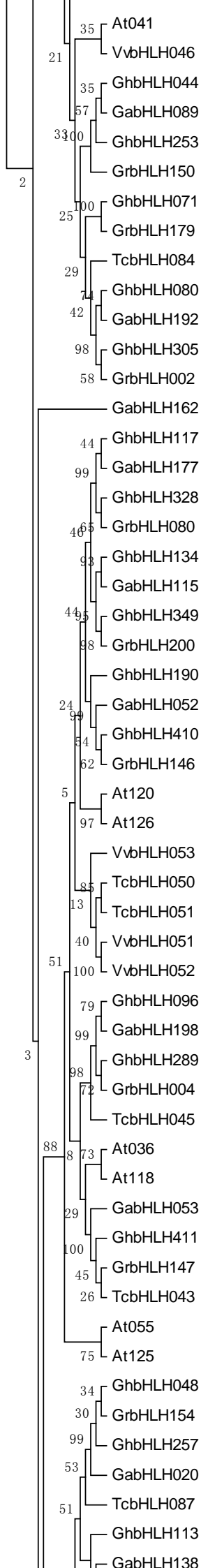

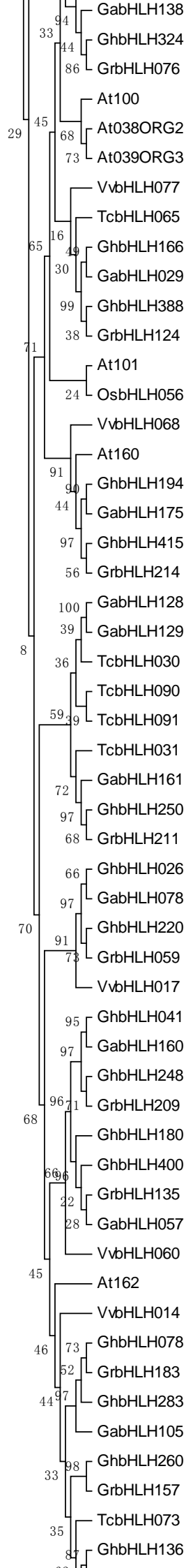

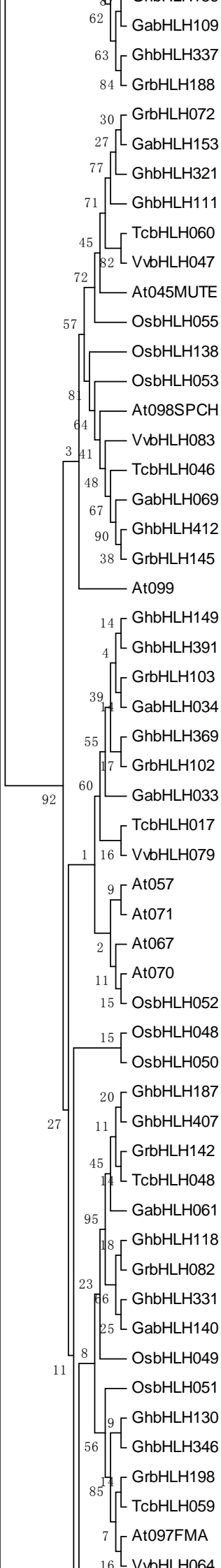

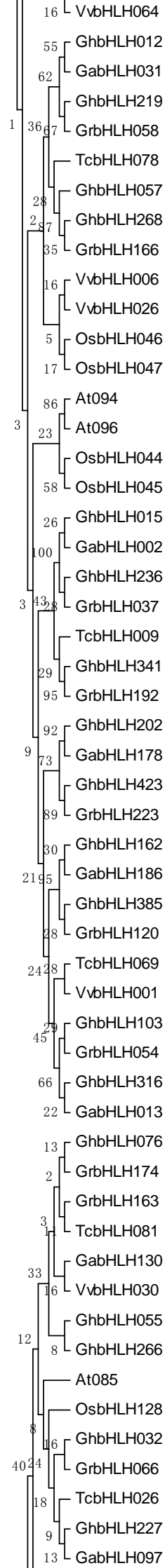

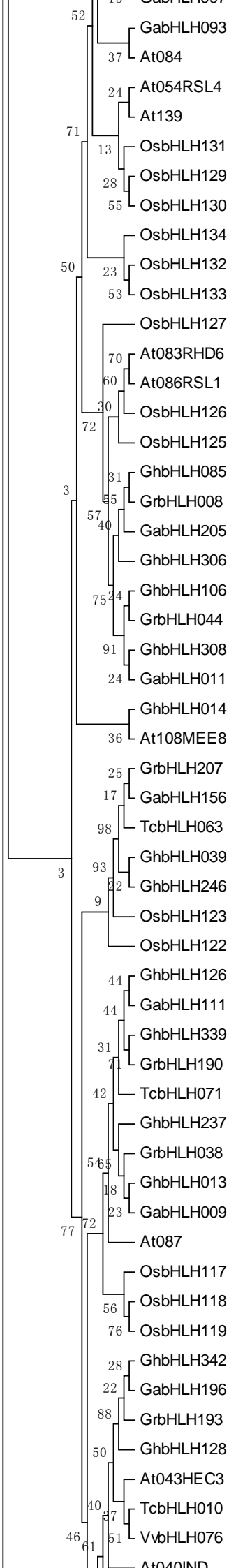

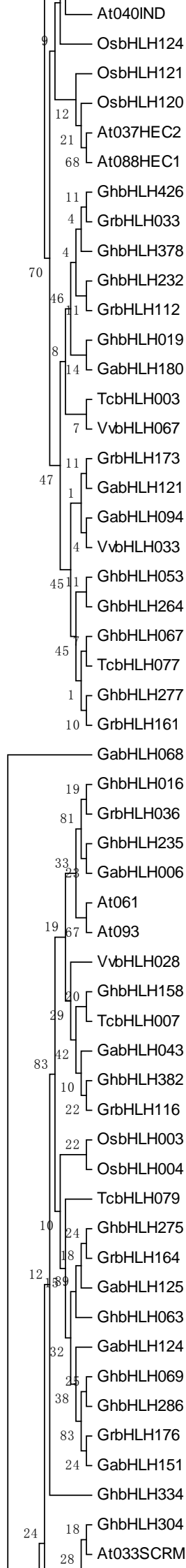

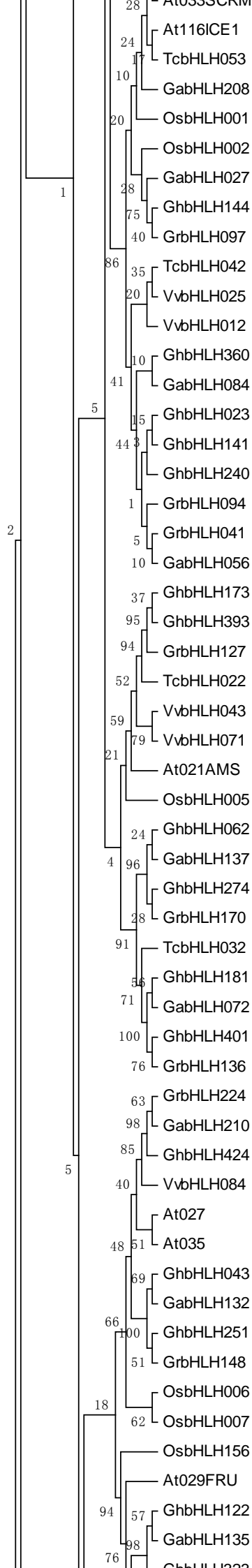

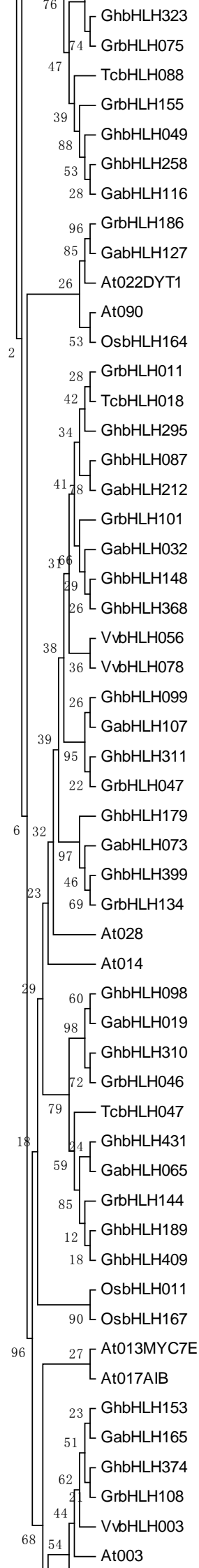

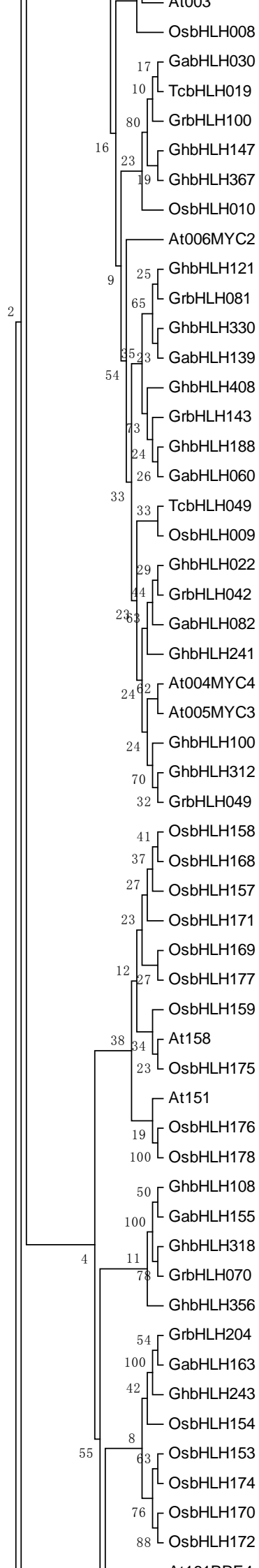

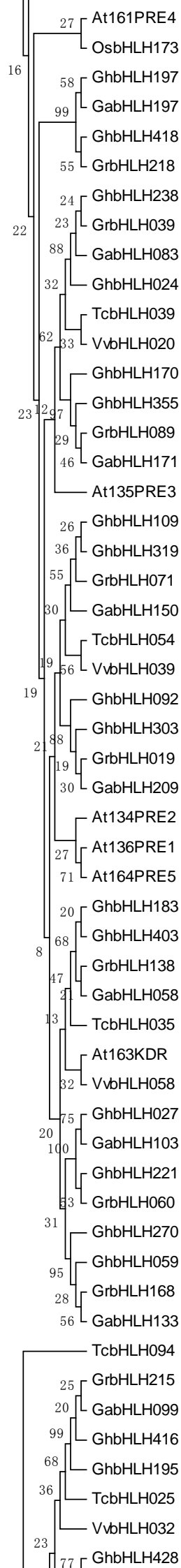

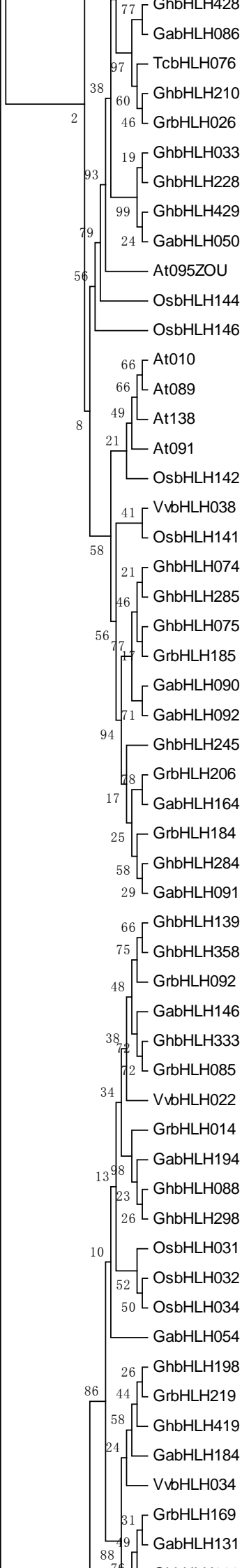

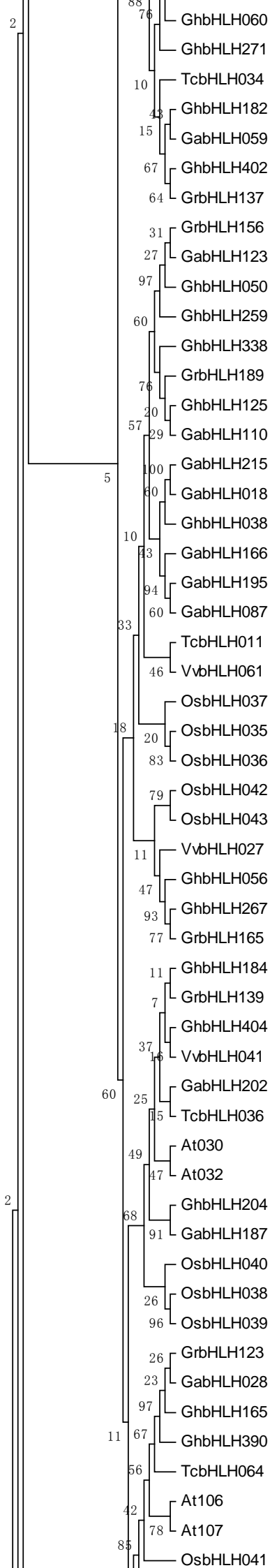

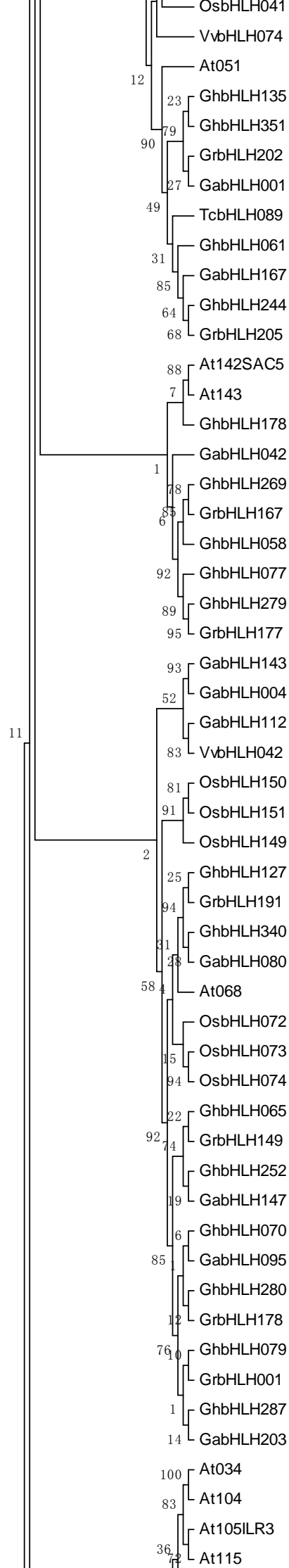

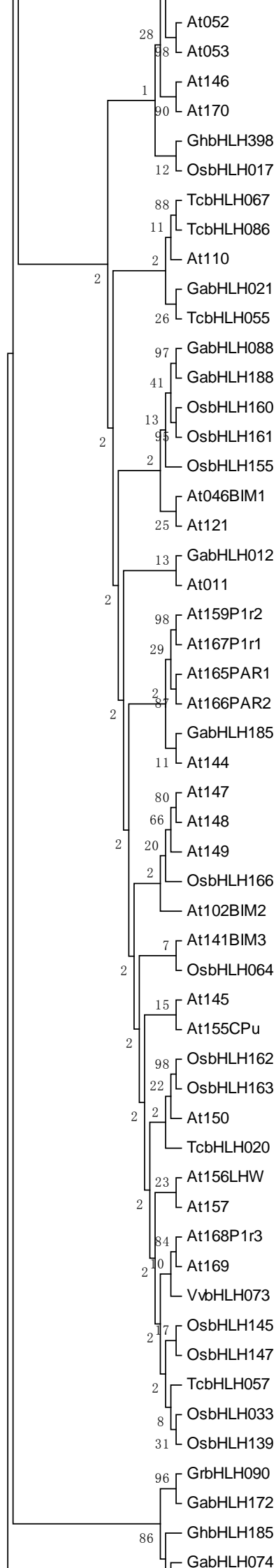

4  
83  
44  
66  
VvbHLH019  
GabHLH074  
GhbHLH405  
GrbHLH140

Supplement: Supplementary file 11 — ML phylogenetic tree of the bHLH members in cotton and other species. (PDF 102 kb) [file 12864_2018_4543_MOESM11_ESM.pdf]
