# Supplementary material for: Divergence and evolution of cotton bHLH proteins from diploid to allotetraploid
Source: BMC Genomics. 2018 Feb 23;19:162. doi: 10.1186/s12864-018-4543-y (PMC5824590; doi:10.1186/s12864-018-4543-y)
Supplement: Supplementary file 6 — NJ phylogenetic tree of the bHLH members in cotton and other species. (PDF 623 kb) [file 12864_2018_4543_MOESM6_ESM.pdf]

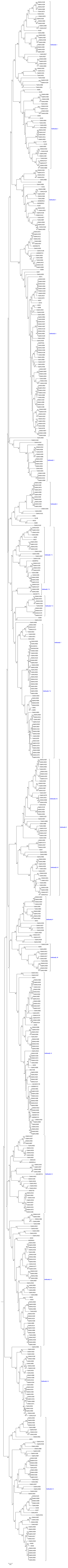

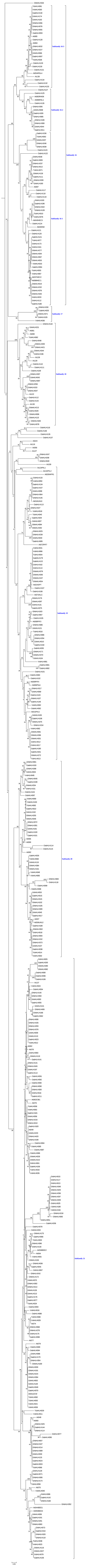

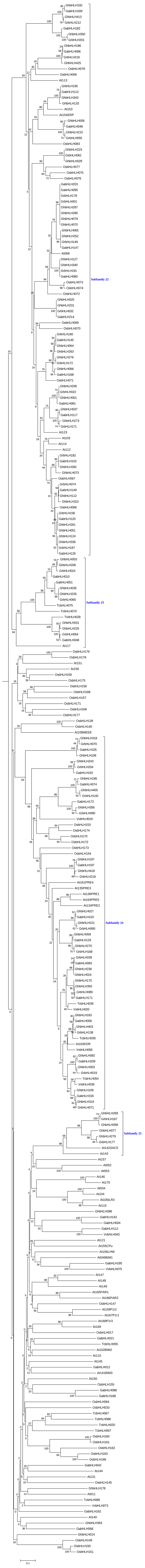
Subfamily 22
Subfamily 23
Subfamily 24
Subfamily 25
0.1
